# Multimodal Machine Learning Workflows for Prediction of Psychosis in Patients With Clinical High-Risk Syndromes and Recent-Onset Depression

**DOI:** 10.1001/jamapsychiatry.2020.3604

**Published:** 2020-12-02

**Authors:** Nikolaos Koutsouleris, Dominic B. Dwyer, Franziska Degenhardt, Carlo Maj, Maria Fernanda Urquijo-Castro, Rachele Sanfelici, David Popovic, Oemer Oeztuerk, Shalaila S. Haas, Johanna Weiske, Anne Ruef, Lana Kambeitz-Ilankovic, Linda A. Antonucci, Susanne Neufang, Christian Schmidt-Kraepelin, Stephan Ruhrmann, Nora Penzel, Joseph Kambeitz, Theresa K. Haidl, Marlene Rosen, Katharine Chisholm, Anita Riecher-Rössler, Laura Egloff, André Schmidt, Christina Andreou, Jarmo Hietala, Timo Schirmer, Georg Romer, Petra Walger, Maurizia Franscini, Nina Traber-Walker, Benno G. Schimmelmann, Rahel Flückiger, Chantal Michel, Wulf Rössler, Oleg Borisov, Peter M. Krawitz, Karsten Heekeren, Roman Buechler, Christos Pantelis, Peter Falkai, Raimo K. R. Salokangas, Rebekka Lencer, Alessandro Bertolino, Stefan Borgwardt, Markus Noethen, Paolo Brambilla, Stephen J. Wood, Rachel Upthegrove, Frauke Schultze-Lutter, Anastasia Theodoridou, Eva Meisenzahl

**Affiliations:** 1 Department of Psychiatry and Psychotherapy, Ludwig-Maximilian-University, Munich, Germany; 2 Max-Planck Institute of Psychiatry, Munich, Germany; 3 Institute of Psychiatry, Psychology and Neuroscience, King’s College London, London, United Kingdom; 4 Institute of Human Genetics, Rheinische Friedrich-Wilhelms-Universität Bonn, Bonn, Germany; 5 Department of Child and Adolescent Psychiatry, Psychosomatics and Psychotherapy, University Hospital Essen, University of Duisburg-Essen, Essen, Germany; 6 Institute of Genomic Statistics and Bioinformatics, University of Bonn, Bonn, Germany; 7 Max-Planck School of Cognition, Leipzig, Germany; 8 International Max-Planck Research School for Translational Psychiatry, Munich, Germany; 9 Department of Psychiatry, Icahn School of Medicine at Mount Sinai, New York, New York; 10 Department of Psychiatry and Psychotherapy, Faculty of Medicine and University Hospital, University of Cologne, Cologne, Germany; 11 Department of Education, Psychology, and Communication, University of Bari Aldo Moro, Bari, Italy; 12 Department of Psychiatry and Psychotherapy, Medical Faculty, Heinrich-Heine University, Düsseldorf, Germany; 13 Institute for Mental Health, University of Birmingham, Birmingham, United Kingdom; 14 Department of Psychiatry, Psychiatric University Hospital, University of Basel, Switzerland; 15 Department of Psychiatry, University of Turku, Turku, Finland; 16 GE Healthcare GmbH (previously GE Global Research GmbH), Munich, Germany; 17 Department of Child and Adolescent Psychiatry, University of Münster, Münster, Germany; 18 Department of Child and Adolescent Psychiatry, Psychotherapy and Psychosomatics, LVR Clinic Düsseldorf, Düsseldorf, Germany; 19 Department of Child and Adolescent Psychiatry and Psychotherapy, University of Zürich, Zürich, Switzerland; 20 University Hospital of Child and Adolescent Psychiatry, University Hospital Hamburg-Eppendorf, Hamburg, Germany; 21 University Hospital of Child and Adolescent Psychiatry and Psychotherapy, University of Bern, Bern, Switzerland; 22 Department of Psychiatry, Psychotherapy and Psychosomatics, University Hospital of Psychiatry Zurich, Zurich, Switzerland; 23 Department of Psychiatry and Psychotherapy I, LVR Hospital Cologne, Cologne, Germany; 24 Department of Neuroradiology, University Hospital of Zurich, Zurich, Switzerland; 25 Melbourne Neuropsychiatry Centre, University of Melbourne and Melbourne Health, Melbourne, Australia; 26 Department of Psychiatry and Psychotherapy, University of Münster, Münster, Germany; 27 Department of Psychiatry and Psychotherapy, University of Lübeck, Lübeck, Germany; 28 Department of Basic Medical Science, Neuroscience and Sense Organs, University of Bari Aldo Moro, Bari, Italy; 29 Department of Neurosciences and Mental Health, Fondazione Istituto di Ricovero e Cura a Carattere Scientifico Ca’ Granda Ospedale Maggiore Policlinico, Milan, Italy; 30 Department of Pathophysiology and Transplantation, University of Milan, Milan, Italy; 31 Centre for Youth Mental Health, University of Melbourne, Melbourne, Australia; 32 Orygen, the National Centre of Excellence for Youth Mental Health, Melbourne, Australia; 33 Department of Psychology and Mental Health, Faculty of Psychology, Airlangga University, Surabaya, Indonesia

## Abstract

**Importance:**

Diverse models have been developed to predict psychosis in patients with clinical high-risk (CHR) states. Whether prediction can be improved by efficiently combining clinical and biological models and by broadening the risk spectrum to young patients with depressive syndromes remains unclear.

**Objectives:**

To evaluate whether psychosis transition can be predicted in patients with CHR or recent-onset depression (ROD) using multimodal machine learning that optimally integrates clinical and neurocognitive data, structural magnetic resonance imaging (sMRI), and polygenic risk scores (PRS) for schizophrenia; to assess models’ geographic generalizability; to test and integrate clinicians’ predictions; and to maximize clinical utility by building a sequential prognostic system.

**Design, Setting, and Participants:**

This multisite, longitudinal prognostic study performed in 7 academic early recognition services in 5 European countries followed up patients with CHR syndromes or ROD and healthy volunteers. The referred sample of 167 patients with CHR syndromes and 167 with ROD was recruited from February 1, 2014, to May 31, 2017, of whom 26 (23 with CHR syndromes and 3 with ROD) developed psychosis. Patients with 18-month follow-up (n = 246) were used for model training and leave-one-site-out cross-validation. The remaining 88 patients with nontransition served as the validation of model specificity. Three hundred thirty-four healthy volunteers provided a normative sample for prognostic signature evaluation. Three independent Swiss projects contributed a further 45 cases with psychosis transition and 600 with nontransition for the external validation of clinical-neurocognitive, sMRI-based, and combined models. Data were analyzed from January 1, 2019, to March 31, 2020.

**Main Outcomes and Measures:**

Accuracy and generalizability of prognostic systems.

**Results:**

A total of 668 individuals (334 patients and 334 controls) were included in the analysis (mean [SD] age, 25.1 [5.8] years; 354 [53.0%] female and 314 [47.0%] male). Clinicians attained a balanced accuracy of 73.2% by effectively ruling out (specificity, 84.9%) but ineffectively ruling in (sensitivity, 61.5%) psychosis transition. In contrast, algorithms showed high sensitivity (76.0%-88.0%) but low specificity (53.5%-66.8%). A cybernetic risk calculator combining all algorithmic and human components predicted psychosis with a balanced accuracy of 85.5% (sensitivity, 84.6%; specificity, 86.4%). In comparison, an optimal prognostic workflow produced a balanced accuracy of 85.9% (sensitivity, 84.6%; specificity, 87.3%) at a much lower diagnostic burden by sequentially integrating clinical-neurocognitive, expert-based, PRS-based, and sMRI-based risk estimates as needed for the given patient. Findings were supported by good external validation results.

**Conclusions and Relevance:**

These findings suggest that psychosis transition can be predicted in a broader risk spectrum by sequentially integrating algorithms’ and clinicians’ risk estimates. For clinical translation, the proposed workflow should undergo large-scale international validation.

## Key Points

**Question:**

Can a transition to psychosis be predicted in patients with clinical high-risk states or recent-onset depression by optimally integrating clinical, neurocognitive, neuroimaging, and genetic information with clinicians’ prognostic estimates?

**Findings:**

In this prognostic study of 334 patients and 334 control individuals, machine learning models sequentially combining clinical and biological data with clinicians’ estimates correctly predicted disease transitions in 85.9% of cases across geographically distinct patient populations. The clinicians’ lack of prognostic sensitivity, as measured by a false-negative rate of 38.5%, was reduced to 15.4% by the sequential prognostic model.

**Meaning:**

These findings suggest that an individualized prognostic workflow integrating artificial and human intelligence may facilitate the personalized prevention of psychosis in young patients with clinical high-risk syndromes or recent-onset depression.

## Teaser

This prognostic study evaluates whether psychosis transition can be predicted in patients with clinical high-risk syndromes or recent-onset depression by multimodal machine learning that optimally integrates clinical and neurocognitive data, structural magnetic resonance imaging, and polygenic risk scores for schizophrenia.

## Introduction

The clinical high-risk (CHR) criteria for psychosis have been established to detect vulnerable individuals as early as possible to intercept disease development.^1^ These criteria identify a patient population with increased incidence compared with the general population,^2^ yet only 22% of patients with CHR as detected by ultra–high-risk criteria show a psychosis transition during a 3-year period.^2^ The clinical utility of the CHR designation may be further limited because its ascertainment is laborious and confined to specialized, well-equipped health care services that do not sufficiently cover the vulnerable population.^3,4^ Hence, improved prognostic accuracy and clinical scalability are needed to accurately identify patients truly at risk for psychosis.

Prognostic accuracy may be increased using psychosis risk calculators for populations with CHR based on conventional statistics^3,5,6,7^ or machine learning,^8,9,10,11,12,13^ with studies finding that a first episode can be predicted with clinical data,^3,14^ combinations of clinical and cognitive data,^6,15^ neuroimaging,^8^ and, recently, with polygenic risk scores (PRS) for schizophrenia,^16^ among other measures.^17^ However, reviews^18,19,20^ have also highlighted methodological shortcomings, such as inadequate sample sizes and model validation strategies,^21,22^ that may have inflated accuracy. Moreover, studies suggested that psychosis does not only emerge from CHR states^23,24^ but occurs and can be predicted across a broader spectrum of comorbid conditions commencing in late adolescence and early adulthood.^3,14^ Hence, generalizable risk prediction models may require transdiagnostic discovery and validation populations, encompassing patients with CHR states and early-onset affective syndromes that share environmental, clinical, and neurobiological features.^25,26,27,28,29,30^ In addition, the growing diversity of risk prediction models originating from different data modalities has led to uncertainty about the minimum number of modalities needed to increase prognostic accuracy to a level justifying clinical implementation.^2,31^ Finally, algorithms should be compared and integrated with clinicians’ predictions of psychosis transition to determine their potential utility from public health and service provision perspectives.^13,32^

Addressing these challenges, the European Union–funded PRONIA study (Personalised Prognostic Tools for Early Psychosis Management [https://www.pronia.eu]) collected multimodal longitudinal data from adolescents and young adults in CHR states, those with recent-onset depression (ROD), and healthy control individuals. We evaluated clinical, neuroanatomical, and genetic machine learning models trained to identify patients with CHR syndromes and ROD who undergo psychosis transition. We compared our models’ performance with our clinical raters’ ability to predict psychosis transition and explored whether the sequential integration of algorithmic and expert-based prognoses produces clinically efficient cybernetic workflows,^33,34^ that is, structured interactions between humans and machines that maximize prognostic accuracy while minimizing the examination burden in the given patient. We assessed potential confounders and moderators of prognostic performance and tested whether our models’ and raters’ estimates predicted not only psychosis transition but also distinct CHR syndromes and nonpsychotic disease trajectories. Finally, we explored whether a considerably condensed, and hence less burdensome, clinical model could be generalized to 3 independent patient cohorts with CHR syndromes and other mental conditions.^35,36,37^ This external validation step also benchmarked the neuroanatomical and combined models derived from the PRONIA cohort.

## Methods

### Study Design and Population

The eMethods section in the Supplement details all of the methods for this prognostic study, which followed the Transparent Reporting of a Multivariable Prediction Model for Individual Prognosis or Diagnosis (TRIPOD) guideline.^38^ In summary, our analysis included 334 patients with CHR states (n = 167) or ROD (n = 167) recruited across 7 sites in Finland, Germany, Italy, Switzerland, and the United Kingdom from February 1, 2014, to May 31, 2017, using standardized inclusion and exclusion criteria (eTables 1 and 2 and CONSORT diagram in eFigure 1 in the Supplement). Follow-up for all patients ranged from 9 to 36 months,^13^ with visits every 3 months to the 18-month point and every 9 months thereafter. Furthermore, 334 healthy controls matched for age, sex, and site were included to evaluate prognostic patterns. Adult participants gave informed consent before study inclusion. Participants younger than 18 years and their guardians provided their written informed assent and consent. The PRONIA observational study was registered at the German Clinical Trials Register (DRKS00005042) and approved by all local research ethics committees.

Sociodemographic and clinical variables were compared between diagnostic groups (eTable 3 in the Supplement), patients with psychosis transition and nontransition, and patients with 18-month or later follow-up data (discovery sample [n = 246]; PRONIA plus 18M) or earlier attrition (validation sample [n = 88]; PRONIA minus 18M) (Table 1). Psychosis transition was defined when at least 1 of the 5 positive symptom items in the Structured Interview for Psychosis–Risk Syndromes^39^ reached psychotic intensity daily for at least 7 days.^40^ Diagnoses of cases with psychosis transition are listed in eTable 4 in the Supplement.

**Table 1.** Study-Related, Sociodemographic, Physical, Functional, and Clinical Differences in Patients With vs Without Transition to Psychosis During the Follow-up Period

Variable	Psychosis transition cohort (n = 26)	Nontransition cohort (n = 308)	Statistical analysis		*P* value (FDR)^a^	PRONIA plus 18M cohort (n = 246)	PRONIA minus 18M cohort (n = 88)	Statistical analysis		*P* value (FDR)^b^
**Samples and study variables**										
Sample site, No. of patients										
Munich	10	99	χ^2^_6_ = 6.78		.56	86	23	χ^2^_6_ = 6.53		.83
Milan	2	23				19	6			
Basel	2	32				22	12			
Cologne	4	59				50	13			
Birmingham	2	33				25	11			
Turku	6	32				24	13			
Udine	0	30				20	10			
Follow-up interval, mean (SD), d	628.3 (316.7)	727.6 (310.7)	*t*_328_ = −1.45		.38	842.7 (272.3)	390.6 (99.6)	*t*_328_ = 22.10		<.001
Sociodemographic data										
Age, mean (SD), y	23.8 (5.6)	24.8 (5.8)	*t*_332_ = −0.81		.65	24.6 (5.6)	25.0 (6.1)	*t*_332_ = −0.59		.86
Female, No (%)	10 (38.5)	160 (51.9)	χ^2^_1_ = 1.75		.38	124 (51.6)	46 (52.3)	χ^2^_1_ = 0.09		.93
Race/ethnicity, No. (%)										
White	24 (92.3)	268 (87.0)	χ^2^_4_ = 3.35		.65	214 (87.0)	78 (88.6)	χ^2^_4_ = 4.18		.83
Asian	1 (3.9)	23 (7.5)				20 (8.1)	4 (4.5)			
African	1 (3.9)	3 (1.0)				3 (1.2)	1 (1.1)			
Mixed	0	5 (1.6)				2 (0.8)	3 (3.4)			
Other	0	9 (2.9)				7 (2.8)	2 (2.3)			
BMI, mean (SD)	23.5 (4.7)	23.7 (4.6)	*t*_331_ = −0.17		.92	23.6 (4.6)	24.0 (4.6)	*t*_331_ = −0.72		.83
Edinburgh Handedness score, mean (SD)^c^	56.7 (66.6)	70.4 (50.6)	*t*_303_ = −1.28		.38	68.0 (53.1)	72.9 (49.4)	*t*_303_ = −0.70		.83
Education, mean (SD), y	13.3 (2.5)	14.3 (2.9)	*t*_331_ = −1.65		.28	14.3 (3.0)	14.1 (2.7)	*t*_331_ = 0.52		.86
Educational problems, mean (SD), y repeated	0.67 (0.88)	0.26 (0.61)	*t*_323_ = 3.20		.02	0.30 (0.64)	0.27 (0.66)	*t*_323_ = −0.34		.93
Having a partnership most of the time in the year before study inclusion, No. (%)	8 (30.8)	88 (28.6)	χ^2^_1_ = 0.57		.90	76 (30.9)	20 (22.7)	χ^2^_1_ = 2.11		.59
CHR criteria met, No. (%)										
Schizotypal personality disorder present	2 (7.7)	9 (2.9)	χ^2^_1_ = 1.71		.38	10 (4.1)	1 (1.1)	χ^2^_1_ = 1.75		.67
First-degree relatives with psychosis	3 (11.5)	25 (8.1)	χ^2^_1_ = 0.37		.83	20 (8.1)	8 (9.1)	χ^2^_1_ = 0.08		.93
30% Loss of global functioning compared with highest levels in the year before study inclusion	9 (34.6)	88 (28.6)	χ^2^_1_ = 0.43		.81	72 (29.3)	25 (28.4)	χ^2^_1_ = 0.02		.93
Criteria										
GRDP	2 (7.7)	23 (7.5)	χ^2^_1_ = 0.002		1.00	18 (7.3)	7 (8.0)	χ^2^_1_ = 0.04		.93
COGDIS	11 (42.3)	83 (26.9)	χ^2^_1_ = 2.80		.29	69 (28.0)	25 (28.4)	χ^2^_1_ = 0.004		.95
APS	18 (69.2)	88 (28.6)	χ^2^_1_ = 18.30		<.001	85 (34.6)	21 (23.9)	χ^2^_1_ = 3.42		.58
BLIPS	2 (7.7)	5 (1.6)	χ^2^_1_ = 4.30		.28	4 (1.6)	3 (3.4)	χ^2^_1_ = 1.00		.83
CHR	23 (88.5)	144 (46.8)	χ^2^_1_ = 16.68		<.001	126 (51.2)	41 (46.6)	χ^2^_1_ = 0.55		.83
**Functioning, mean (SD)**										
GF:S^d^										
Highest lifetime	7.85 (0.78)	7.96 (0.88)	*z* = −0.78		.65	7.91 (0.86)	8.06 (0.91)	*z* = −1.45		.59
Baseline	5.92 (1.72)	6.44 (1.33)	*z* = −1.32		.38	6.34 (1.38)	6.57 (1.34)	*z* = −1.22		.70
GF:R^d^										
Highest lifetime	7.92 (0.74)	8.11 (0.83)	*z* = −1.27		.38	8.15 (0.78)	7.97 (0.94)	*z* = −11.73		.59
Baseline	5.85 (1.78)	6.08 (1.66)	*z* = −0.57		.75	6.07 (1.70)	6.06 (1.56)	*z* = −0.16		.93
**High-risk symptoms, mean (SD)**										
SIPS^e^										
Positive symptoms	1.77 (0.81)	0.83 (0.80)	*t*_332_ = −5.06		<.001	0.91 (0.82)	0.89 (0.90)	*t*_332_ = 0.25		.93
Negative symptoms	1.90 (1.57)	1.55 (1.02)	*t*_332_ = −0.54		.75	1.58 (1.04)	1.48 (0.99)	*t*_332_ = 1.20		.70
Disorganized symptoms	1.07 (1.15)	0.66 (0.60)	*t*_332_ = −1.84		.26	0.69 (0.62)	0.60 (0.55)	*t*_332_ = 1.54		.59
General psychopathology	2.29 (1.19)	1.86 (0.95)	*t*_332_ = −1.65		.28	1.92 (0.94)	1.71 (0.95)	*t*_332_ = 2.24		.47
**History of *DSM-IV* comorbid disorders at study inclusion**										
Any affective, substance, anxiety, or eating disorders at study inclusion, diagnosis, No. (%)										
None	5 (19.2)	135 (44.1)	χ^2^_3_ = 8.20		.21	102 (41.8)	39 (44.8)	χ^2^_3_ = 1.12		.93
1	13 (50.0)	93 (30.4)				78 (32.0)	27 (31.0)			
2	6 (23.1)	42 (13.7)				38 (15.6)	10 (11.5)			
≥3	2 (7.7)	36 (11.8)				26 (10.7)	11 (12.6)			
Major depressive disorder, No. (%)										
No	9 (34.6)	58 (19.0)	χ^2^_1_ = 3.65		.26	47 (19.2)	20 (23.3)	χ^2^_1_ = 0.58		.83
Yes	17 (65.4)	248 (81.0)				198 (80.8)	67 (77.0)			
Affective disorder, No. (%)^f^										
None	12 (46.2)	200 (65.4)	χ^2^_2_ = 5.45		.26	154 (62.9)	59 (67.8)	χ^2^_2_ = 1.55		.83
1	14 (53.8)	98 (32.0)				86 (35.1)	25 (28.7)			
2	0	8 (2.6)				5 (2.0)	3 (3.4)			
Substance use disorder, No. (%)										
No	25 (96.2)	302 (98.7)	χ^2^_2_ = 1.64		>.99	241 (98.8)	86 (98.9)	χ^2^_1_ = 0.003		.95
Yes	1 (3.8)	3 (1.0)				3 (1.2)	1 (1.1)			
Anxiety disorders, No. (%)										
None	17 (65.4)	212 (69.3)	χ^2^_3_ = 1.35		.86	172 (70.2)	58 (66.7)	χ^2^_3_ = 1.92		.86
1	7 (26.9)	59 (19.3)				45 (18.4)	21 (24.1)			
2	1 (3.8)	25 (8.2)				21 (8.6)	5 (5.7)			
≥3	1 (3.8)	10 (3.3)				7 (2.9)	3 (3.4)			
Eating disorders, No. (%)										
None	24 (92.3)	290 (94.8)	χ^2^_2_ = 0.90		.83	230 (93.9)	84 (96.6)	χ^2^_2_ = 5.55		.58
1	2 (7.7)	13 (4.2)				14 (5.7)	1 (1.1)			
2	0	3 (1.0)				1 (0.4)	2 (2.3)			
Treatments, No. (%)										
Antipsychotics	10 (38.5)	53 (17.2)	χ^2^_1_ = 7.08		.09	40 (20.2)	14 (15.9)	χ^2^_1_ = 0.76		.83
Antidepressants	13 (50.0)	176 (57.1)	χ^2^_1_ = 0.50		.74	140 (57.6)	48 (54.5)	χ^2^_1_ = 0.25		.86
Inpatient	15 (60.0)	144 (46.8)	χ^2^_1_ = 2.10		.38	122 (50.2)	36 (40.9)	χ^2^_1_ = 2.24		.59
Psychotherapy	14 (56.0)	229 (74.4)	χ^2^_1_ = 5.09		.21	179 (73.7)	62 (70.5)	χ^2^_1_ = 0.34		.86

Abbreviations: APS, attenuated positive symptom; BLIPS, brief limited intermittent psychotic symptoms; BMI, body mass index (calculated as weight in kilograms divided by square of height in meters); CHR, clinical high-risk; COGDIS, Schizophrenia Proneness Instrument: Cognitive Disturbances criteria; FDR, false discovery rate; GF:R Global Functioning Scale: Role; GF:S, Global Functioning Scale: Social; GRDP, genetic risk and deterioration psychosis risk syndrome; PRONIA plus 18M, Personalised Prognostic Tools for Early Psychosis Management (PRONIA) with follow-up of at least 18 months; PRONIA minus 18M, PRONIA with follow-up of less than 18 months; SIPS, Structured Interview for Psychosis–Risk Syndromes.

^a^ Calculated as psychosis transition vs nontransition groups.

^b^ Calculated as PRONIA plus 18M vs PRONIA minus 18M samples.

^c^ Scores range from −100 to 100, with higher scores indicating more pronounced right-handedness.

^d^ Scores range from 0 to 10, with higher scores indicating better social functioning.

^e^ Scores range from 0 to 6, with higher scores indicating more severe symptoms.

^f^ Excludes major depressive disorder.

### Prognostic Modeling Strategy

Data were analyzed from January 1, 2019, to March 31, 2020. Using the machine learning software NeuroMiner, version 1.05 (GitHub [https://github.com/neurominer-git/NeuroMiner-1]), we constructed and tested unimodal, multimodal, and clinically scalable sequential risk calculators for transition prediction in the PRONIA plus 18M cohort using leave-one-site-out cross-validation (LOSOCV)^21,41^ (eMethods and eFigures 2-4 in the Supplement). We evaluated the risk calculators using baseline and longitudinal data and validated their specificity in the PRONIA minus 18M sample, which did not include cases with psychosis transition but provided all data modalities. In addition, 3 external data sets consisting of cases with psychosis transition and nontransition were available to test selected models.^35,36,37^

**Figure 1.**
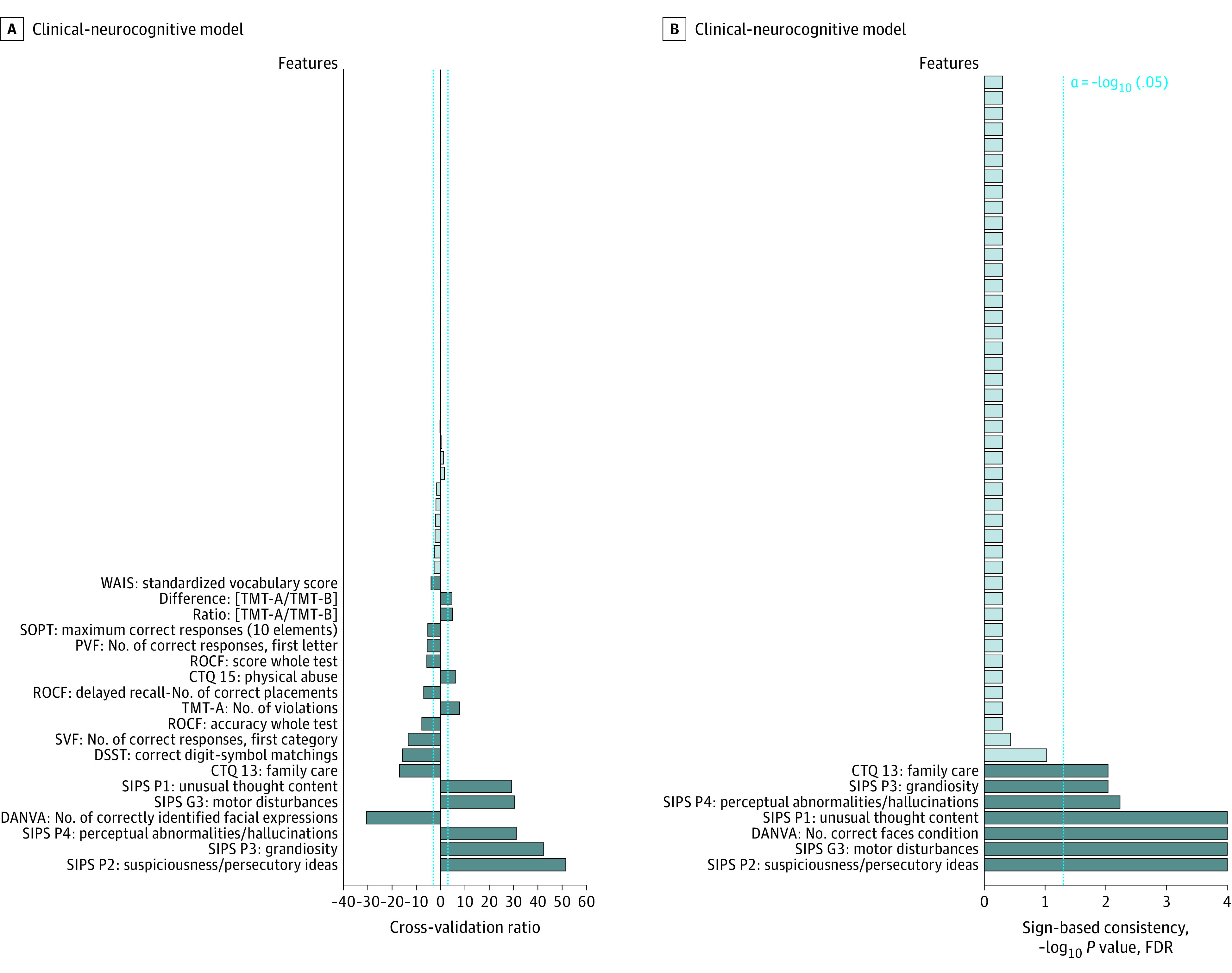
Predictive Signatures Underlying the Clinical-Neurocognitive Models The reliability of predictive pattern elements was evaluated using cross-validation ratio mapping (A). In addition, the significance of predictive features used by the clinical-neurocognitive model was assessed by means of sign-based consistency mapping (B). Both visualization methods are detailed in the eMethods in the Supplement. CTQ indicates Childhood Trauma Questionnaire; DANVA, Diagnostic Analysis of Non-Verbal Accuracy; DSST, Digit-Symbol Substitution Test; FDR, false discovery rate; PVF, Phonetic Verbal Fluency; ROCF, Rey-Osterreith Figure; SIPS, Structured Interview for Psychosis-Risk Syndromes; SOPT, Self-Ordered Pointing Task; SVF, Semantic Verbal Fluency; TMT, Trail Making Test; and WAIS, Wechsler Adult Intelligence Scale.

**Figure 2.**
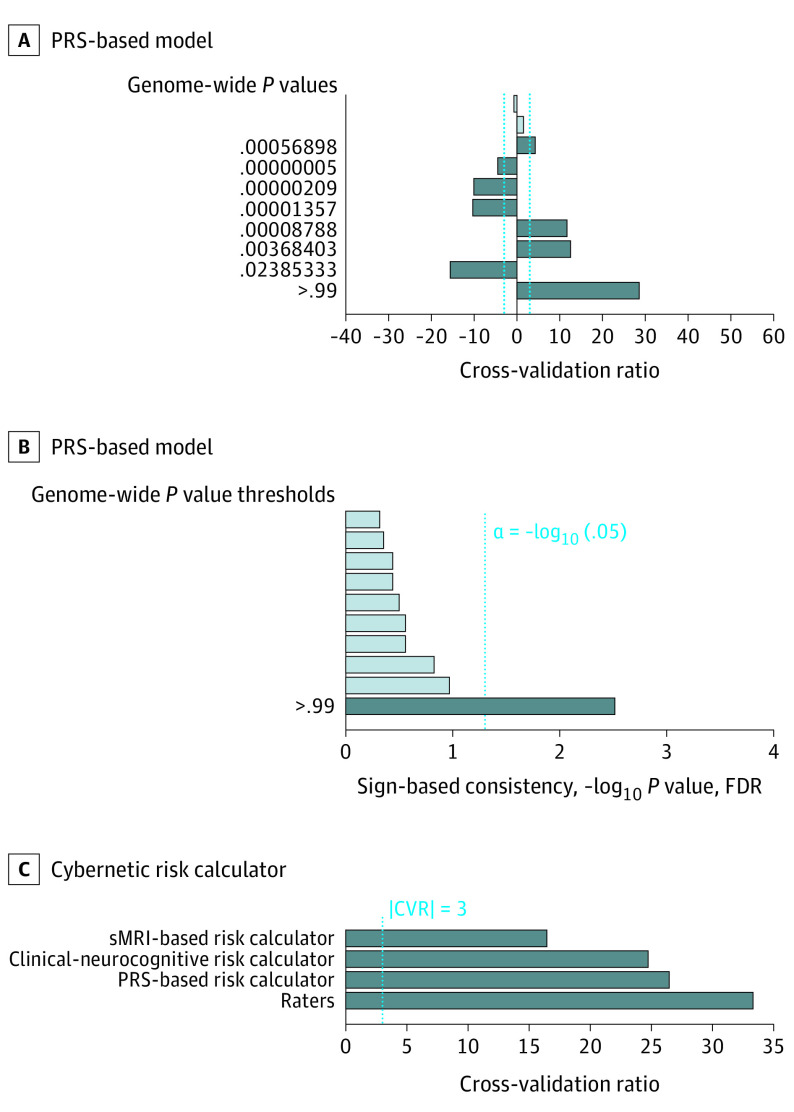
Predictive Signatures Underlying the Polygenic Risk Score (PRS)–Based and Cybernetic Risk Calculator Models The reliability of predictive pattern elements was evaluated using cross-validation ratio (CVR) mapping (A). In addition, the significance of predictive features used by the PRS-based model was assessed by means of sign-based consistency mapping (B) (as described in the eMethods in the Supplement). The cybernetic model combines all algorithmic and human components (C). FDR indicates false discovery rate; and sMRI, structural magnetic resonance imaging.

**Figure 3.**
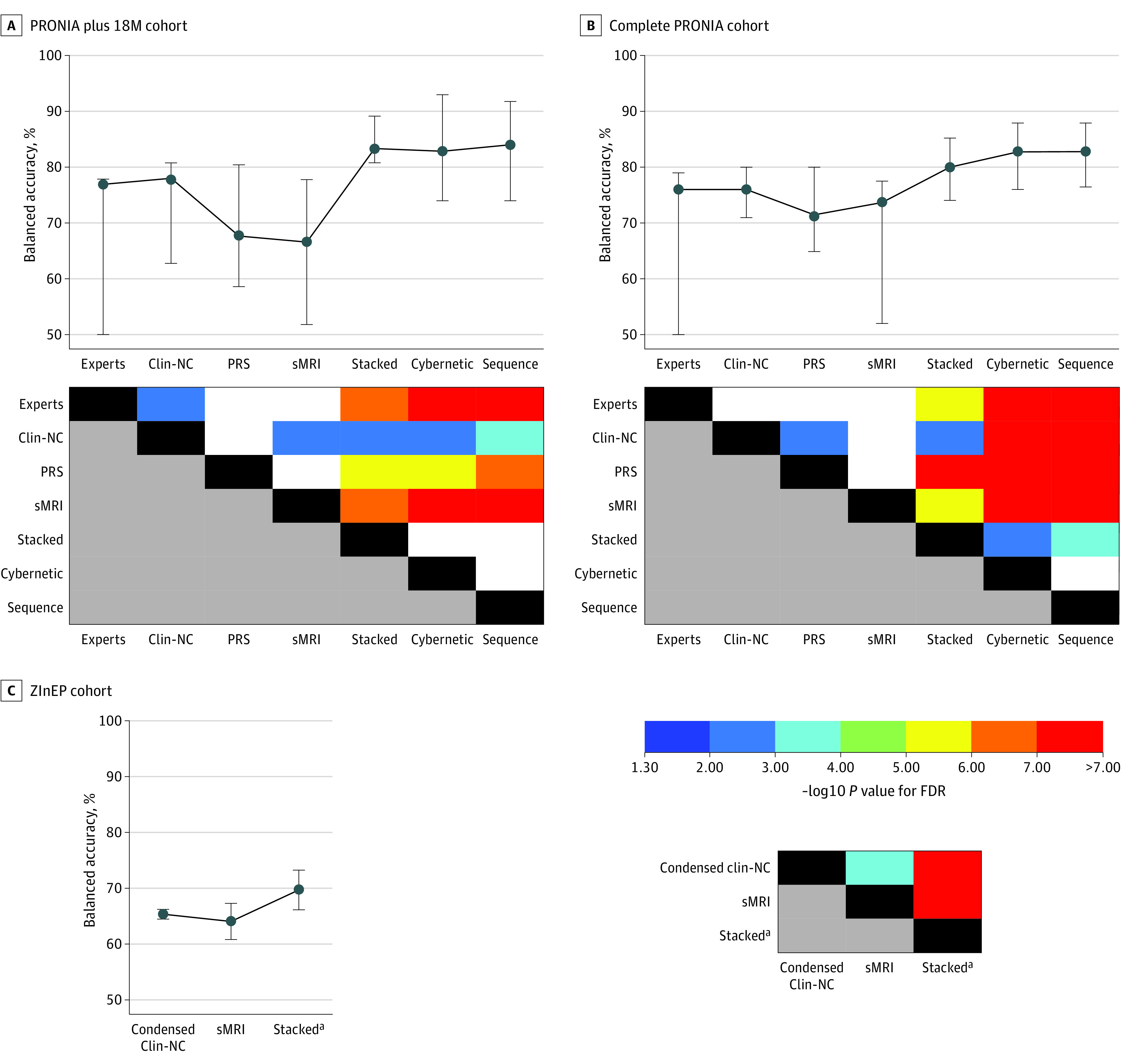
Statistical Comparison of Prognostic Models Cohorts include patients with follow-up of 18 months or longer (PRONIA plus 18M), the complete PRONIA cohort, and the Zurich Early Recognition Program (ZInEP). Data points indicate median. The Quade test^51^ was used to compare the models’ median balanced accuracy (BAC) computed across the cross-validation cycle (CV_2_) test data partitions. The BAC measures obtained for the ZInEP cohort (C) were produced by applying the condensed clinical-neurocognitive (Clin-NC), structural magnetic resonance imaging (sMRI)–based, and respective stacked risk calculators of the complete PRONIA sample (B) to this external sample (eFigure 2 in the Supplement). Post hoc comparisons were performed using the t distribution approximation described by Heckert and Filliben.^52^ *P* values were corrected for multiple comparisons using the false discovery rate (FDR). The upper graphs represent the median BAC for each risk calculator in analyses A, B, and C along with the lower and upper quartiles of the BAC distributions (whiskers of the error bars). The lower figures show the logarithmized, FDR-corrected *P* matrix for the pairwise post hoc classifier comparisons. For an in-depth analysis of the prognostic sequence included in the risk classifier comparison, see eFigures 14 and 15 in the Supplement. The cybernetic risk calculator analyzed the combined predictions of raters, Clin-NC, polygenic risk score (PRS)–based, and sMRI-based risk calculators; the stacked risk calculator, the combined predictions of Clin-NC, PRS-based, and sMRI-based risk calculators. ^a^Indicates risk calculator encompassing the condensed Clin-NCs and sMRI-based models and specifically trained to externally validate the effect of stacking on prognostic performance in the ZInEP cohort.

First, unimodal risk calculators were trained with literature-based baseline predictors of psychosis transition,^2,6,16^ including prodromal symptoms,^39,42^ functioning,^43,44^ childhood adversity,^45,46^ and neurocognitive measures^6,47^ in the clinical-neurocognitive domain (eTable 5 in the Supplement); PRS for schizophrenia^48^ in the genetic domain^16^; and gray matter volume maps in the structural magnetic resonance imaging (sMRI) domain^8,9^ (eTable 6 in the Supplement). In addition, we evaluated our raters’ predictions, which, at the conclusion of baseline assessments, were provided as yes or no replies to the question, “Do you think the patient will likely transition to psychosis?” Then we assessed whether combining unimodal algorithms using stacked generalization^49^ improved prognostic accuracy (eFigures 2 and 3 in the Supplement and Table 2).^13^ Following the concept of expert-based machine learning,^50^ we integrated our raters’ estimates as additional predictors to produce a cybernetic model^33^ (eFigure 2 in the Supplement). Models’ predictive signatures were visualized in Figures 1 and 2 and eFigure 5 in the Supplement using measures of pattern element stability (cross-validation ratio) and pattern element significance (sign-based consistency; eMethods in the Supplement). In addition, the prognostic models were assessed using random-label permutations (Table 2). Raters’ and models’ performances were compared statistically at the omnibus level using the Quade test,^51^ an extension of the nonparametric Wilcoxon signed rank test, followed by post hoc pairwise mean differences tests using the *t* distribution.^52^ Statistical significance was determined at α = .05. Obtained *P* values were 2-sided; *P* values computed in the pairwise classifier comparisons were corrected using the false discovery rate (FDR) (Figure 3). Classifiers were visually compared in eFigure 6 in the Supplement).^51,52^

**Table 2.** Prediction Performance of Clinical Raters; Unimodal, Stacked, and Cybernetic Risk Calculators; and Prognostic Workflows^a^

Model by cohort	No. of findings				Sensitivity, %	Specificity, %	BAC, %	PPV	NPV	PSI	Positive LR	AUC	*P* value for FDR
	True positive	True negative	False positive	False negative									
**Rater-based estimates **													
PRONIA plus 18M	16	185	33	10	61.5	84.9	73.2	32.7	94.9	27.5	4.1	0.73	NA
PRONIA minus 18M	NA	75	11	NA	NA	87.2	NA	NA	NA	NA	NA	NA	NA
Complete PRONIA	16	260	44	10	61.5	85.5	73.5	26.7	96.3	23.0	4.3	0.74	NA
**Clin-NC risk calculator **													
PRONIA plus 18M	22	147	73	4	84.6	66.8	75.7	23.2	97.4	20.5	2.6	0.83	NA
PRONIA minus 18M^b^	NA	58	30	NA	NA	65.9	NA	NA	NA	NA	NA	NA	NA
Complete PRONIA	21	201	107	5	80.8	65.3	73.0	16.4	97.6	14.0	2.3	0.79	<.001
**Condensed Clin-CN risk calculator using 7 significant predictors**													
PRONIA minus 18M^b^	NA	58	30	NA	NA	65.9	NA	NA	NA	NA	NA	NA	NA
ZInEP^b^	14	74	56	2	87.5	43.1	65.3	15.9	96.6	12.5	1.5	0.67	NA
BEARS-Kid^b^	10	287	162	3	76.9	63.9	70.4	5.8	99.0	4.8	2.1	0.68	NA
**PRS-based risk calculator **													
PRONIA plus 18M	19	113	88	6	76.0	56.2	66.1	17.8	95.0	12.7	1.7	0.74	NA
PRONIA minus 18M^b^	NA	35	37	NA	NA	48.6	NA	NA	NA	NA	NA	NA	NA
Complete PRONIA	22	157	116	3	88.0	57.5	72.8	15.9	98.1	14.1	2.1	0.74	<.001
**sMRI-based risk calculator **													
PRONIA plus 18M	22	116	101	3	88.0	53.5	70.7	17.9	97.5	15.4	1.9	0.70	NA
PRONIA minus 18M^b^	NA	40	48	NA	NA	45.5	NA	NA	NA	NA	NA	NA	NA
Complete PRONIA	22	164	137	3	88.0	54.5	71.2	13.8	98.2	12.0	1.9	0.73	<.001
FePsy^b^	12	13	8	4	75.0	61.9	68.5	60.0	76.5	36.5	2.0	0.71	NA
ZInEP^b^	12	79	51	4	75.0	60.8	67.9	19.0	95.2	14.2	1.9	0.71	NA
**Stacked risk calculator analyzing the predictions of the Clin-NC and sMRI classifiers**													
PRONIA minus 18M^b^	NA	70	18	NA	NA	79.5	NA	NA	NA	NA	NA	NA	NA
Complete PRONIA	21	265	43	5	80.8	86.0	83.4	32.8	98.1	31.0	5.8	0.89	NA
ZInEP^b^	12	88	42	4	75.0	67.7	71.3	22.2	95.7	17.9	2.3	0.74	NA
**Stacked Clin-NC, PRS, and sMRI risk calculator **													
PRONIA plus 18M	21	187	33	5	80.8	85.0	82.9	38.9	97.4	36.3	5.4	0.88	NA
PRONIA minus 18M^b^	NA	71	17	NA	NA	80.7	NA	NA	NA	NA	NA	NA	NA
Complete PRONIA	18	263	45	8	69.2	85.4	77.3	28.6	97.0	25.6	4.7	0.86	<.001
**Cybernetic risk calculator including raters, Clin-NC, PRS, and sMRI**													
PRONIA plus 18M	22	190	30	4	84.6	86.4	85.5	42.3	97.9	40.2	6.2	0.90	NA
PRONIA minus 18M^b^	NA	73	15	NA	NA	83.0	NA	NA	NA	NA	NA	NA	NA
Complete PRONIA	21	266	42	5	80.8	86.4	83.6	33.3	98.2	31.5	5.9	0.90	<.001
**Optimal prognostic workflow**^c^													
PRONIA plus 18M	22	192	28	4	84.6	87.3	85.9	44.0	98.0	42.0	6.6	0.90	NA
PRONIA minus 18M^b^	NA	74	14	NA	NA	84.1	NA	NA	NA	NA	NA	NA	NA
Complete PRONIA	21	267	41	5	80.8	86.7	83.7	33.9	98.2	32.0	6.1	0.89	<.001
**Prognostic workflows optimized for clinical scalability**													
PRONIA minus 18M cohort^b^^,^^c^	NA	74	14	NA	NA	84.1	NA	NA	NA	NA	NA	NA	NA
Workflow optimized for light examination sparsity^d^													
PRONIA plus 18M	21	189	31	5	80.8	85.9	83.3	40.4	97.4	37.8	5.7	0.87	NA
PRONIA minus 18M^b^	NA	74	14	NA	NA	84.1	NA	NA	NA	NA	NA	NA	NA
Complete PRONIA	19	266	42	7	73.1	86.4	79.7	31.1	97.4	28.6	5.4	0.85	<.001
PRONIA minus 18M cohort^b^^,^^d^	NA	75	13	NA	NA	85.2	NA	NA	NA	NA	NA	NA	NA
Workflow optimized for strong examination sparsity^e^													
PRONIA plus 18M	22	164	56	4	84.6	74.5	79.6	28.2	97.6	25.8	3.3	0.84	NA
PRONIA minus 18M^b^	NA	67	21	NA	NA	76.1	NA	NA	NA	NA	NA	NA	NA
Complete PRONIA	21	228	80	5	80.8	74.0	77.4	20.8	97.9	18.6	3.1	0.81	<.001
PRONIA minus 18M cohort^b^^,^^e^	NA	63	25	NA	NA	71.6	NA	NA	NA	NA	NA	NA	NA

Abbreviations: AUC, area under the curve; BAC, balanced accuracy; BEARS-Kid, Bi-national Evaluation of At-Risk Symptoms in Children and Adolescents; Clin-NC, clinical-neurocognitive; FDR, false discovery rate; LR, likelihood ratio; NA, not applicable; NPV, negative predictive value; PPV positive predictive value; PRONIA plus 18M, Personalised Prognostic Tools for Early Psychosis Management (PRONIA) with follow-up of at least 18 months; PRONIA minus 18M, PRONIA with follow-up of less than 18 months; PRS, polygenic risk score; PSI, Prognostic Summary Index; sMRI, structural magnetic resonance imaging; ZInEP, Zurich Early Recognition Program.

^a^ Risk calculators were first trained and cross-validated in the PRONIA plus 18M cohort and then validated in the PRONIA minus 18M sample. To estimate the models’ significance, they were retrained and cross-validated using the complete PRONIA cohort. Model significance was computed for each risk calculator using 1000 label permutations in the complete PRONIA cohort (eMethods in the Supplement), and *P* values were corrected for multiple comparisons using the FDR. External validation was conducted for the condensed Clin-NC model, the sMRI-based risk calculator, and the stacked model. These models were retrained in the complete PRONIA cohort before external validation. In addition, prognostic workflows that included the condensed Clin-NC model were validated in the PRONIA minus 18M sample. eFigure 2 and the eMethods in the Supplement give a detailed description of the entire analysis process leading from unimodal to workflow models.

^b^ Because variable extraction for the condensed Clin-NC model was performed in the PRONIA plus 18M sample, we report its performance and the respective metrics of its dependent stacked model only for the PRONIA minus 18M, the ZInEP, and BEARS-Kid samples.

^c^ Γ = 0.0 (case propagation cutoffs: 25.0% and 100%). Sequence is Clin-NC, Clin-NC plus raters, Clin-NC plus PRS, and Clin-NC plus sMRI.

^d^ Γ = 0.5 (case propagation cutoffs: 37.5% and 100%). Sequence is Clin-NC, Clin-NC plus PRS, Clin-NC plus raters, and Clin-NC plus sMRI.

^e^ Γ = 1.0 (case propagation cutoffs: 37.5% and 75.0%). Sequence is Clin-NC, Clin-NC plus PRS, Clin-NC plus raters, and Clin-NC plus sMRI.

### In-Depth Model Evaluation

We tested differences between the prognostic assignment groups and the matched healthy control group to determine whether the models’ predictive patterns represented a deviation from normality (eFigures 7-9 in the Supplement). Potential model confounders and moderators were systematically tested, including image quality (eFigure 10 in the Supplement), treatments (eTable 7 in the Supplement), follow-up frequency and duration (eFigure 11 and eTable 8 in the Supplement), site-related effects (eTables 9-11 in the Supplement), baseline study group membership (eTables 12 and 13 in the Supplement), and, specifically, the inclusion of patients with ROD (eTable 14 and eFigure 12 in the Supplement). Using the patients’ longitudinal data, we evaluated whether model predictions were not specific for the binary transition vs nontransition outcome, but we also separated transitions from nonremitting/de novo CHR symptom courses (P-CHR) and asymptomatic/nonpersisting trajectories (NP-CHR) (eTable 15 in the Supplement). To further explore a prognostic generalization effect,^13^ we used unsupervised machine learning (eMethods in the Supplement) to construct fine-grained CHR syndrome trajectories (eFigure 13A in the Supplement) and used linear mixed-effects modeling to compare trajectories between predicted and observed outcome groups (eFigure 13B [part 1] in the Supplement). Finally, we investigated whether assignments generalized to the prediction of nonpsychotic outcomes (eTable 16 in the Supplement).

### Optimization for Clinical Scalability

To facilitate clinical implementation, we developed a sequential prediction method that optimizes the ordering and number of data modalities as well as the prognostic uncertainty thresholds to decide whether a patient needs further testing (eTable 17 and eMethods in the Supplement). We analyzed the identified optimal prognostic workflow (eFigure 14 in the Supplement) and tested whether it achieved similar performance as the fully stacked models at lower diagnostic burden for the patients. To further enhance clinical scalability, we condensed the clinical-neurocognitive model, which was the workflow’s entry point, from 141 to 7 (5.0%) variables (Figure 2) using sign-based consistency mapping (eMethods in the Supplement).^53^ We tested the condensed model and the respective workflow’s specificity in the PRONIA minus 18M sample (Table 2). Finally, we explored whether the prognostic sequence could be further trimmed using diagnostic parsimony regularization (eMethods in the Supplement and Table 2). Nonregularized and regularized workflows including the full or condensed clinical-neurocognitive models were compared in eFigure 15 in the Supplement.

### External Validation Experiments

We validated the condensed clinical-neurocognitive model in 2 external cohorts: 146 patients with CHR (aged 15-35 years; 16 [11.0%] transitions) provided by the Zurich Early Recognition Program (ZInEP) (eTable 18 in the Supplement)^36^ and 462 patients with diverse mental conditions (aged 8-17 years; 13 [2.8%] transitions) drawn from the Bi-national Evaluation of At-Risk Symptoms in Children and Adolescents study (eTable 19 in the Supplement).^37^ Second, we validated the sMRI-based model in ZInEP and in 37 patients with CHR (16 [43.2%] transitions) from the Früherkennung von Psychosen study (Table 2).^35,54^ To validate the increased performance of multimodal risk calculators, we trained a stacked model using the condensed clinical-neurocognitive and sMRI-based models, tested it in the ZInEP data (Table 2), and used the Quade test^51^ to compare the 2 unimodal prediction models with the stacked classifier (Figure 3). Finally, we made our models available in the NeuroMiner Model Library (http://www.proniapredictors.eu) to facilitate their independent external validation.

## Results

### Group-Level Differences

A total of 668 patients and controls were included in the analysis (mean [SD] age, 25.1 [5.8] years; 354 [53.0%] female and 314 [47.0%] male). Patients in the PRONIA plus 18M and PRONIA minus 18M groups were followed up for a mean (SD) of 842.7 (272.3) and 390.6 (99.6) days, respectively (Table 1). They did not differ in any examined variable (Table 1). Psychosis transition occurred after a mean (SD) of 246.9 (244.5) days in 26 cases and developed into schizophrenia in 8 (30.8%) (eTable 4 in the Supplement). Follow-up durations differed between sites but not time to psychosis transition (eTable 9 in the Supplement). Compared with nontransition, individuals with psychosis transition had more repeated school years (mean [SD], 0.67 [0.88] vs 0.26 [0.61] years) and more prevalent attenuated positive symptoms (APSs) at baseline (18 of 26 [69.2%] vs 88 of 308 [28.6%]) (Table 1). Of 167 patients with CHR, 23 (13.8%) developed psychosis, whereas 53 (31.7%) had remitted from CHR criteria at the 9-month visit. Major depression affected 103 patients with CHR syndromes (61.7%) but was not differentially associated with psychosis transition (Table 1). Nonremitting mood and anxiety disorders were present during follow-up in cases with psychosis transition and nontransition (eTable 16 in the Supplement). Compared with healthy controls, ROD was associated with low but significantly elevated CHR symptom scores (eg, mean [SD] Structured Interview for Psychosis–Risk Syndromes positive symptoms, 0.43 [0.44] vs 0.10 [0.21]; *P* < .001; mean [SD] Schizophrenia Proneness Instrument: Cognitive Disturbances symptoms, 0.24 [0.32] vs 0.02 [0.08]; *P* < .001) (eTable 3 in the Supplement). Functional-cognitive and interpersonal abnormalities were comparable between ROD and CHR groups. Of 167 patients with ROD, 32 (19.2%) developed psychosis-related outcomes, including CHR states in 29 (17.4%) and psychosis transitions in 3 (1.8%).

### Machine Learning Analyses

The full clinical-neurocognitive model predicted psychosis transition with a balanced accuracy (BAC-LOSOCV) of 75.7% (sensitivity, 84.6%; specificity, 66.8%; *P* < .001) (Table 2). Significant predictors as determined by sign-based consistency mapping (*z*>3.28; *P* < .05 for FDR) were APS and motor disturbances, a nonsupportive family environment during childhood, and reduced facial emotion recognition (Figure 1B). Compared with healthy controls, those assigned to psychosis transition had elevated abnormality scores in these variables, whereas those with nontransition assignments showed an abnormality pattern focused on unusual thought content, suspiciousness, perceptual abnormalities, and childhood adversity, with higher visual working memory and semantic verbal fluency performance (*P* < .05 for FDR) (eFigure 7 in the Supplement).

The PRS-based model achieved a BAC-LOSOCV of 66.1% (sensitivity, 76.0%; specificity, 56.2%; *P* < .001). Among the 10 tested genome-wide significance thresholds, only *P* = 1.0 reached significance (Figure 2). Compared with healthy controls, those with psychosis transition assignments had elevated PRS across all whole-genome *P* thresholds, whereas those with nontransition assignments expressed reduced PRS at *P* ≥ 5.7 × 10^−4^ (eFigure 8A in the Supplement). Patients with observed nontransition and those with ROD did not show reduced PRS (eFigure 8B-C in the Supplement).

The sMRI-based model attained a BAC-LOSOCV of 70.7% (sensitivity, 88.0%; specificity, 53.5%; *P* < .001). At a stability threshold (cross-validation ratio) of at least 3, the brain pattern predicting psychosis transition involved reduced gray matter volume in the superior temporal, supramarginal, angular, orbitofrontal, inferior frontal, dorsomedial prefrontal, and occipital cortices. The predictive pattern also included areas of increased gray matter volume covering the dorsolateral prefrontal, precuneal, insular, hippocampal, and cerebellar brain regions (eFigure 5 in the Supplement). This brain signature differentiated psychosis transition-assigned patients from healthy controls, whereas nontransition-assigned patients showed a partial pattern inversion with increased temporo-occipital gray matter volume compared with healthy controls (see threshold-free cluster enhancement statistics thresholded at *P* < .05 for FDR) (eFigure 9 in the Supplement).

Clinical raters achieved a BAC of 73.2% (sensitivity, 61.5%; specificity, 84.9%), which was independent of the length of their early recognition experience (mean [SD] length for correct predictions: 31.8 [46.8] months; mean [SD] length for wrong predictions, 29.4 [38.1] months; unpaired 2-tailed *t*_326_ = 0.35; *P* = .72). The stacked model combining unimodal algorithms produced a BAC-LOSOCV of 82.9% (sensitivity, 80.8%; specificity, 85.0%). Integration of raters’ prognoses into the stacked model increased BAC-LOSOCV to 85.5% (sensitivity, 84.6%; specificity, 86.4%), and they were the cybernetic model’s most relevant predictor (Figure 2C).

### Baseline Moderators of Prediction Performance

Image quality (eFigure 10 in the Supplement), baseline treatments or previous hospitalizations (eTable 7 in the Supplement), follow-up duration and frequency (eTable 8 and eFigure 11 in the Supplement), and site effects (eTable 10 in the Supplement) did not influence model performance. Study group (CHR vs ROD) could be classified with a BAC-LOSOCV of 82.3% (sensitivity, 73.7%; specificity, 91.0%) using clinical-neurocognitive data and with a BAC of 55.7% (sensitivity, 49.7%; specificity, 61.7%) using PRS (eTable 12 in the Supplement). These diagnostic classifiers explained 49.7% and 18.3%, respectively, of the variance of the respective prognostic counterparts (both *P* < .001 for FDR) (eTable 13 in the Supplement). Raters’ prognoses were also significantly informed by baseline study group (BAC, 62.7%; sensitivity, 30.9%; specificity, 94.5%) (eTable 12 in the Supplement). The removal of the patients with ROD from the training samples or their substitution with healthy controls significantly reduced the balanced accuracy of all risk calculators by −2.8% to −11.7% in the CHR group (eTable 14 and eFigure 12 in the Supplement).

### Prognostic Generalization

Prognostic assignments also delineated psychosis transition and P-CHR and NP-CHR courses irrespective of model type (eTable 15 in the Supplement). The separability of P-CHR from NP-CHR courses was lower and only significant for clinical-neurocognitive models and raters. The nonnegative matrix factorization and linear mixed-model analysis showed that CHR syndrome trajectories were stratified by the predictions of the clinical-neurocognitive classifier (factor F1 paranoid-perceptual disturbances, *F*_1,832_ = 136.35 [*P* < .001 for FDR]; factor F2 disturbances of volition and affect, *F*_1,832_ = 12.76 [*P* = .001 for FDR]; factor F3 functional disturbances, *F*_1,832_ = 24.34 [*P* < .001 for FDR]; factor F4 cognitive disturbances, *F*_1,832_ = 44.05 [*P* = .007 for FDR]) as well as by raters' outcome estimates (factor F1 paranoid-perceptual disturbances, *F*_1,825_ = 30.64 [*P* < .001 for FDR]; factor F2 disturbances of volition and affect, *F*_1,825_ = 6.15 [*P* = .03 for FDR]; factor F3 functional disturbances, *F*_1,825_ = 5.80 [*P* < .03 for FDR]; factor F4 cognitive disturbances, *F*_1,825_ = 9.00 [*P* = .007 for FDR]) (eFigure 13 in the Supplement). Nonpsychotic disease courses were not associated with prognostic assignments (eTable 16 in the Supplement).

### Clinical Scalability and External Validation of Predictive Models

We identified a prognostic sequence that produced a BAC-LOSOCV of 85.9% (sensitivity, 84.6%; specificity, 87.3%) (Table 2) and started with the clinical-neurocognitive model, added raters, and finally integrated PRS- and sMRI-based models (eFigure 14 in the Supplement). Across this sequence, the positive likelihood ratio increased to 6.6, whereas the population requiring all prognostic assessments decreased to 41.1%. Regularization for diagnostic parsimony further reduced this population to 23.2% (regularization strength Γ = 0.5) and 0 (regularization strength Γ = 1.0) (PRONIA plus 18M sample, left panel of eFigure 15B in the Supplement), with the latter parsimony level significantly reducing BAC by −6.4% (PRONIA plus 18M sample, left panel of eFigure 15C, part 2 in the Supplement). Highly similar findings were obtained when analyzing the complete PRONIA cohort (right panels in eFigure 15B and C in the Supplement).

Further clinical scalability experiments showed that the condensed clinical-neurocognitive model matched the full model in correctly predicting PRONIA minus 18M cases with nontransition (specificity, 65.9%). Furthermore, its performance in the external ZInEP-CHR cohort (BAC, 65.3%; sensitivity, 87.5%; specificity, 43.1%) and the Bi-national Evaluation of At-Risk Symptoms in Children and Adolescents sample (BAC, 70.4%; sensitivity, 76.9%; specificity, 63.9%) was similar to the full model in the PRONIA-CHR sample (BAC, 63.3%; sensitivity, 87.0%; specificity, 39.6%) (eTable 14 in the Supplement) and the complete PRONIA cohort (BAC, 72.8%; sensitivity, 88.0%; specificity, 57.5%) (Table 2). Replacing the full model with its condensed counterpart in the nonregularized or regularized (Γ = 0.5) workflows did not increase the false-positive rate in the PRONIA minus 18M sample or the complete PRONIA cohort (eFigure 14C in the Supplement).

The validation of the unimodal sMRI-based workflow component in the ZInEP (BAC, 67.9%; sensitivity, 75.0%; specificity, 60.8%) and Früherkennung von Psychosen (BAC, 68.5%; sensitivity, 75.0%; specificity, 61.9%) samples approximated the PRONIA-CHR results (BAC, 70.8%; sensitivity, 86.4%; specificity, 55.3%) (eTable 14 in the Supplement). Finally, the stacked risk calculator composed of the condensed clinical-neurocognitive and sMRI-based models significantly outperformed these models in the ZInEP data (BAC, 71.3%; sensitivity, 75.0%; specificity, 67.7%) (Figure 3 and Table 2).

### Comparisons of Risk Calculators and Clinical Raters

All risk calculators were significant in the permutation analysis (mean [SD] BAC, 77.3 [4.8]; mean [SD] sensitivity, 80.2% [6.5%]; mean [SD] specificity, 74.5% [13.8%]; *P* < .001 for FDR for all models) Table 2), but differences in BAC emerged (Figure 3). Multimodal risk calculators outperformed all unimodal counterparts (*t* range, 3.14-6.20; *P* < 3.19 × 10^−8^ to *P* = .002 for FDR), whereas the nonregularized prognostic sequence did not differ from the cybernetic model (mean [SD] BAC for nonregularized sequence, 83.7% [9.6%]; mean [SD] BAC for cybernetic model, 83.4% [9.6%]; *t* = 0.21; *P* = .44 for FDR). In addition, the stacked model (mean [SD] BAC, 79.7% [7.9%]) was outperformed by both the cybernetic model (mean [SD] BAC, 81.5% [9.6%]; *t* = 3.18; *P* = .001 for FDR) and the nonregularized sequential model (mean [SD] BAC, 82.0% [9.6%]; *t* = 3.82; *P* < .001 for FDR) in the complete PRONIA sample. Raters were comparable to unimodal risk calculators (mean [SD] BAC for raters, 68.2% [12.6%]; mean [SD] BAC range for unimodal predictors, 67.5% [16.4%] to 74.8% [6.8%]; *t* range, 0.002-1.56; *P* = .50 to *P* = .07 for FDR) but were outperformed by multimodal prediction algorithms in terms of higher BAC and reduced prognostic variability (stacked model vs raters, *t* = 4.64 [*P* < .001 for FDR]; cybernetic model vs raters, *t* = 7.82 [*P* < .001 for FDR]; sequence model vs raters, *t* = 8.46 [*P* < .001 for FDR]). Finally, the nonregularized sequential model reduced raters’ false-negative rate from 38.5% to 15.4% (PRONIA plus 18M sample) or 19.2% (complete PRONIA cohort) of cases.

## Discussion

Using a thorough model discovery and validation approach,^21^ our study demonstrated geographic transportability of expert-based clinical and biological psychosis transition prediction approaches across a transdiagnostic, multinational risk population. We found that combined risk calculators outperformed all unimodal counterparts and clinical raters in terms of prognostic accuracy and cross-site stability. Importantly, our study revealed that the increased diagnostic burden arising from data fusion could be mitigated through optimized sequential testing that arranges clinicians and risk calculators into clinically scalable prognostic workflows. Based on this form of deferral learning,^55^ we showed that the complete assessment battery is only needed in 23.2% of the initial population (eFigure 15B in the Supplement). This subgroup was enriched for patients who received a prediction of psychosis transition in the initial clinical-neurocognitive examination, suggesting that biological markers of psychosis transition are useful for delineating true-positive from false-positive findings at the later steps of a multistep prognostic assessment.

Examining the baseline heterogeneity of our transdiagnostic population, we found functional-neurocognitive impairments in the ROD group akin to the CHR group and low between-group neuroanatomical and genetic separability (eTable 12 in the Supplement), supporting the neurobiological proximity between early-onset affective and psychotic disorders.^26,30,56^ Although CHR syndromes expectedly separated CHR and ROD groups at the cross-sectional level, we observed that these syndromes emerged in 19.2% of patients with ROD during the follow-up period, which led to psychosis transition in 1.8% of cases.^56^ Strikingly, our analyses also showed that the models’ prognostic accuracy, particularly the sensitivity for psychosis transition in patients with CHR, depended on patients with ROD being part of the model discovery process, which further supports the pooling of both groups into a broader risk population (eTable 14 and eFigure 12 in the Supplement). Our finding of a transdiagnostic predictability of psychosis was corroborated by the generalizability of the clinical-neurocognitive and neuroanatomical models to external samples, which showed markedly different risk levels, age distributions, and diagnostic compositions.

The in-depth analysis of the clinical-neurocognitive domain revealed that the presence of APSs facilitated a good baseline separability of CHR syndromes vs ROD and substantially informed the prediction of psychosis transition (eTable 13 in the Supplement). However, measures of childhood adversity,^46^ motor disturbances,^57^ and facial affect recognition^58^ did not overlap between diagnostic and prognostic models (eFigure 16 in the Supplement) and thus could be regarded as transdiagnostic markers^59,60,61,62^ of poor psychosis-related outcomes, including transition to psychosis. This interpretation was supported by the prognostic generalization of the clinical-neurocognitive model to the clinically relevant separation of patients with (1) nonremitting/de novo and nonsymptomatic CHR syndrome courses (eTable 15 in the Supplement) or (2) unfavorable perceptual, affective, functional, and basic symptom trajectories (eFigure 13 in the Supplement).^63,64^ Importantly, the model’s prognostic generalization capacity did not encompass nonpsychotic diagnoses (eTable 16 in the Supplement), and, thus, its prognostic pluripotency was confined to diverse CHR-specific symptom courses.^65^

Furthermore, we confirmed the prognostic value of PRS for schizophenia,^48^ as reported recently,^16^ and extended those findings by showing that genetic information augments the performance of clinical-neurocognitive models and prognostic workflows in a broader risk population (Table 2 and eTable 20 in the Supplement). Within this transdiagnostic setting, we replicated group-level differences among patients with psychosis transition, patients with nontransition, and healthy controls^16^ but also found that PRS-based prognostic assignments specifically differentiated APS-related trajectories (eFigure 13 in the Supplement).^66^ They also delineated patient groups with abnormally high and low genetic risk compared with healthy controls (eFigure 8 in the Supplement)—a finding that may point to distinct environmental and/or neurobiological pathways conferring risk and resilience to psychosis.^67^

The analysis of the structural neuroimaging data revealed a psychosis-predictive brain signature that generalized well across 3 independent cohorts. This signature overlapped with brain alterations previously reported to correlate with perceptual abnormalities, disorganization of speech and thought, and poor insight in early, subsyndromal, or prodromal stages of psychosis.^9,68,69,70^ Interestingly, nontransition-assigned patients showed reversed temporo-occipital volume reductions, which differentiated them from healthy controls (eFigure 9 in the Supplement). These findings may point to ongoing compensatory mechanisms of resilience to psychosis, as reported previously in a longitudinal sMRI study of adolescents with CHR.^71^ In this regard, our sMRI-based risk calculator may serve as a useful tool for enriching future observational studies and clinical trials with at-risk patients who express potential brain mechanisms of resilience to psychosis transition.

We observed that our raters matched unimodal risk calculators in predicting psychosis as measured by their BAC. However, raters also showed a pronounced optimism bias (low sensitivity and high specificity) toward the true risk of poor clinical outcome.^13^ It is noteworthy that their prognoses were based on all information collected in an extended study-related assessment and likely would be less accurate in routine, time-restricted diagnostic settings. Because the algorithmic counterparts showed exactly the inverted bias (high sensitivity and low specificity), the integration of clinicians and risk calculators into the cybernetic model produced a superior predictive system.^50^ Furthermore, our prognostic workflows demonstrated that similar levels of prognostic accuracy can be achieved by reducing the false-positive rate through sequential model application in patients with an estimated higher risk for psychosis transition (eFigure 14 in the Supplement). In this subgroup, the removal of the final sMRI-based assessment step increased false-positive findings (eFigure 15 in the Supplement), suggesting that the cost-benefit ratio of expensive neuromarkers needs to be individually adjusted according to the patient’s predicted risk.^72^

The finding that prognostic workflows always started with the clinical-neurocognitive model places the recognition of the clinical gestalt of emerging psychosis at the gateway of more precise early detection techniques.^73^ Our scalability experiments suggest that the laborious recognition of this pattern currently practiced in early recognition services could be effectively condensed to a few clinical-neurocognitive variables,^6^ thus enhancing the clinical utility of the proposed workflow. Nonetheless, future studies should revisit the validity of the selected 7 variables because they have been taken out of their original assessment context. Further studies also need to quantitatively explore the information patterns guiding clinicians’ gut-feeling estimates of psychosis transition and in turn foster more effective clinical early recognition strategies that integrate with cybernetic systems.

### Limitations

Psychosis transitions were limited to 26 individuals in the PRONIA discovery sample. This sample size increased the risk of producing overly optimistic prediction results owing to an accidental collection of well-classifiable cases. We implemented a multistep model validation procedure to guard against this possibility, including label permutation testing, strict nested cross-validation of all processing steps,^74^ in-depth model analysis to assess possible prognostic confounds and moderators, specificity testing of all models in a completely held-back portion of the PRONIA sample, and model benchmarking in 3 independent data sets, which provided a further 45 individuals with psychosis transition and 600 with nontransition for external validation. Owing to limited data availability in these samples, only the condensed clinical-neurocognitive, sMRI-based, and a specific stacked risk calculator trained on the outputs of the former 2 models could be externally validated. However, our internal-external validation approach followed established guidelines for model construction and validation.^21^ In keeping with this literature, the similar performance levels observed in our LOSOCV and independent validation experiments support the validity of the models not tested in external samples.

## Conclusions

In this prognostic study, we identified generalizable risk assessment tools that can be arranged into a multimodal prognostic workflow for a clinically viable, individualized prediction of psychosis in patients with CHR states and ROD. Our study showed for the first time, to our knowledge, that the augmentation of human prognostic abilities with algorithmic pattern recognition improves prognostic accuracy to margins that likely justify the clinical implementation of cybernetic decision-support tools. New international collaborations, such as the HARMONY (Harmonization of At Risk Multisite Observational Networks for Youth) initiative,^75^ may help to propel a reciprocal and iterative process of clinical validation and refinement of these prognostic tools in real-world early recognition services.

## Notes

Supplement. **eMethods.** Participants and Analysis **eTable 1.** Study Inclusion/Exclusion Criteria of the Study **eTable 2.** Antipsychotic Medication Thresholds Based on the Previous Version of the S3 Guidelines of the German Association for Psychiatry, Psychotherapy and Psychosomatics **eTable 3.** Group-Level Comparison of CHR, ROD and HC Individuals **eTable 4.** Diagnostic Breakdown of Psychotic Disorders in PRONIA Cases With a Disease Transition **eTable 5.** Sociodemographic, Clinical and Neurocognitive Variables Used in the Clinical Prediction Models **eTable 6.** MR Scanner Systems and Structural MRI Sequence Parameters Used at the Respective PRONIA Sites **eTable 7.** Effects of Baseline Treatments on the Decision Scores Generated by 5 Different Risk Calculators **eTable 8.** Correlations Between Decision Scores of Unimodal and Cybernetic Risk Calculators and Patients’ Maximum Follow-up Intervals and Number of Follow-up Examinations **eTable 9.** Analysis of the Site-Related Variation in Follow-up Duration, Time to Transition, Age and Sex Distributions in the PRONIA Cohort **eTable 10.** Effects of the Factor ‘Site’ on Predictive Performance of Raters, Unimodal, Stacked, and Cybernetic Risk Calculators **eTable 11.** Heterogeneity of Raters’ and Models’ Predictive Performance **eTable 12.** Differential Diagnostic Performance of Classification Models Trained to Separate Between CHR and ROD Patients Using Clinical-Neurocognitive, PRS-Based, and sMRI-Based Data Domain **eTable 13.** Explained Variances of Pairwise Prognostic, Diagnostic, and Prognostic-Diagnostic Classifier Combinations **eTable 14.** ROD Depletion and Substitution Analyses Assessing the Performance Effects Induced by the ROD Group in the Prediction of Psychosis Transitions in the CHR Sample **eTable 15.** Discriminative Performance of Raters and Risk Calculators in Distinguishing Between Transition Cases, Cases With Nonremitting/De novo CHR States and Cases Developing Asymptomatic CHR Trajectories **eTable 16.** Comparison of Nonpsychotic Diagnostic Outcomes Between Patients With a Predicted Transition vs Predicted Nontransition to Psychosis During the Follow-up Period of the Study **eTable 17.** Prognostic Sequences Tested in the Sequence Optimization Algorithm **eTable 18.** Study-Related, Sociodemographic, Physical, Functional, and Clinical Differences in the ZInEP Cohort **eTable 19.** Study-Related, Sociodemographic, Physical, Functional, and Clinical Differences in the BEARS-Kid Cohort **eTable 20.** Performance Gains Produced by the Stacking of the Clinical-Neurocognitive and PRS-Based Models **eFigure 1.** CONSORT Chart and Follow-up Protocol of the PRONIA Study for the Clinical Participants **eFigure 2.** Schematic Analysis Workflow of the Study **eFigure 3.** Experimental Design of the Machine Learning Pipelines Used to Train and Cross-validate the Unimodal and Stacked Risk Calculators **eFigure 4.** Schematic Representation of the NeuroMiner Model Optimization Process Used to Train the Structural MRI Predictors **eFigure 5.** Predictive Signature Underlying the sMRI-Based Risk Calculator **eFigure 6.** Comparison of Predictive Performance of Expert-Based, Unimodal, Stacked, Cybernetic and Sequentially Stacked Risk Calculators Trained and Cross-validated Using the PRONIA-18M and Complete PRONIA Cohorts **eFigure 7.** Comparison of Standardized Clinical-Neurocognitive Variables Between Healthy Volunteers and CHR/ROD Patients Who Were Labeled With a Transition or Nontransition to Psychosis by the Clinical-Neurocognitive Risk Calculator **eFigure 8.** Comparison of Polygenic Risk Scores (PRS) Between Healthy Volunteers and CHR/ROD Patients Who Were Labeled With a Transition or Nontransition to Psychosis by the PRS-Based Risk Calculator **eFigure 9.** Univariate Volumetric Comparisons Between Healthy Volunteers and Patients Labeled With a Transition or Nontransition to Psychosis by the sMRI-Based Risk Calculator **eFigure 10.** Image Quality Assessment of T1-Weighted Images Analyzed in the Study Using the Quality Assessment Tools of the CAT12 Toolbox **eFigure 11.** Interaction Analysis Assessing the Effects of the Number of Examinations Available and the Longest Interval Duration on the Predictions of Four Different Risk Calculators **eFigure 12.** Statistical Analysis of Prognostic Performance Effects Induced by the ROD Depletion and Substitution Strategies in the Three Unimodal Risk Calculators and the Stacked Model **eFigure 13.** Prognostic Stratification Effects of Raters’ Predictions, Unimodal and Cybernetic Risk Calculators on the Trajectories of CHR Syndromes and Functioning as Determined by Nonnegative Matrix Factorization and Linear Mixed-Effects Modeling **eFigure 14.** Detailed Analysis of the Optimal Sequential Prediction Algorithm **eFigure 15.** Regularization of the Prognostic Workflow for DIFFERENT LEVELS of Examination Sparsity and Analysis of Effects of Regularization on Prognostic Performance **eFigure 16.** Comparison of Clinical-Neurocognitive Signatures Used by the Prognostic Risk Calculator and the Differential Diagnostic Classifier (CHR vs ROD) **eReferences.**
