# Supplementary material for: Multimodal Machine Learning Workflows for Prediction of Psychosis in Patients With Clinical High-Risk Syndromes and Recent-Onset Depression
Source: JAMA Psychiatry. 2020 Dec 2;78(2):1–15. doi: 10.1001/jamapsychiatry.2020.3604 (PMC7711566; doi:10.1001/jamapsychiatry.2020.3604)
Supplement: Supplement. — eMethods. Participants and Analysis eTable 1. Study Inclusion/Exclusion Criteria of the Study eTable 2. Antipsychotic Medication Thresholds Based on the Previous Version of the S3 Guidelines of the German Association for Psychiatry, Psychotherapy and Psychosomatics eTable 3. Group-Level Comparison of CHR, ROD and HC Individuals eTable 4. Diagnostic Breakdown of Psychotic Disorders in PRONIA Cases With a Disease Transition eTable 5. Sociodemographic, Clinical and Neurocognitive Variables Used in the Clinical Prediction Models eTable 6. MR Scanner Systems and Structural MRI Sequence Parameters Used at the Respective PRONIA Sites eTable 7. Effects of Baseline Treatments on the Decision Scores Generated by 5 Different Risk Calculators eTable 8. Correlations Between Decision Scores of Unimodal and Cybernetic Risk Calculators and Patients’ Maximum Follow-up Intervals and Number of Follow-up Examinations eTable 9. Analysis of the Site-Related Variation in Follow-up Duration, Time to Transition, Age and Sex Distributions in the PRONIA Cohort eTable 10. Effects of the Factor ‘Site’ on Predictive Performance of Raters, Unimodal, Stacked, and Cybernetic Risk Calculators eTable 11. Heterogeneity of Raters’ and Models’ Predictive Performance eTable 12. Differential Diagnostic Performance of Classification Models Trained to Separate Between CHR and ROD Patients Using Clinical-Neurocognitive, PRS-Based, and sMRI-Based Data Domain eTable 13. Explained Variances of Pairwise Prognostic, Diagnostic, and Prognostic-Diagnostic Classifier Combinations eTable 14. ROD Depletion and Substitution Analyses Assessing the Performance Effects Induced by the ROD Group in the Prediction of Psychosis Transitions in the CHR Sample eTable 15. Discriminative Performance of Raters and Risk Calculators in Distinguishing Between Transition Cases, Cases With Nonremitting/De novo CHR States and Cases Developing Asymptomatic CHR Trajectories eTable 16. Comparison of Nonpsychotic Diagnostic Outcomes Between [file jamapsychiatry-e203604-s001.pdf]

Supplementary Online Content

Koutsouleris N, Dwyer DB, Degenhardt F, et al; Writing Group for the PRONIA Consortium. Multimodal workflow in machine learning workflows for prediction of psychosis prediction in patients with clinical high-risk syndromes and recent-onset depression. *JAMA Psychiatry*. Published online December 2, 2020. doi:10.1001/jamapsychiatry.2020.3604

eMethods. Participants and Analysis
eTable 1. Study Inclusion/Exclusion Criteria of the Study
eTable 2. Antipsychotic Medication Thresholds Based on the Previous Version of the S3 Guidelines of the German Association for Psychiatry, Psychotherapy and Psychosomatics
eTable 3. Group-Level Comparison of CHR, ROD and HC Individuals
eTable 4. Diagnostic Breakdown of Psychotic Disorders in PRONIA Cases With a Disease Transition
eTable 5. Sociodemographic, Clinical and Neurocognitive Variables Used in the Clinical Prediction Models
eTable 6. MR Scanner Systems and Structural MRI Sequence Parameters Used at the Respective PRONIA Sites
eTable 7. Effects of Baseline Treatments on the Decision Scores Generated by 5 Different Risk Calculators
eTable 8. Correlations Between Decision Scores of Unimodal and Cybernetic Risk Calculators and Patients’ Maximum Follow-up Intervals and Number of Follow-up Examinations
eTable 9. Analysis of the Site-Related Variation in Follow-up Duration, Time to Transition, Age and Sex Distributions in the PRONIA Cohort
eTable 10. Effects of the Factor ‘Site’ on Predictive Performance of Raters, Unimodal, Stacked, and Cybernetic Risk Calculators
eTable 11. Heterogeneity of Raters’ and Models’ Predictive Performance
eTable 12. Differential Diagnostic Performance of Classification Models Trained to Separate Between CHR and ROD Patients Using Clinical-Neurocognitive, PRS-Based, and sMRI-Based Data Domain
eTable 13. Explained Variances of Pairwise Prognostic, Diagnostic, and Prognostic-Diagnostic Classifier Combinations
eTable 14. ROD Depletion and Substitution Analyses Assessing the Performance Effects Induced by the ROD Group in the Prediction of Psychosis Transitions in the CHR Sample
eTable 15. Discriminative Performance of Raters and Risk Calculators in Distinguishing Between Transition Cases, Cases With Nonremitting/De novo CHR States and Cases Developing Asymptomatic CHR Trajectories
eTable 16. Comparison of Nonpsychotic Diagnostic Outcomes Between Patients With a Predicted Transition vs Predicted Nontransition to Psychosis During the Follow-up Period of the Study
eTable 17. Prognostic Sequences Tested in the Sequence Optimization Algorithm
eTable 18. Study-Related, Sociodemographic, Physical, Functional, and Clinical Differences in the ZInEP Cohort
eTable 19. Study-Related, Sociodemographic, Physical, Functional, and Clinical Differences in the BEARS-Kid Cohort

© 2020 Koutsouleris N et al. *JAMA Psychiatry.*

eTable 20. Performance Gains Produced by the Stacking of the Clinical-Neurocognitive and PRS-Based Models

eFigure 1. CONSORT Chart and Follow-up Protocol of the PRONIA Study for the Clinical Participants
eFigure 2. Schematic Analysis Workflow of the Study
eFigure 3. Experimental Design of the Machine Learning Pipelines Used to Train and Cross-validate the Unimodal and Stacked Risk Calculators
eFigure 4. Schematic Representation of the NeuroMiner Model Optimization Process Used to Train the Structural MRI Predictors
eFigure 5. Predictive Signature Underlying the sMRI-Based Risk Calculator
eFigure 6. Comparison of Predictive Performance of Expert-Based, Unimodal, Stacked, Cybernetic and Sequentially Stacked Risk Calculators Trained and Cross-validated Using the PRONIA-18M and Complete PRONIA Cohorts
eFigure 7. Comparison of Standardized Clinical-Neurocognitive Variables Between Healthy Volunteers and CHR/ROD Patients Who Were Labeled With a Transition or Nontransition to Psychosis by the Clinical-Neurocognitive Risk Calculator
eFigure 8. Comparison of Polygenic Risk Scores (PRS) Between Healthy Volunteers and CHR/ROD Patients Who Were Labeled With a Transition or Nontransition to Psychosis by the PRS-Based Risk Calculator
eFigure 9. Univariate Volumetric Comparisons Between Healthy Volunteers and Patients Labeled With a Transition or Nontransition to Psychosis by the sMRI-Based Risk Calculator
eFigure 10. Image Quality Assessment of T1-Weighted Images Analyzed in the Study Using the Quality Assessment Tools of the CAT12 Toolbox
eFigure 11. Interaction Analysis Assessing the Effects of the Number of Examinations Available and the Longest Interval Duration on the Predictions of Four Different Risk Calculators
eFigure 12. Statistical Analysis of Prognostic Performance Effects Induced by the ROD Depletion and Substitution Strategies in the Three Unimodal Risk Calculators and the Stacked Model
eFigure 13. Prognostic Stratification Effects of Raters' Predictions, Unimodal and Cybernetic Risk Calculators on the Trajectories of CHR Syndromes and Functioning as Determined by Nonnegative Matrix Factorization and Linear Mixed-Effects Modelling
eFigure 14. Detailed Analysis of the Optimal Sequential Prediction Algorithm
eFigure 15. Regularization of the Prognostic Workflow for DIFFERENT LEVELS of Examination Sparsity and Analysis of Effects of Regularization on Prognostic Performance
eFigure 16. Comparison of Clinical-Neurocognitive Signatures Used by the Prognostic Risk Calculator and the Differential Diagnostic Classifier (CHR vs ROD)
eReferences.
This supplementary material has been provided by the authors to give readers additional information about their work.

© 2020 Koutsouleris N et al. *JAMA Psychiatry.*

eMethods. Participants and Analysis

1.1. Inclusion and exclusion criteria, study protocol and CONSORT chart

The study inclusion and exclusion used to recruit patients with clinical high-risk states for psychosis, patients with recent-onset depression (ROD), and healthy volunteers across the seven PRONIA sites are described in **eTable 1**. The geographical distribution of the study sites, the follow-up scheme of the study, and the CONSORT chart are shown in **eFigure 1**.

For the present study, the PRONIA consortium screened 5547 individuals for study eligibility between 2/15/2014 and 5/1/2017 across seven academic early recognition services which cover a European catchment population of 5,384,000 persons. Among the screened cohort, 263 patients met inclusion criteria for CHR states, and 246 patients fulfilled criteria for a ROD. Ninety-six candidate CHR patients and 79 ROD patients had to be excluded from this cohort because (1) inclusion criteria could not be validated, (2) patients met exclusion criteria (**eTable 1**), including maximum medication thresholds (**eTable 2**), (3) did not finish the baseline examination, or were lost to follow-up after the 6-month visit. From the remaining study population of 167 CHR and 167 ROD individuals, a risk calculator discovery cohort (N=246) was extracted that comprised patients who had been followed for at least 18 months (PRONIA+18M sample) and therefore provided us with higher certainty concerning their transition-related outcomes.[1] The rest of the study population (N=88, PRONIA-18M sample), which consisted of non-transition cases only, was used as an independent validation sample to further validate the specificity of the trained risk prediction models. The rationale for this split of the PRONIA database, was two-fold: First, we were able to assess the generalizability of our models in a population which was lost earlier to follow-up and thus more realistically approximated the real-world situation of early recognition services. Second, all predictive data domains were available in the PRONIA-18M validation cohort, in contrast to the external samples described below, which only partially overlapped with the PRONIA feature space. This complete overlap allowed us to validate the human raters' prognostic performance, the unimodal and multimodal risk calculators, the cybernetic model, and the prognostic sequence.

To train and validate the structural MRI-based risk calculator, the study analyzed the baseline imaging data of a total 326 CHR and ROD individuals (discovery sample: 126 CHR/116 ROD patients, validation sample: 39 CHR/45 ROD patients), which were acquired at baseline based on a minimally harmonized structural MRI protocol (**eTable 6**) and which passed the quality control as described in section 1.3. For the genetic risk calculator, the DNA of 298 patients (discovery sample: 116 CHR/110 ROD patients, validation sample: 39 CHR/33 ROD patients) could be successfully extracted from the blood samples and genotyped as described in section 1.4. In addition, we extracted 334 healthy controls (HC) from the PRONIA database, which were matched one-to-one for age, sex, and site with the 334 clinical participants. Baseline study groups were compared in **eTable 3**.

1.2. Clinical and neurocognitive features

For the clinical and neurocognitive modelling part of the analysis, we used established assessment tools capturing phenotypes that were previously reported to be predictive of transition to psychosis.[2] The description of variables measuring these phenotypes are provided in **eTable 5**Error! Reference source not found., and, in summary, covered the following five phenotypic domains:

i. Sociodemographic variables consisting of age and sex,

ii. Interview-based psychopathological symptom assessments (Structured Interview for Psychosis-Risk Syndromes[3] with positive, negative, disorganized and general subscale items; Schizophrenia Proneness Instrument – Adult and Child/Youth version,[4] with items measuring cognitive attentional impediments (*B* items), cognitive disturbances (*C* items), disturbances in experiencing the self and surroundings (*D* items), perception disturbances (*F* items), and optional items with a positive predictive value of equal

© 2020 Koutsouleris N et al. *JAMA Psychiatry.*

or greater 0.70 according to the prospective Cologne Early Recognition study (*O* items). Item scores of 7 (=symptom item has always been present in same severity), 8 (=symptom item definitely present but severity unknown), and 9 (=symptom definition questionably met) were set to 0 before entering subsequent analyses,

iii. Interview-based current, previous and lifetime levels of functioning (Global Assessment of Functioning – Split Version[5]; Global Functioning: Social and Role scales[6]),

iv. Self-reported childhood trauma as measured by the 28 items of the Childhood Trauma Questionnaire (CTQ).[7] Single CTQ items were preferred over CTQ domain scores to allow for a data-driven weighting and combination of trauma-related information with other phenotypic data.

v. Neurocognitive performance in the general intelligence (Wechsler Adult Intelligence Scale, WAIS[8]), processing speed (Digit-Symbol Substitution Test (DSST) from the Brief Assessment of Cognition in Schizophrenia[9]; Trail Making Test A, TMT-A[10]), (visual) working memory (Self-Ordered Pointing Task, SOPT[11]; Rey-Osterreith Figure, ROCF[12]; digits forward and backward tests[8]), verbal fluency (Semantic and Phonetic Verbal Fluency, SVF/PVF[13]), cognitive flexibility, sustained attention and inhibitory control (Trail-Making Test B, TMT-B[14]), and facial emotion recognition as a measure of the social cognitive domain (Diagnostic Analysis of Non-Verbal Accuracy, DANVA[15]).

1.3. MRI data acquisition and processing

When setting up the PRONIA study, we decided to record data that represents the MR scanner sequence heterogeneity encountered in real clinical settings. The aim of this strategy was to strengthen the generalizability and clinical applicability of the risk calculators developed in our machine learning analyses. Thus, a minimal MRI harmonization protocol was implemented across the PRONIA consortium that required the sites to only (1) acquire isotropic or nearly isotropic voxel sizes of preferably 1 mm resolution, (2) set the Field Of View (FOV) parameters as needed in order to guarantee the full 3D coverage of the brain including all parts of the cerebellum, and (3) define the relaxation time (TR) and echo time (TE) as well as other imaging parameters in a way that would maximize the contrast between cortical ribbon and the white matter and enhance the signal-to-noise ratio in the images. The parameters defining the structural MR sequences across the 7 PRONIA sites are listed in **eTable6**.

At each PRONIA site, all 660 images (329 clinical participants, 331 healthy controls) were visually inspected, automatically defaced, and anonymized using an in-house Freesurfer-based script prior to data centralization. Then, the open-source CAT12 toolbox (version r1155; http://dbm.neuro.uni-jena.de/cat12/), an extension of SPM12[16] designed for the processing and analysis of structural brain images, was used to segment images into grey matter (GM), white matter, and cerebrospinal fluid maps, and then to high-dimensionally register them to the stereotactic space of the Montreal Neurological Institute (MNI-152 space). The manual of the CAT12 toolbox (http://www.neuro.uni-jena.de/cat12/CAT12-Manual.pdf) details all processing steps applied to the structural images. In summary, they consisted of (1) the 1st denoising step based on Spatially Adaptive Non-Local Means (SANLM) filtering;[17] (2) an Adaptive Maximum A Posteriori (AMAP) segmentation technique, which models local variations of intensity distributions as slowly varying spatial functions and thus achieves a homogeneous segmentation across cortical and subcortical structures;[18] (3) the 2nd denoising step using Markov Random Field approach which incorporates spatial prior information of adjacent voxels into the segmentation estimation generated by AMAP;[18] (4) a Local Adaptive Segmentation (LAS) step, which adjusts the images for white matter (WM) inhomogeneities and varying gray matter (GM) intensities caused by differing iron content in e.g. cortical and subcortical structures. The LAS step is carried out before the final AMAP segmentation; (5) a partial volume segmentation algorithm that is capable of modeling tissues with intensities between GM and WM, as well as GM and cerebrospinal fluid (CSF) and is applied to the AMAP-generated tissue segments; (6) a high-dimensional DARTEL registration of the image to a MNI-template generated from the MRI data of 555 healthy controls in the IXI database

(http://www.braindevelopment.org). The registered GM images were multiplied with the Jacobian determinants obtained during registration to produce GM volume (GMV) maps. GMV maps were resliced to 3 mm isotropic voxel resolution and smoothed with a 4 to 8 mm full-width-at-half maximum (FWHM) Gaussian kernel, with the kernel width being optimized in the subsequent machine learning analysis (see section **1.5.3**). The Quality Assurance framework of CAT12 was used to quantitatively check the quality of the GMV maps (**eFigure 10, A**). This procedure produced a weighted quality score (from excellent [A] to failed [F]) for each image, which for 95.7% of the images used in the current study fell between a B+ (81.6%) and B (14.1%). We excluded two images because they were rated with a C [satisfactory] due to white matter segmentation errors.

1.4. Genotyping

DNA could be extracted from the whole blood samples of 499 participants (25 PT and 273 NT patients [89.2% of the clinical study cohort], 209 healthy controls) who had provided blood for, and consented to, genetic testing. DNA was genotyped using Illumina's Infinium Global Screening (GSA) Array-24 BeadChip version 2 + Psych content (GSA). The GSA includes > 650,000 markers and offers an unparalleled genomic coverage and imputation performance. The Psych content comprises 50,000 variants associated with common psychiatric disorders such as schizophrenia, bipolar disorder, and autism spectrum disorders. After standard, stringent quality control using PLINK[19] (e.g. sample call rate > 0.98; variant call rate > 0.98; Minor Allele Frequency > 0.01; removal of variants deviating from Hardy-Weinberg equilibrium, p < 10E-6; sex check and heterozygosity outlier analysis), a total of 505,687 variants remained in the dataset.

Subsequently, the dataset was imputed using the Haplotype Reference Consortium panel (rv1.1; www.haplotype-reference-consortium.org; n=32,470 samples) using Positional Burrows-Wheeler Transform as implemented in the Sanger Imputation Server (imputation.sanger.ac.uk). To include reliable variants for polygenic risk score analysis we excluded imputed variants with lower prediction accuracy (i.e., info score<0.6). After successful imputation, more than 7,420,913 variants were available in the PRONIA dataset.

Finally, we computed Polygenic Risk Scores for schizophrenia (PRS-SCZ) by means of the "clumping plus threshold" method employing the summary statistics from the SCZ genome-wide association studies meta-analysis as provided by the Psychiatric Genomic Consortium (PGC2, https://www.med-.unc.edu/pgc/download-results/; n>36,989 patients and n>113,075 controls). Clumping was performed using PLINK to filter for independent genetic signals (r^2<0.1) within the target dataset having the highest association with SCZ summary statistics considering non-overlapping 250kb size regions across the genome. PRS-SCZ were computed as the sum of the risk alleles weighed by the association estimates for SCZ (beta of PGC-SCZ2) including all common variants (i.e., with Minor Allele Frequency > 1%) in the clumped dataset at a given *P* value threshold. PRS were generated over a range of ten *P* values from genome-wide significant threshold to the full model (i.e., 5.0E-8, 3.2E-7, 2.1E6, 1.4E-5, 8.8E-5, 5.6E-4, 3.7E-3, 2.4E-2, 1.5E-1, 1) thus including an increasing number of variants (i.e., 155, 232, 389, 773, 1640, 1874, 11255, 35586, 119001, 324299). PRS were standardized to have a direct comparison across sample PRS values considering variable *P* value thresholds. Moreover, since PRS values can be also influenced by population structure we extracted the first 10 Principal Components (PC) from the post QC genotype data. Pruning of genotyping data was applied prior to computation the PC to limit the effect of Linkage Disequilibrium (LD) across markers and thus to better represent the population structure in the eigenvectors. Genome-wide pruning was performed with PLINK considering window sizes of 50 variants and steps of 5 variants while a threshold of 0.5 in the R^2 correlation across paired variants was considered. Both the 10 PC to consider population structure and the 10 PRS-SCZ to model the genetic liability for schizophrenia were included in the machine learning analysis described below.

© 2020 Koutsouleris N et al. *JAMA Psychiatry.*

1.5. Machine Learning

1.5.1. Overall strategy

In the **first step** of our machine learning strategy (see **eFigure 2** and **section 1.5.3**), we trained an optimal sequential prognostic workflow for psychosis risk stratification that integrated unimodal and multimodal (stacked) algorithmic predictions with expert-based prognostic estimates in the PRONIA discovery sample (PRONIA+18M). To this end, we developed a sequential prognostic algorithm that identified the optimal sequence of predictive components to be combined into a stacked model,[20] as well as the optimal decision thresholds determining the cases to be propagated to the next predictive component in the sequence (see **section 1.5.4**). We tested the optimal prognostic algorithm as well as its predictive components by assessing their generalizability to geographically distinct patient samples following the *internal-external validation* approach recommended by Steyerberg and Harrell.[21] In our case, this approach took the form of a repeated nested leave-site-out cross-validation (LOSOCV) scheme, in which the inner pooled cross-validation cycle (CV_1) trained and optimized the prognostic workflow's component models, and the workflow itself, while the outer leave-site-out cross-validation cycle (CV_2) iteratively tested their generalizability to each held-back validation site (**eFigure 3**). This LOSOCV scheme captured the geographic transportability of our models within a European context because it measured model generalizability against multiple sources of population variance caused by the multi-center design of PRONIA (e.g. varying pathways to and organizational forms of mental healthcare systems, diverse genetic backgrounds of patients recruited at the different PRONIA sites, different scanner hardware and software, etc.). First, we assessed whether significant site-related variation existed in the follow-up duration, the age and sex distributions of our study population, as well as the time to transition in patients who developed psychosis (**eTable 9**). Then, we tested explicitly whether our models' prognostic estimates were moderated by image quality (**eFigure 10**), baseline treatments or treatment history (**eTable 7**), follow-up duration and availability (**eTable 8**, **eFigure 11**), as well as by site-related effects (**eTable 10**). Furthermore, our internal-external validation approach was extended to include a conservative validation of *model specificity* by applying *all* risk calculators and the prognostic workflow trained in the PRONIA+18M sample to the PRONIA-18M cohort (**eFigure 2**), which included non-transition patients with shorter follow-up intervals compared to the discovery cohort (**Table 1**, main manuscript).

The **second step** of the analytical strategy was to gain a deeper insight into the signatures of the prognostic workflow models by:

(1) Extracting their reliable and significantly predictive elements using cross-validation ratio and sign-based consistency mapping (see **section 1.5.5**),

(2) Assessing whether the predictive signatures of clinical-neurocognitive, sMRI- and PRS-based models represented a deviation from normality by comparing the predicted outcome groups to age-, sex- and site-matched health volunteers (see **section 1.5.6**),

(3) (a) Exploring whether our risk calculators and clinical raters were influenced by study-group related clinical-neurocognitive, PRS-based, and imaging-related differences between the CHR and ROD patients,
(b) Evaluating whether the inclusion of the ROD group into the training of unimodal and multimodal risk calculators improved prediction performance in the CHR sample. These analyses are described in **section 1.5.7**.

(4) (a) Evaluating whether the estimates generated by the workflow components were not only predictive of psychosis but also predictive of psychiatric diagnostic outcomes more broadly,
(b) Assessing whether these estimates delineated distinct CHR syndrome trajectories which were constructed from the patients' longitudinal data using unsupervised machine learning methods and linear mixed models (see **section 1.5.8**).

© 2020 Koutsouleris N et al. *JAMA Psychiatry.*

In the **third analytic step**, we assessed the significance of our models' performance because of the limited number of transition cases and the imbalance of the outcome classes in our study sample. To maximize the sample size for this and the subsequent analysis step (external validation), we first re-trained all risk calculators and evaluated their LOSOCV performance using the entire PRONIA cohort (N=334 cases). Then, we performed outcome label permutations and compared the permuted component risk calculators' and the workflow's leave-site-out performance against the respective models trained using the original labels.[22] **Section 1.5.9** details this permutation analysis and **Table 2** in the main manuscript reports the results.

In the **final step** of our analysis strategy, we tested the *external validity* of several workflow components, such as a condensed version of the clinical-neurocognitive risk calculator, the sMRI-based risk calculator, and a stacked risk calculator that analyzed the predictions of both former models. To this end, we applied the risk calculators retrained on the entire PRONIA sample to patients drawn from completely independent projects– the ZInEP,[23] FePsy,[24] and BEARS-Kid studies.[25]

1.5.2. Data domain-specific feature preprocessing

As outlined in **eFigure 3**, each data domain underwent specific preprocessing steps as part of model optimization. Importantly, to exclude any information leakage between the training, test, and validation samples, NeuroMiner wraps all preprocessing steps entirely into a nested cross-validation design. Within this design the CV_1 training samples are used to compute preprocessing parameters (e.g. the mean and standard deviation of a z-normalization model), the CV_1 test data are employed to select the optimally predictive model parameters, and the CV_2 validation data serve to determine the leave-site-out generalizability of the models. Thus, the preprocessing and subsequent machine learning steps are jointly optimized and become integrated parts of the predictive model. **eFigure 4** exemplifies this process with respect to the training of the sMRI-based risk calculator.

For the *interview-based, patient-reported and neurocognitive data*, the preprocessing involved the following procedures: (1) feature-wise scaling of the data matrix to the range [0,1], followed by (2) k Nearest Neighbor (kNN)-based imputation using the Jaccard distance as dissimilarity measure, and (3) standardization by mean-centering each feature and dividing it by the respective feature's standard deviation. The percentage of missing values in data matrix measured 3.2%.

For the *sMRI data*, the GMV maps were resliced to a 3 mm isotropic voxel resolution and smoothed with 4, 6 and 8 mm Full-Width-at-Half-Maximum Gaussian kernels (**eFigure 4**). Then, the resliced and smoothed maps were age-corrected using partial correlation analysis. The smoothed, resliced and age-corrected GMV maps were thresholded for between-scanner voxel reliability, as described previously.[26] In brief, we applied generalizability theory[27] to the GMV maps of six travelling healthy volunteers to produce a *g* coefficient map that measured the voxels' between-site reliability. This map was then resliced and smoothed as described above and applied to the patients' smoothed, resliced and age-adjusted GMV maps to remove unreliable voxels at the 25%, 50% and 75% percentile thresholds. Finally, the dimensionality of the thresholded maps was reduced by means of Principal Component Analysis (PCA).[28] PCA projected the image information to 15, 20 and 25 eigenvariates. The patients' eigenvariate scores were standardized and then forwarded to the wrapper-based machine-learning algorithm described below.

For the *genetic data* domain, we used partial correlation analysis to correct the PRS score matrix feature-wise for ancestry-related variance using the 10 PCA-based ancestry components. The corrected PRS scores were then standardized before entering the subsequent machine learning analysis steps.

1.5.3. Machine learning algorithms and feature selection

Wrapper-based feature selection strategies[29] using linear, non-kernelized L2-regularized, L1-loss support vector machines (SVM; provided by the LIBLINEAR library[30] in NeuroMiner) were used to find the

optimal set of predictive features for the three unimodal risk calculators. We employed cost-sensitive SVM formulations to automatically account for the highly unbalanced class distribution in our study population. Models were trained using the CV_1 training data first and then selected based on their prediction performance in the entire CV_1 data (training + test data) partition. More specifically, for each hyperparameter combination and CV_1 partition the wrapper-based feature selection process was repeated, thus producing a bag of models with variable feature sets. We picked optimally predictive models from this bag by first computing the SVM models' average balanced accuracy (BAC), defined as:

$$\overline{\mathrm{BAC}}_{\mathrm{CV_1\,Train+CV_1\,Test}} = \frac{\sum_{i=1}^{k=5\times10}(\mathrm{Sensitivity}_i + \mathrm{Specificity}_i)/2}{k}$$

across the k=50 (=5 repetitions × 10 folds) models available in the bag for each hyperparameter combination. Then, we combined these performance measures into a hyperparameter performance profile as shown in **eFigure 4**. The number of parameter combinations tested during model optimization varied per data domain, with the optimal clinical and PRS-based models being selected within the SVM's *C* regularization parameter range of $2^{[-4 \underset{\in\mathbb{Z}}{\rightarrow} +4]}$, and the best structural MRI models being picked within a hyperparameter space defined by the following four dimensions:

i. Three Gaussian smoothing kernel widths of 4, 6 and 8 mm,
ii. three *g* map percentile thresholds at 25%, 50%, and 75%,
iii. three dimensionalities defined for data reduction using PCA: 15, 20 and 25 principal components, and
iv. five SVM *C* parameters picked from the range of 2^{-4} to 2^{+4} with an exponent stepping of 2,

totaling up to 135 parameter combinations.

The wrapper-based feature selection strategies varied per data domain: For the optimization of the clinical-neurocognitive risk calculator, we performed a greedy sequential forward search (SFS)[29] at each SVM *C* regularization parameter with early stopping at 10% of the features selected. The rationale was to find a restricted feature set among the 141 input variables (see **eTable 5**) that would most economically predict outcome labels, and hence would be more suitable for real-world application than the full feature set. After validating the model using LOSOCV, we used sign-based consistency mapping (section 1.5.5) to identify significant variables and retrained the risk calculator using only those. This reduced model was then externally validated in the ZInEP and BEARS-Kid samples and became part of a clinically scalable prognostic workflow (see section **1.5.4**).

Similarly, we applied SFS to the processed imaging data to select parsimonious combinations of principal components that were most predictive of transition to psychosis. To mitigate the risk of overfitting, we did not apply the SFS algorithm at all 135 parameter combinations of the optimization problem. Instead, in each CV_1 partition, we selected the best three SVM models across this parameter range and optimized them using SFS. To further reduce the risk of overfitting, this feature selection process stopped when the prediction performance increase started leveling out, as detected by means of a knee-point detection algorithm, or when 50% of the features had been selected—we used 50% instead of 10% because, unlike for clinical and neurocognitive data that are laborious to collect, there is no clinical need to maximally reduce the feature set from an MRI scan.

Finally, we used greedy sequential backward elimination (SBE)[29] in the ancestry-adjusted PRS data to remove noise and variance unrelated to the prediction task. The SBE algorithm is less aggressive than the greedy forward search approach used for the clinical-neurocognitive and imaging domains and thus more suitable for feature sets characterized by high a degree of collinearity. The SBE search was stopped when 50% of the PRS features had been removed from the feature pool.

To further reduce the risk of wrapper overfitting, we did not select the winning model from the $\overline{\mathrm{BAC}}_{\mathrm{CV_1\,Train+CV_1\,Test}}$ profile, but picked the best three ensemble models, which were retrained using the entire CV_1 partition data and then combined into a bag of SVM ensembles (see **eFigure 4**). This

ensemble bag was then applied without any further modification to the CV_2 validation data of given held-back PRONIA site, thus producing decision scores D_{Ens} for each CV_2 validation patient, which were further accumulated across the CV_2 leave-site-out partitions to produce a bag of leave-site-out decision scores. This bagged ensemble finally predicted a CV_2 patient's outcome based on the class membership probability calculated by means of majority voting.[31] The median across the ensemble of decision scores constituted the Out-Of-Training (OOT) decision score of each CV_2 validation patient.

Then, we built three different stacking models that integrated modality-specific models as follows:

i. a model combining the full clinical-neurocognitive, sMRI-based, and PRS-based models,
ii. a model combining clinical-neurocognitive, sMRI-based, PRS-based, and human expert predictions, and finally
iii. a stacked model combining the condensed clinical-neurocognitive model and the sMRI model for external validation (see section **1.5.10**).

For the training of the stacked models, we employed linear, kernelized L2-regularized, L1-loss SVMs as provided by the LIBSVM library without wrapper-based feature selection.[32] Input features of the stacking algorithm were the CV_1-test decision scores, which were extracted across the three unimodal outcome predictors and aggregated into a new training data matrix using the same CV_1 cross-validation structure as for the training of the unimodal classifiers. This new CV_1-training score matrix was scaled to the range [-1, 1] using its column-wise minima and maxima and the resulting scaling model was then applied to the respectively aggregated CV_1-test and CV_2-validation score matrices. Due to cases with missing data in the PRS and structural MRI domains, we applied kNN-based imputation to the training, test and validation matrices as described above for the preprocessing of the clinical-neurocognitive data domain. Iterating through the CV_1 cycle, the scaled and imputed training data matrices were forwarded to the SVM algorithm which found the optimal *C* parameter within the range of $2^{[-4 \underset{\in \mathbb{Z}}{\rightarrow} +4]}$ using the same optimization process as in the unimodal classification analyses. The final outcome prediction for the CV_2 validation patients was carried out as described above.

Furthermore, following the principles of expert-augmented machine learning,[33] we extended this algorithmic stacking procedure by incorporating the raters' prognostic estimates into the decision score matrix. These estimates were a coded as yes/no replies to the question 'Do you think that the patient is likely to transition to psychosis?', which our raters received at the end of the baseline examination. Inspired by Norbert Wiener's foundational work on cybernetics,[34,35] we will use the term 'cybernetic risk calculator' throughout this work to differentiate this type of stacked risk calculator from purely algorithmic multi-modal prediction.

1.5.4. Case propagation-based sequential stacking

Building on our previous work,[26] we implemented a sequential stacking algorithm in NeuroMiner to generate cost-effective prognostic machine learning tools for clinical care. The algorithm optimizes the application sequence of predictive models that maximizes prognostic accuracy and reduces the per-case examinations needed to achieve this performance. More specifically, the objective of the sequence optimizer is to search through a space of every combination of stacking possibilities (see **eTable 17**), while intelligently choosing individuals for whom prognostic (or diagnostic) accuracy would improve from the addition of new modalities in the stack. The combination of stacking possibilities was divided into four different sequential nodes that represented unimodal predictions, predictions based on two data modalities, predictions based on three modalities, and predictions based on four modalities (i.e., the decision scores produced by the clinical-neurocognitive, PRS-based, and sMRI-based risk calculators, as well as by the estimates provided by the clinical raters). To identify individuals who need additional sequences, we employed a method that focuses on maximizing the decision score margin between the cases of the opposite classes within specific percentile windows.

© 2020 Koutsouleris N et al. *JAMA Psychiatry.*

Specifically, for a given predictive sequence (**eTable 17**), the algorithm first ranks the training cases according to their decision scores in the prediction node, and then, starting from the decision boundary with a 5%-step width, determines the optimal upper and lower decision score percentiles for which case propagation to the next prediction node would maximize the decision score margin between cases of opposite classes. Establishing increasing percentile windows around the decision boundary is expected to place a gradient from individuals with most ambiguous decision scores to individuals with a very unequivocally predicted class membership. This procedure is repeated across all subsequent prognostic nodes of the given sequence, for all prognostic sequences to be tested, and for each CV_1 data partition so that a performance profile can be computed across the three-dimensional hyperparameter cube of the algorithm, i.e. the sequence pool, the lower (L) and the upper (U) case propagation percentiles. In the current work, we defined 64 candidate sequences (**eTable**) and the L(-)/U(+) thresholds around the decision boundary ([±15%, ±25%, ±50%] of cases), thus resulting in 64 × 3 × 3 = 574 hyperparameter combinations. The winning prognostic sequence was defined by the hyperparameter combination that was most frequently chosen by the algorithm based on the BAC as optimization criterion (**eFigure 14, I**). This sequence was further analyzed in terms of the number, percentage of propagated PT and total cases, predicted classes, and prognostic performance per examination node (**eFigure 14, II**).

Furthermore, to facilitate translation into clinical care, we tested whether the integration of sparsity regularization in the sequence optimization process would reduce the number of examinations needed to achieve similar levels of prognostic performance as in the non-regularized sequence. Examination cost was operationalized as the fraction of cases reaching the final examination step of the given sequence. More specifically, following the regularization approach proposed by Boardman and Trappenberg[36], we down-weighted BAC by examination cost as follows:

$$\widehat{BAC_S} = \frac{\text{Sensitivity}+\text{Specificity}}{2} - \lambda(\frac{N_c}{N})^\Gamma$$

Where N_c defined the number of individuals among the total N of cases that reached the final examination step of sequence S. The regularization parameter λ was always kept at 1, while regularization strength Γ was set to either 0.5 or 1.0, thus imposing increasing levels of examination sparsity on the optimal sequence identification process (**eFigure 15, A**). The OOT performance of the two new optimized prognostic sequences was measured using leave-site-out cross-validation in the PRONIA+18M and complete PRONIA cohorts and displayed in the in terms of false positive rate, sensitivity, specificity and BAC in **eFigure 15, C1-C2**. Furthermore, we compared the CV_2-level BAC measures of the two new sequences to the BAC of the non-regularized workflow using the Wilcoxon paired signed rank test (**eFigure 15**, **C2**). In addition, we analyzed the fraction of patients reaching each examination node (**eFigure 15**, **B**) to assess the effect of regularization strength on examination sparsity. Finally, we tested whether the replacement of the full clinical-neurocognitive model with the condensed version negatively impacted on the regularized and non-regularized workflows' false positive rate in the PRONIA-18M sample (**eFigure 15**, **C1**).

1.5.5. Visualization of predictive patterns elements using ensemble learning

Two computational approaches, as implemented in NeuroMiner, were used to visualize the predictive patterns elements in the unimodal and stacked risk calculators trained on the PRONIA+18M sample. First, we calculated the pattern element *stability*, termed cross-validation ratio (CVR), by computing the mean and standard error of all SVM weight vectors concatenated across the entire nested cross-validation structure (**eFigure 3**). This cross-validation ratio as a measure for pattern stability was inspired by the bootstrap ratio commonly used in the Partial Least Squares literature and described in

© 2020 Koutsouleris N et al. *JAMA Psychiatry.*

Krishnan et al.[37] Similarly to the bootstrap ratio, the CVR of pattern element j, be it a clinical-neurocognitive variable, a polygenic risk score, an image voxel or the decision score of a unimodal risk calculator, was defined as:

$$\mathrm{CVR}_j = \frac{\left(\sum_{i=1}^{n=p_{CV_1}*k_{CV_1}*r_{CV_2}*k_{CV_2}} \hat{\mathbf{w}}_j^i\right)/n}{\sigma_{\hat{\mathbf{w}}_j^i}/\sqrt{n}}$$

Where n is the size of the SVM ensemble, p_{CV_1} is the number of CV_1 permutations, k_{CV_1} the number of CV_1 folds, r_{CV_2} the number of CV_2 repetitions, k_{CV_2} the number of CV_2 folds, $\hat{\mathbf{w}}_j^i$ the jth element of the ith normalized weight vector $\hat{\mathbf{w}}^i = \mathbf{w}^i/\|\mathbf{w}^i\|$ in the SVM ensemble,[38–40] $\sigma_{\hat{\mathbf{w}}_j^i}$ the standard deviation of $\hat{\mathbf{w}}_j^i$. Akin to *Z*-scores, the CVR vectors or images (see Figures in main manuscript) were thresholded at CVR=±3 to delineate stable pattern elements across the cross-validation experiment.

Furthermore, we implemented a sign-consistency-based method to statistically probe the relevance of clinical-neurocognitive variables, polygenic risk scores or image voxels in our SVM ensembles. To this end, we adopted and extended the approach proposed by Gómez-Verdejo et al.[41] toward wrapper-based feature selection strategies. The variable importance of the jth pattern element in the three unimodal risk calculators was defined as:

$$I_j = \frac{\left|\sum_{i=1}^{n} \hat{\mathbf{w}}_j^i > 0 \; - \sum_{i=1}^{n} \hat{\mathbf{w}}_j^i < 0\right|}{n} * \left(1 - \frac{\sum_{i=1}^{n} \hat{\mathbf{w}}_j^i = 0}{n}\right)$$

The first part of the equation measures the consistency of the weights assigned by the SVM ensemble to given pattern element. The importance I_j is reduced by the second part of the equation, which measures the fraction of SVMs that de-selected the given pattern element during the wrapper-based optimization process (see **section 1.5.3**). Hence, $I_j = 1$, when the weights of jth pattern element all share the same sign and the element has been selected by all classifiers in the ensemble, or $I_j = 0$, when positive and negative weights occur equally across the ensemble or given pattern element has been omitted by all classifiers in the ensemble. We defined a hypothesis test for I_j, with:

$$\begin{cases} H_0: I_j = 0, & j \text{ is not relevant} \\ H_1: I_j > 0, & j \text{ is relevant} \end{cases}$$

Significance thresholds for this hypothesis test were derived using the *Z*-statistic, defined as:

$$z_j = \frac{I_j}{\sqrt{var\{I_j\}}}$$

We used the normal cumulative distribution function to pick the right-tailed *P* value corresponding to the respective *Z*-score of the variable importance of I_j. The *P* values were corrected for multiple comparisons using the false-discovery rate[42] and statistical significance was defined at α=0.05.

1.5.6. Predictive signature validation

As in our previous work,[26] we used the HC sample to assess whether the multivariate signatures of our risk calculators (i.e., the clinical-neurocognitive variables, voxels of GMV increments/reductions or ancestry-adjusted PRS increments/reductions selected as the most predictive of PT) could be interpreted as patterns indicating a deviation from normality.

For the clinical-neurocognitive risk calculator, we drew a sample of 244 HC participants from the age-, sex- and site-matched HC cohort (**eTable 3**), for which less or equal than 25% of the 141 variables used in the training of the clinical-neurocognitive risk calculator were missing.[43] The following preprocessing steps were performed to compare the patients' data to HC: First, the data matrix of the HC participants was scaled to [0,1] and missing values were imputed using the same nearest neighbor-

© 2020 Koutsouleris N et al. *JAMA Psychiatry.*

based method as in the main risk calculator analysis. Then, the mean ($\bar{x}$) and standard deviation (σ) were computed for each of the reliably PT-predictive variables in the scaled and imputed data matrix of the HC sample (see CVR profile of clinical-neurocognitive variables in **Figure 1, A**, main manuscript). Finally, the HC-based scaling and imputation models were applied to the data of the clinical study participants. The HC-based means and standard deviations were used to standardize the scaled and imputed data of the HC, CHR, and ROD participants using the formula $z_i = (x_i - \bar{x})/\sigma(x)$, thus creating a measure of deviation from normality across these variables. Several variables (DANVA, CTQ item 13, DSST, SVF and PVF, ROCF, SOPT, WAIS) were inverted to facilitate score interpretation, i.e. a higher positive score corresponded to a higher PT-related abnormality of the given variable. The resulting abnormality profiles were visually compared between HC, predicted PT and predicted NT patients (**eFigure 7**). Latter two groups were produced by the prognostic assignments of the clinical-neurocognitive risk calculator. Then, the three groups were statistically compared using Multivariate Analysis of Variance (MANOVA) provided by the Statistical Package for the Social Sciences (SPSS v.25, IBM Inc.). The right side of **eFigure 7** shows the results of the between-group ANOVAs which were conducted following the significant omnibus test. The *P* values of these ANOVAs were corrected for multiple comparisons using the false-discovery rate. In significant ANOVAs, we performed pairwise post hoc analyses and adjusted the obtained *P* values for multiple comparisons using Tukey's Honestly Significant Differences (HSD) method.

For the PRS-based risk calculator, we conducted a similar MANOVA by defining the 10 ancestry-adjusted PRS scores for schizophrenia computed at different genome-wide significance thresholds (see **section 1.4**) as dependents in the analysis. A three-group dummy matrix consisting of the two predicted outcome groups and a subgroup of 209 healthy volunteers for whom PRS were available was entered as a fixed factor into the analysis. After assessing the omnibus test significance using Wilk's λ, univariate ANOVAs were computed for each PRS score and an alpha correction was carried out using the FDR. In significant ANOVAs, post hoc comparisons were performed to evaluate pairwise group differences. Tukey's HSD method was employed to correct the alpha level of pairwise comparisons. Additionally, we repeated the analysis using the observed transition labels and the baseline study groups to test the specificity of findings. All PRS-related MANOVA results are shown in **eFigure 8**.

For the sMRI-based risk calculator, we performed voxel-based morphometry (VBM) with threshold-free cluster enhancement (TFCE)[44] in Statistical Parametric Mapping (SPM12)[16] to compare the predicted outcome samples to our site-, age- and sex-matched sample of 331 healthy volunteers. To this end, we smoothed the study participants' GMV maps with a 6 mm Gaussian kernel, proportionally scaled them to their global GMV and masked the images with a binarized version of the CVR map shown in **Figure 1** (main manuscript) to restrict the analysis to stable parts of the sMRI-based risk calculator's signature. The smoothing kernel width of 6 mm was chosen based on the parameter combination that was most frequently selected across the sMRI-based SVM ensemble. The smoothed, scaled and masked GMV maps entered a non-parametric analysis of variance as implemented in the TFCE toolbox for SPM12 (http://www.neuro.uni-jena.de/tfce/). For each of the three comparisons [predicted PT vs. HC], [predicted NT vs. HC], and [predicted PT vs. predicted NT] a voxel-wise null distribution of TFCE values was computed using 5000 random label permutations. The resulting *P* value maps were log-transformed, corrected using the false-discovery rate and visualized in **eFigure 9**.

1.5.7. Diagnostic moderators of prognostic performance

To evaluate whether differences between the CHR and ROD groups had influenced the prognostic performance of our unimodal, stacked and cybernetic risk calculators, we first replaced the transition outcomes with the diagnostic labels in all classification experiments conducted in the complete PRONIA sample and re-trained all risk calculators with the identical algorithmic setup used for the prognostic analyses (**eTable 12**). Then, we computed a cross-correlation matrix of the prognostic and differential

© 2020 Koutsouleris N et al. *JAMA Psychiatry.*

diagnostic OOT decision scores and inspected the pairwise explained variances R^2 and corresponding *P* values (**eTable 13**). An alpha correction was carried out using FDR.

Furthermore, we evaluated whether training our risk calculators on a broader risk population, which encompassed both CHR and ROD patients, lead to a better prediction performance in the CHR sample in contrast to training and cross-validating only in the CHR population. We hypothesized that the widening of the risk spectrum due to the inclusion of the lower-risk ROD patients (1.8% transitions compared with 13.8% in the CHR group) would lead to a more accurate and generalizable prediction of the CHR patients' transition risk across all data domains. To test this hypothesis, we conducted two sets of analyses in the complete PRONIA cohort and used the identical algorithmic setup as for the main analyses. First, we removed all ROD patients from our sample, retrained and cross-validated all unimodal risk calculators and the stacked model using only the CHR patients. Then, we tested whether the reduced model performance observed in these analyses was simply caused by the reduction of training sample size. To this end, we replaced the 167 ROD patients in the complete PRONIA cohort with 167 age, sex and site-matched HC individuals drawn from our HC cohort (**eTable 3**). Again, we retrained all unimodal and stacked risk calculators and measured the prognostic performance in CHR sample using LOSOCV. The results of these ROD depletion and substitution analyses are reported in **eTable 14** alongside the original performances of the risk calculators in the CHR group. Furthermore, to statistically test our hypothesis, we performed a Quade test for each unimodal risk calculator and the stacked model. If the given omnibus test was significant at α=0.05, we conducted pairwise post hoc tests of performance differences between the original model and the 'ROD-depleted' classifier, the original model and the 'ROD-replaced' classifier, and the 'ROD-depleted' and 'ROD-replaced' models. Obtained P values were corrected for multiple comparisons using the false-discovery rate and visualized in **eFigure 12**.

1.5.8. Prognostic generalization of raters and risk calculators to outcome targets beyond PT

Similar to Koutsouleris et al. 2018,[26] we assessed whether our raters' estimates and the risk calculators' predictions of PT generalized to other prediction targets. First, we tested whether their psychosis transition (PT) estimates were sensitive to *non-remission or de-novo occurrence* of CHR syndromes, as an intermediate outcome between transition and CHR symptom remission (**eTable 15**). More specifically, we created a new three-group outcome label that differentiated between transition to psychosis (PT), non-remission/de-novo occurrence of CHR syndromes (P-CHR), and asymptomatic courses (NP-CHR). The P-CHR outcome was defined by the presence of PRONIA CHR criteria in at least one follow-up examination conducted after the 6-month follow-up interval. Consequently, NP-CHR patients had to show no CHR criteria in any of the visits after the 6-month timepoint. Then, we used a non-parametric permutation approach to test whether the raters' outcome estimates or the OOT decision scores of the risk calculators trained to differentiate between PT and NT cases were also significantly separating outcomes in the three classification tasks 'PT vs. PR', 'PT vs. NP-CHR', and 'P-CHR vs. NP-CHR'. We established significant discriminative effects by computing the null distributions of BAC using 5000 conjoint permutations of all outcome predictions and comparing the observed BAC of respective prediction models in given classification tasks to their respective null distributions. Based on the conjoint permutation approach, it was also possible to evaluate whether our binary risk prediction models were differentially associated with the three-outcome label: we computed the null distributions of BAC differences (ΔBAC) and compared the observed ΔBAC to those distributions. Alpha corrections were carried out for these two analysis steps using the FDR and significance was established at α=0.05.

Secondly, we evaluated whether the prognostic estimates of our raters and risk calculators, as well as the observed outcome classes were associated with more fine-grained CHR and functional syndrome trajectories. Similar to Koutsouleris et al. 2018,[26] these syndromes were extracted from the clinical baseline data using unsupervised machine learning methods. More specifically, we first scaled

© 2020 Koutsouleris N et al. *JAMA Psychiatry.*

the patients' psychometric baseline data, including items from the SIPS, SPI-A, and the GF:S, GF:R questionnaires, to the range [0, 1] and imputed missing data using the approach described in **section 1.5.2**. We applied orthogonal non-negative matrix factorization[46] to the scaled and imputed matrix in order to reduce it to four factor scores (**eFigure 13, A**). After sorting the features according to their factor weights, we interpreted the four factors as *paranoid-perceptual disturbances* (F1), *disturbances of volition and affect* (F2), *functional disturbances* (F3) and *cognitive disturbances* (F4). Then, scaling, imputation and factorization models were applied without further modification to the clinical data available for the T1, T2, T3 and T4 timepoints to produce follow-up scores for each of the four factors domains. Finally, a matrix of univariate linear mixed-effects models was computed to detect differences between prognostic assignments (**eFigure 13, B1**) and observed outcomes (**eFigure 13, B2**). In each of the 24 (=4 factors × 6 predictors) analyses, we defined the respective factor score as response variable and added intercept, *group* (predicted or observed) and its interaction with *timepoint* as fixed factors, while *subject* and *site*-level intercepts were entered as random effects into the model. An alpha correction of *P* values for the main and interaction effects was performed using the FDR. Statistical significance was defined at α=0.05.

Thirdly, we evaluated whether the raters' and risk calculators' transition estimates were predictive of *non-psychotic diagnostic outcomes* over the follow-up period of the study. For each of the affective, anxiety, substance dependency and eating disorders sections of the DSM-IV-TR[45] we distinguished between four possible outcomes, consisting of 'no diagnostic criteria met', 'remission from baseline diagnosis, 'non-remission from baseline diagnosis, 'de-novo occurrence of diagnosis'. These outcomes were delineated in the following order: First, 'de-novo occurrence of diagnosis' was defined as meeting DSM-IV criteria for any condition in given diagnostic section during follow-up but not at baseline. If criteria for de-novo occurrence were not fulfilled, 'non-remission from baseline diagnosis' for given diagnostic section was considered. Non-remission was defined when symptomatic criteria were met both at baseline and at least at one follow-up examination (T1, T2, T3, or T4; see **eFigure 1**) for any diagnostic entity in given section. Finally, if these two outcomes were not observed, remission was considered, defined as criteria for any disease in given diagnostic domain being met at baseline, but not anymore during any follow-up timepoint. Major depressive disorder was treated as a separate domain because of its high prevalence in the study population. In addition, we analyzed whether the observed transition vs. non-transition outcomes were associated with these non-psychotic outcomes. **eTable 16** summarizes the results of these χ^2 analyses across diagnostic domains (rows) and transition predictors (columns). An alpha correction for multiple comparisons was carried out using the FDR and statistical significance was determined at α=0.05.

1.5.9. Permutation analysis

Given the relatively low number of transition cases in the PRONIA database, we tested the hypothesis that the SVM-based risk calculators had learned prediction rules that accidentally generalized within our sample. We, therefore, tested the statistical significance of our risk calculators' prediction performance by performing label permutation analyses.[22] More specifically, we used LOSOCV (as stated in section 1.5.1) to retrain and cross-validate all unimodal, stacked, cybernetic and sequentially stacked risk calculators on the *entire* PRONIA sample in order to maximize the available sample size. Then, we constructed a permutation structure consisting of $n = 1000$ random transition label shuffles and used 1000 permuted LOSOCV experiments to probe our models. Specifically, at each label permutation, we retrained the SVM ensembles using the same cross-validation setup and respective feature sets as in the original label analyses. Hence, we accumulated the random models' predictions into a permuted ensemble prediction for each CV_2 patient. This procedure generated a null distribution of OOT classification performance for each risk calculator and this distribution was used to compute the significance of the observed performance, as:

© 2020 Koutsouleris N et al. *JAMA Psychiatry.*

$$P_{\mathrm{OOT}_{\mathrm{observed}}} = \frac{\sum_{i=1}^{n} \begin{cases} 1 \text{ if } \mathrm{BAC}^{i}_{\mathrm{OOT}_{\mathrm{permuted}}} \geq \mathrm{BAC}_{\mathrm{OOT}_{\mathrm{observed}}} \\ 0 \text{ if } \mathrm{BAC}^{i}_{\mathrm{OOT}_{\mathrm{permuted}}} < \mathrm{BAC}_{\mathrm{OOT}_{\mathrm{observed}}} \end{cases}}{n}$$

The obtained *P* values were corrected for multiple comparisons using the FDR. The corrected significance threshold was defined at α=0.05. Corrected P values were reported in **Table 2** of the main manuscript.

1.5.10. External validation of risk calculators

We validated an abbreviated, pragmatic version of our clinical-neurocognitive risk calculator by first extracting the 7 features that were significant in the sign-based consistency visualization analysis (see **1.5.5, Figure 1**, main manuscript, and **eFigure 2**) from the original space of 142 features. We retrained the SVM ensemble using these features and the identical training parameters as in the original analysis, except for the wrapper-based feature selection, which was skipped because of our feature pre-selection selection strategy. Then, we tested this condensed risk calculator in the independent cohort of 88 PRONIA non-transition cases to probe our model's specificity. After completing this procedure, we retrained the condensed risk calculator using the entire PRONIA dataset and applied it to two independent patient samples extracted from the ZInEP[23] and BEARS-Kid[47] cohorts (**eFigure 2**). The ZInEP (*Zürcher Impulsprogramm zur Nachhaltigen Entwicklung der Psychiatrie*) study was a prospective naturalistic study of patients with CHR states, aged 13 to 35, who were similarly recruited as in PRONIA using the SIPS[3] and SPI-A/-CY[4,48] instruments. Patients were enrolled in the Zurich area, Switzerland, and examined following a multi-level approach which included psychopathological, neuropsychological, and magnetic resonance imaging measures. Patients were followed every 6 months for a maximum follow-up duration of 36 months. In contrast, the BEARS-Kid project (*Bi-national Evaluation of At-Risk Symptoms in Children and Adolescents*) was a study of CHR symptoms in individuals aged 8 to 40 who were sampled from the 384,000 persons included in the population registry of Canton Bern, Switzerland. Study participants were followed over a period of 24 months and assessed with the SIPS and SPI-A/-CY amongst others clinical instruments. For the purpose of externally validating the clinical-neurocognitive risk calculator, we extracted a sample of 462 patients with various mental conditions from the full BEARS-Kid cohort (see **eTable 19**). Importantly, the transition rate in this sample measured 2.8% and hence was significantly lower compared to the CHR sample (11.0%) of the ZInEP study (*df*=2, χ^2=16.2, *P*<.001). This difference allowed us to test the generalizability of the clinical-neurocognitive risk calculator at two distinct pre-test risk enrichment levels, which could occur in a general youth mental health service vs. a service specifically targeting patients at risk of psychotic disorders.[49] To qualitatively compare these two cohorts to the PRONIA sample, we selected sociodemographic and clinical variables that closely matched the variables analyzed in **Table 1**, main manuscript, and performed group-level comparisons of transition vs. non-transition cases, which were reported in **eTable 18** (ZInEP) and **eTable 19** (BEARS-Kid). Alpha corrections were carried out as in the PRONIA group-level analyses using the FDR and statistical significance was defined at α=0.05. In both samples all five SIPS variables needed for the application of the risk calculator were available, but not the CTQ item ('People in my family looked for each other') and the DANVA item ('No. of correctly identified facial expressions'). Prior to the application of the SVM ensemble, these missing variables were imputed by the trained data pre-processing module of the clinical-neurocognitive risk calculator (see **1.5.2**).

Furthermore, we tested the external validity of our sMRI-based risk calculator using the imaging data provided by ZInEP (n=146) and the Fe*Psy*[24] (n=37) studies. The Fe*Psy* cohort analyzed in the present work is identical to the one previously described in Koutsouleris et al[50]. The transition rate in this sample measured 43.2% and thus was significantly higher (df=2, χ^2=21.3, *P*<.001) than among the ZInEP patients (11.0%). The structural images of these two samples were pre-processed using the same CAT12 toolbox running with the parameters employed for the pre-processing of the PRONIA images

© 2020 Koutsouleris N et al. *JAMA Psychiatry.*

(see **1.5.2**). No specific calibration of the pre-processed images to the PRONIA data was performed. Then, the sMRI-based SVM ensemble retrained on the entire PRONIA cohort was used to generate outcome predictions for both external samples. We report the OOCV performance of the classifier in **Table 2**, main manuscript.

Because both psychopathological and imaging data were available for the 146 patients of the ZInEP cohort, we were able to test the external validity of a reduced stacked risk calculator that was trained on the two unimodal risk calculators described above. Thus, we were able to evaluate whether a multi-modal prognostic system outperformed its constituent unimodal components in independent and external datasets. First, we trained the stacked prognostic system on the PRONIA patients with 18-months follow-up data and then validated the system using the 88 PRONIA non-transition cases using the same preprocessing and machine learning settings employed for the training and cross-validation of the fully stacked or cybernetic risk calculators. Then, we performed the external validation of the stacked risk calculator by retraining it on the entire PRONIA cohort and applying it to the predictions generated by the unimodal clinical and sMRI-based risk calculators in the ZInEP sample. Finally, we compared the predictive performance of the clinical-neurocognitive, sMRI-based, and stacked models in the ZInEP data using Quade test (**Figure 2**, main manuscript).[51]

© 2020 Koutsouleris N et al. *JAMA Psychiatry.*

eTable 1. Study Inclusion/Exclusion Criteria of the Study

<table>
<tr><th>Group Inclusion Criteria</th><th>Group Exclusion Criteria</th><th>General Inclusion / Exclusion / Drop-out</th></tr>
<tr><td>Psychosis-risk syndrome defined:

EITHER by Attenuated Positive Symptoms (APS), as measured by the SIPS (requires 1 of 5 attenuated psychotic symptoms: unusual thought content/ delusional ideas, suspicious-ness/persecutory ideas, grandiosity, perceptual abnormalities/hallucinations, and disorganized communication) with a moderate to severe, but not psychotic, severity (SIPS score 3-5) that (1) began with-in the past year or was rated one or more scale points higher compared to 12 month ago, AND (2) occurred at an average frequency of at least once per week for at least several minutes per event in the past month</td>
<td rowspan="4">1. Any intake of antipsychotic medication for more than 30 cumulative days at or above the minimum dosage threshold defined by the DGPPN S3 Guidelines for the treatment of first-episode psychosis (eTable 2 and [29]),
2. Any intake of antipsychotic drugs within the past 3 months before psychopathological baseline assessments at or above the minimum dosage threshold.
3. Occurrence of the CHR syndrome is better explained by other DSM-IV disorder</td>
<td rowspan="5">Inclusion Criteria:
1. Age 15 to 40 years
2. Language skills sufficient for participation
3. Able to provide to consent / assent

Exclusion Criteria:
1. IQ below 70
2. Hearing is not sufficient for neuro-cognitive testing
3. Current or past head trauma with loss of consciousness (> 5 min)
4. Current or past known neurological disorder of the brain
5. Current or past known somatic disorder potentially affecting the structure or functioning of the brain
6. Current or past alcohol dependence
7. Current poly-substance dependence or within the past six months (Note: any combination with E.6. led to exclusion)
8. Any contra-indication for MRI

Exclusion criteria for healthy controls:
1. Any current or past DSM-IV axis disorder
2. A positive familial history (1st degree relatives) for affective or non-affective psychoses or major affective disorders; and
3. An intake of psychotropic medications or drugs more than 5 times/year and in the month before study inclusion.

Drop-out criteria:
1. No follow-up examination after the 6-months follow-up examination (IV6)
2. Withdrawn consent / assent</td></tr>
<tr><td>OR: by Brief Intermittent Psychotic Symptoms (BLIPS), as measured by the SIPS (as defined by one of the symptoms listed above (1) reaching a psychotic level of intensity in each of the past 3 months for at least several minutes per day, OR (2) reaching a psychotic level of intensity in the past month, occurring at an average frequency of at least once per week for at least several minutes per event in the past month, or occurring at least for a cumulative period of more than one hour within the past month, AND (1+2) remitting spontaneously within one week (i.e. without antipsychotic medication)</td></tr>
<tr><td>OR: by a Genetic Risk and Functional Decline Psychosis-Risk Syndrome (GRFD) defined by a current 30% or greater reduction in the functional disability score of the split version of the Global Assessment of Functioning Scale (GAF-F) compared with the highest lifetime level of functioning, AND (having a first-degree relative with a history of any psychotic disorder, OR having a DSM-IV-TR schizotypal personality disorder).</td></tr>
<tr><td>OR: by a Cognitive Disturbance Syndrome (COGDIS) as measured by the SPI-A (requires at least 2 of 9 cognitive basic symptoms with at least weekly occurrence (score ≥3) during the last 3 months)</td></tr>
<tr><td>Recent-onset Depression as defined by DSM-IV-TR major depression category + ALL of the following criteria:
1. First life-time depressive episode,
2. Duration of current depressive episode no longer than 24 months,
3. Diagnostic criteria fulfilled within past three months</td>
<td>1. Occurrence of the major depressive episode is better explained by other DSM-IV disorder
2. See CHR exclusion criteria</td></tr>
</table>

© 2020 Koutsouleris N et al. *JAMA Psychiatry.*

eTable 2. Antipsychotic Medication Thresholds Based on the Previous Version of the S3 Guidelines of the German Association for Psychiatry, Psychotherapy and Psychosomatics

Candidate CHR and ROD patients were excluded if they had received antipsychotic medication (1) for more than 30 cumulative days at or above the minimum target dosage threshold for the treatment of first-episode psychosis, or (2) within the past 3 months before psychopathological baseline assessments at or above the minimum target dosage threshold for the treatment of first-episode psychosis.

**Substance**	**Recommended starting dosage (mg/d)**	**DI'**	**Target dosage first-episode psychosis (mg/d)**	**Target dosage relapsing schizophrenia (mg/d)**	**Maximum dosage recommended (mg/d)[2]**
**Atypical Antipsychotics**					
Amisulpride	200	(1)-2	100-300	400-800	1200
Aripiprazole	(10)-15	1	15-(30)	15-30	30
Olanzapine	5-10	1	5-15	5-20	20
Quetiapine	50	2	300-600	400-750	750
Risperidone	2	1-2	1-4	3-6-(10)	16
Ziprasidone	40	2	40-80	80-160	160
**Typical Antipsychotics**					
Fluphenazine	0.4-10	2-3	2.4-10	10-20	20-(40)
Flupentixole	2-10	1-3	2-10	10-60	60
Haloperidole	1-10	(1)-2	1-4	3-15	100
Perazine	50-150	1-2	100-300	200-600	1000
Perphenazine	4-24	1-3	6-36	12-42	56
Pimozide	1-4	2	1-4	2-12	16
Zotepine	25-50	2-(4)	50-150	75-150	450
Zuclopenthixole	2-50	1-3	2-10	25-50	75

Abbreviations: DI dosage interval, [2]maximum recommended dosage according to prescribing information.

© 2020 Koutsouleris N et al. *JAMA Psychiatry.*

eTable 3. Group-Level Comparison of CHR, ROD and HC Individuals

Groups are compared across sociodemographic, functioning, psychopathological and selected neurocognitive domains. *P* values were corrected for multiple comparisons using the False-Discovery Rate (P_{FDR}). Significant comparisons in bold.

Variables	CHR	ROD	HC	*df*	$T/Z/\chi^2$	P_{FDR}†	$P_{HC\ vs.\ CHR}$	$P_{HC\ vs.\ ROD}$	$P_{CHR\ vs.\ ROD}$
**Sociodemographic variables**									
Sample sizes	167	167	334						
Munich	53	56	109	12	10.84	.54			
Milan	15	10	25						
Basel	19	15	34						
Cologne	23	40	63				—	—	—
Birmingham	17	19	36						
Turku	25	12	37						
Udine	15	15	30						
Age [mean (SD) years]	23.9 (5.4)	25.7 (6.1)	25.4 (5.7)	2	**5.32**	**.007**	**.01**	1.00	**.01**
Sex [F (%)]	83 (49.7)	86 (51.5)	185 (55.4%)	2	1.75	.44	—	—	—
Ethnicity									
Caucasian	143	149	286	8	9.47	.33			
Asian	15	9	28						
African	3	1	3				—	—	—
Mixed	3	2	12						
Other	3	6	4						
Body-Mass Index [mean (SD) m2/kg]	23.3 (4.2)	24.1 (5.0)	22.9 (3.6)	2	**4.49**	**.02**	.98	**.009**	.24
Edinburgh Handedness Score [mean (SD)]	63.6 (59.8)	72.5 (47.3)	76.6 (43.4)	2	**3.37**	**.04**	**.03**	1.00	.35
Education [mean (SD) years]	13.6 (2.8)	14.9 (2.9)	15.8 (3.2)	2	**30.57**	**<.001**	**<.001**	**.003**	**<.001**
Educational problems [mean (SD) years repeated]	0.43 (1.17)	0.38 (1.29)	0.13 (0.38)	2	**7.76**	**<.001**	**.002**	**.010**	1.00
Having a partnership most of the time in the year before study inclusion [Yes (%)]	77 (47.0)	91 (54.5)	222 (67.9)	2	21.95	**<.001**	**<.001**	**.009**	.31
**Functioning [mean (SD)]**									
GF:S Highest Lifetime	7.86 (0.84)	8.07 (0.89)	8.71 (0.68)	2	133.76	**<.001**	**<.001**	**<.001**	.15
GF:S Baseline	6.31 (1.45)	6.50 (1.31)	8.47 (0.78)	2	344.09	**<.001**	**<.001**	**<.001**	.42
GF:R Highest Lifetime	7.95 (0.80)	8.26 (0.83)	8.67 (0.68)	2	91.25	**<.001**	**<.001**	**<.001**	**.002**
GF:R Baseline	6.01 (1.60)	6.13 (1.76)	8.52 (0.77)	2	365.97	**<.001**	**<.001**	**<.001**	1.00
**High-risk symptoms [mean (SD)]**									
SIPS Positive Symptoms	1.60 (0.90)	0.43 (0.44)	0.10 (0.21)	2	**417.92**	**<.001**	**<.001**	**<.001**	**<.001**
SIPS Negative Symptoms	1.64 (1.17)	1.51 (0.97)	0.03 (0.09)	2	**292.75**	**<.001**	**<.001**	**<.001**	.43
SIPS Disorganized Symptoms	0.81 (0.72)	0.58 (0.59)	0.02 (0.07)	2	**158.24**	**<.001**	**<.001**	**<.001**	**<.001**
SIPS General Psychopathology	1.89 (1.00)	1.90 (0.95)	0.06 (0.17)	2	**492.01**	**<.001**	**<.001**	**<.001**	1.00
Mean of 9 COGDIS symptoms	0.83 (0.67)	0.24 (0.32)	0.02 (0.08)	2	**214.75**	**<.001**	**<.001**	**<.001**	**<.001**
**Neurocognitive variables [mean (SD)]**									

© 2020 Koutsouleris N et al. *JAMA Psychiatry.*

DANVA (No. of correctly identified facial expressions)	18.9 (2.3)	19.2 (2.3)	19.3 (2.3)	2	1.59	.24	—	—	—
DSST (No. of correct symbol matches)	65.2 (10.5)	61.6 (11.7)	59.1 (12.2)	2	**15.5**	**<.001**	**<.001**	**.004**	.15
PVF (No. of correct words from a phonetic category in 60 sec)	14.5 (5.1)	14.2 (4.7)	15.6 (5.0)	2	**5.06**	**.01**	.07	**.01**	1.00
SVF (No. of correct words from a semantic category in 60 sec)	22.8 (5.9)	24.0 (6.6)	25.6 (6.0)	2	**10.6**	**<.001**	**<.001**	**.03**	.24

© 2020 Koutsouleris N et al. *JAMA Psychiatry.*

eTable 4. Diagnostic Breakdown of Psychotic Disorders in PRONIA Cases With a Disease Transition

DSM-IV-based diagnostic breakdown of psychotic disorders in CHR and ROD patients who developed a disease transition during the follow-up period. Diagnoses were examined at the 9-months and 18-months timepoints using the Structured Clinical Interview for DSM-IV-TR.[52]

**Diagnoses**	**All**	**CHR**	**ROD**
N	26	23	3
Transition rate [%]	7.8	13.8	1.8
Schizophrenia	8	7	1
Schizophreniform disorder	4	4	0
Schizoaffective disorder	2	2	0
Delusional disorder	1	1	0
Brief psychotic disorder	2	1	1
Psychosis NOS*	5	5	0
Bipolar disorder type I, manic episode with mood-congruent psychotic features	1	1	0
Major depression with mood incongruent psychotic features	1	0	1
Major depression with mood-congruent psychotic features	2	2	0

*excluding brief limited intermittent psychotic symptoms.

© 2020 Koutsouleris N et al. *JAMA Psychiatry.*

eTable 5. Sociodemographic, Clinical and Neurocognitive Variables Used in the Clinical Prediction Models

Variable ID	Variable Description	Instrument
Age		
Sex		
SIPS P1	Unusual thought content/delusional ideas	Structured Interview for Psychosis-Risk Syndromes
SIPS P2	Suspisciousness/persecutory ideas	Structured Interview for Psychosis-Risk Syndromes
SIPS P3	Grandiose ideas	Structured Interview for Psychosis-Risk Syndromes
SIPS P4	Perceptual abnormalities/hallucinations	Structured Interview for Psychosis-Risk Syndromes
SIPS P5	Disorganized communication	Structured Interview for Psychosis-Risk Syndromes
SIPS N1	Social anhedonia	Structured Interview for Psychosis-Risk Syndromes
SIPS N2	Avolition	Structured Interview for Psychosis-Risk Syndromes
SIPS N3	Expression of emotions	Structured Interview for Psychosis-Risk Syndromes
SIPS N4	Experience of emotions and self	Structured Interview for Psychosis-Risk Syndromes
SIPS N5	Ideational richness	Structured Interview for Psychosis-Risk Syndromes
SIPS N6	Occupational functioning	Structured Interview for Psychosis-Risk Syndromes
SIPS D1	Odd behaviour or appearance	Structured Interview for Psychosis-Risk Syndromes
SIPS D2	Bizzarre thinking	Structured Interview for Psychosis-Risk Syndromes
SIPS D3	Trouble with focus and attention	Structured Interview for Psychosis-Risk Syndromes
SIPS D4	Impairment in personal hygiene and social attentiveness	Structured Interview for Psychosis-Risk Syndromes
SIPS G1	Sleep disturbance	Structured Interview for Psychosis-Risk Syndromes
SIPS G2	Disphoric mood	Structured Interview for Psychosis-Risk Syndromes
SIPS G3	Motor disturbances	Structured Interview for Psychosis-Risk Syndromes
SIPS G4	Impaired tolerance to normal stress	Structured Interview for Psychosis-Risk Syndromes
SPI-A B1	Inability to divide attention	Schizophrenia Proneness Instrument
SPI-A C2	Thought interference	Schizophrenia Proneness Instrument
SPI-A C3	Thought blockages	Schizophrenia Proneness Instrument
SPI-A C4	Disturbance of receptive speech	Schizophrenia Proneness Instrument
SPI-A C5	Disturbance of expressive speech	Schizophrenia Proneness Instrument
SPI-A D3	Thought pressure	Schizophrenia Proneness Instrument
SPI-A D4	Unstable ideas of reference, "subject-centrism"	Schizophrenia Proneness Instrument
SPI-A O1	Thought perseveration	Schizophrenia Proneness Instrument
SPI-A O2	Decreased ability to discriminate between ideas and perception, fantasy and true memories	Schizophrenia Proneness Instrument
SPI-A O3	Disturbances of abstract thinking	Schizophrenia Proneness Instrument
SPI-A O4 00	Other visual perception disturbances	Schizophrenia Proneness Instrument
SPI-A O4.01	Near and television	Schizophrenia Proneness Instrument
SPI-A O4.02	Metamorphopsia	Schizophrenia Proneness Instrument
SPI-A O4.03	Changes in colour vision	Schizophrenia Proneness Instrument
SPI-A O4.04	Changed perception of the patient ́s own face	Schizophrenia Proneness Instrument
SPI-A O4.05	Pseudomovements of optic stimuli	Schizophrenia Proneness Instrument
SPI-A O4.06	Diplopsia, oblique vision	Schizophrenia Proneness Instrument
SPI-A O4.07	Disturbances of the estimation of distances or size	Schizophrenia Proneness Instrument
SPI-A O4.08	Disturbances of the perception of straight lines or contours	Schizophrenia Proneness Instrument

© 2020 Koutsouleris N et al. *JAMA Psychiatry.*

SPI-A O4.09	Maintenance of visual stimuli, "visual echoes"	Schizophrenia Proneness Instrument
SPI-A O4.10	Partial seeing including tubular vision	Schizophrenia Proneness Instrument
SPI-A D5	Changed perception to the face or body of others	Schizophrenia Proneness Instrument
SPI-A F2	Photopsia	Schizophrenia Proneness Instrument
SPI-A F3	Micropsia, macropsia	Schizophrenia Proneness Instrument
SPI-A O5	Other acoustic perception disturbances	Schizophrenia Proneness Instrument
SPI-A O5.1	Acoasms	Schizophrenia Proneness Instrument
SPI-A O5.2	Maintenance of acoustic stimuli, "acoustic echoes"	Schizophrenia Proneness Instrument
SPI-A F5	Changes in the perceived intensity or quality of acoustic stimuli	Schizophrenia Proneness Instrument
SPI-A O7	Captivation of attention by details of the visual field	Schizophrenia Proneness Instrument
SPI-A O8	Derealization	Schizophrenia Proneness Instrument
GF S Current	Current functioning score	Global Functioning: Social Scale (GF-S)
GF S LowPastYearT0	Lowest functioning score in the past year	Global Functioning: Social Scale (GF-S)
GF S HighPastYearT0	Highest functioning score in the past year	Global Functioning: Social Scale (GF-S)
GF S HighLifetimeT0	Highest lifetime functioning score	Global Functioning: Social Scale (GF-S)
GF R Current	Current functioning score	Global Functioning: Role Scale (GF-R)
GF R LowPastYearT0	Lowest functioning score in the past year	Global Functioning: Role Scale (GF-R)
GF R HighPastYearT0	Highest functioning score in the past year	Global Functioning: Role Scale (GF-R)
GF R HighLifetimeT0	Highest lifetime functioning score	Global Functioning: Role Scale (GF-R)
GAF S LifeTime	Highest lifetime score	Global Assessment of Functioning - Symptoms
GAF S PastYearT0	Highest score in the past year	Global Assessment of Functioning - Symptoms
GAF S PastMonth	Highest score in the past month	Global Assessment of Functioning - Symptoms
GAF DI LifeTime	Highest score lifetime	Global Assessment of Functioning - Disability
GAF DI PastYear	Highest score in the past year	Global Assessment of Functioning - Disability
GAF DI PastMonth	Highest score in the past month	Global Assessment of Functioning - Disability
DANVA Number of correct faces conditions	Number of faces correctly recognized	Diagnostic analysis of nonverbal accuracy second version
DSST Score symbol matchings	Correctly matched digits to symbols	Digit-symbol substitution test
SVF Correct 00 60 category	Correct words from a semantic category in 60 seconds	Verbal fluency semantic
SVF Error 00 60 category	Errors in 60 seconds	Verbal fluency semantic
SVF Repetition 00 60 category	Repetitions in 60 seconds	Verbal fluency semantic
PVF Correct 00 60 letter	Correct words from a phonetic rule in 60 seconds	Verbal fluency phonetic
PVF Error 00 60 letter	Errors in 60 seconds	Verbal fluency phonetic
PVF Repetition 00 60 letter	Repetitions in 60 seconds	Verbal fluency phonetic
ROCF Accuracy whole	Accuracy: sum score of all elements -phase 1 (drawing from figure)	Rey-Osterrieth Complex Figure
ROCF Accuracy whole Immediate	Accuracy: sum score of all elements -phase 2 (drawing from memory immediately after copying)	Rey-Osterrieth Complex Figure
ROCF Accuracy whole Delayed	Accuracy sum score of all elements -phase 3 (drawing from memory 30 minutes after copying)	Rey-Osterrieth Complex Figure three phases: (1) drawing from the picture, (2) drawing from memory (immediate), (3) drawing from memory (delayed)
ROCF Placeme whole	Placement: sum score of all elements -phase 1 (drawing from figure)	Rey-Osterrieth Complex Figure
ROCF Placeme whole Immediate	Placement: sum score of all elements -phase 2 (drawing from memory immediately after copying)	Rey-Osterrieth Complex Figure
ROCF Placeme whole Delayed	Placement: sum score of all elements -phase 3 (drawing from memory 30 minutes after copying)	Rey-Osterrieth Complex Figure
ROCF Score whole	Accuracy and placement: sum score of all elements -phase 1 (drawing from figure)	Rey-Osterrieth Complex Figure
ROCF Score whole Immediate	Accuracy and placement: sum score of all elements -phase 2 (drawing from memory immediately after copying)	Rey-Osterrieth Complex Figure
ROCF Score whole Delayed	Accuracy and placement: sum score of all elements -phase 3 (drawing from memory 30 minutes after copying)	Rey-Osterrieth Complex Figure
ROCF Time	Time of execution: phase 1 (drawing from figure)	Rey-Osterrieth Complex Figure
ROCF Time Immediate	Time of execution: phase 2 (drawing from memory immediately after copying)	Rey-Osterrieth Complex Figure
ROCF Time Delayed	time of execution -phase 3 (drawing from memory 30 minutes after copying)	Rey-Osterrieth Complex Figure
WAIS V Raw score	Vocabulary test -raw score	Wechsler Adult Intelligence Scale
WAIS V Standard score	Vocabulary test -standard score	Wechsler Adult Intelligence Scale

© 2020 Koutsouleris N et al. *JAMA Psychiatry.*

WAIS MR Raw score	Matrices test - raw score	Wechsler Adult Intelligence Scale
WAIS MR Standard score	Matrices test - standard score	Wechsler Adult Intelligence Scale
FDS Number of correct trials	number of correctly remembered digit strings	Auditory digit span forward
FDS Maximum digits string length correctly reminded at least once	Maximum number of correctly remembered digit strings	Auditory digit span forward
BDS Number of correct trials	Number of correctly remembered digit strings	Auditory digit span backward
BDS Maximum digits string length correctly reminded at least once	Maximum number of correctly remembered digit strings	Auditory digit span backward
TMA 1 Time of execution	Time of execution	Trail-Making Test-A
TMA 2 Errors	Number of errors	Trail-Making Test-A
TMA 3 Violations	Number of rule violations	Trail-Making Test-A
TMB 1 Time of execution	Time of execution	Trail-Making Test-B
TMB 2 Errors	Number of errors	Trail-Making Test-B
TMB 3 Violations	Rule violations	Trail-Making Test-B
Diff TMB TMA	Difference in performance between TMT-A and -B	Trail-Making Test-A-B
Ratio TMB TMA	Ratio of the performance in TMT-A und -B	Trail-Making Test-A-B
SOPT Max corr responses before error 4 elemes 01	Maximum correct responses before error by 4 elements - trial 1	Self-ordered pointing task
SOPT Max corr responses before error 4 elemes 02	Maximum correct responses before error by 4 elements - trial 2	Self-ordered pointing task
SOPT Max corr responses before error 4 elemes 03	Maximum correct responses before error by 4 elements - trial 3	Self-ordered pointing task
SOPT Max corr responses before error 6 elemes 01	Maximum correct responses before error by 6 elements - trial 1	Self-ordered pointing task
SOPT Max corr responses before error 6 elemes 02	Maximum correct responses before error by 6 elements - trial 2	Self-ordered pointing task
SOPT Max corr responses before error 6 elemes 03	Maximum correct responses before error by 6 elements - trial 3	Self-ordered pointing task
SOPT Max corr responses before error 8 elemes 01	Maximum correct responses before error by 8 elements - trial 1	Self-ordered pointing task
SOPT Max corr responses before error 8 elemes 02	Maximum correct responses before error by 8 elements - trial 2	Self-ordered pointing task
SOPT Max corr responses before error 8 elemes 03	Maximum correct responses before error by 8 elements - trial 3	Self-ordered pointing task
SOPT Max corr responses before error 10 elemes 01	Maximum correct responses before error by 10 elements - trial 1	Self-ordered pointing task
SOPT Max corr responses before error 10 elemes 02	Maximum correct responses before error by 10 elements - trial 2	Self-ordered pointing task
SOPT Max corr responses before error 10 elemes 03	Maximum correct responses before error by 10 elements - trial 3	Self-ordered pointing task
CTQ 01	I didn't have enough to eat.	Childhood Trauma Questionnaire
CTQ 02	I knew that there was someone to take care of me and protect me.	Childhood Trauma Questionnaire
CTQ 03	People in my family called me things like ''stupid'', ''lazy'' or ''ugly''	Childhood Trauma Questionnaire
CTQ 04	My parents were too drunk or high to take care of the family.	Childhood Trauma Questionnaire
CTQ 05	There was someone in my family who helped me feel that I was important or special.	Childhood Trauma Questionnaire
CTQ 06	I had to wear dirty clothes.	Childhood Trauma Questionnaire
CTQ 07	I felt loved.	Childhood Trauma Questionnaire
CTQ 08	I thought that my parents wished I hadn''t been born.	Childhood Trauma Questionnaire
CTQ 09	I got hit so hard by someone in my family that I had to see a doctor or go to the hospital.	Childhood Trauma Questionnaire
CTQ 10	There was nothing I wanted to change about my family.	Childhood Trauma Questionnaire
CTQ 11	People in my family hit me so hard that it left me with bruises or marks.	Childhood Trauma Questionnaire
CTQ 12	I was punished with a belt, a board, a cord, or some other hard object.	Childhood Trauma Questionnaire
CTQ 13	People in my family looked out for each other.	Childhood Trauma Questionnaire
CTQ 14	People in my family said hurtful or insulting things to me.	Childhood Trauma Questionnaire
CTQ 15	I believe that I was physically abused.	Childhood Trauma Questionnaire

© 2020 Koutsouleris N et al. *JAMA Psychiatry.*

CTQ 16	I had the perfect childhood.	Childhood Trauma Questionnaire
CTQ 17	I got hit or beaten so badly that it was noticed y someone like a teacher, neighbour, or doctor.	Childhood Trauma Questionnaire
CTQ 18	I felt that someone in my family hated me.	Childhood Trauma Questionnaire
CTQ 19	People in my family felt close to each other.	Childhood Trauma Questionnaire
CTQ 20	Someone tried to touch me in a sexual way. Or tried to make me touch them.	Childhood Trauma Questionnaire
CTQ 21	Someone threatened to hurt me or tell lies about me unless I did something sexual with them.	Childhood Trauma Questionnaire
CTQ 22	I had the best family in the world.	Childhood Trauma Questionnaire
CTQ 23	Someone tried to make me do sexual things or watch sexual things.	Childhood Trauma Questionnaire
CTQ 24	Someone molested me.	Childhood Trauma Questionnaire
CTQ 25	I believe that I was emotionally abused.	Childhood Trauma Questionnaire
CTQ 26	There was someone to take me to the doctor if I needed it.	Childhood Trauma Questionnaire
CTQ 27	I believe that I was sexually abused.	Childhood Trauma Questionnaire
CTQ 28	My family was a source of strength and support.	Childhood Trauma Questionnaire

eTable 6. MR Scanner Systems and Structural MRI Sequence Parameters Used at the Respective PRONIA Sites

PRONIA Site	Model	Field Strength	Coil Channels	Flip Angle	TR [ms]	TE [ms]	Voxel Size [mm]	FOV	Slice Number
Munich	Philips Ingenia	3T	32	8	9.5	5.5	0.97 x 0.97 x 1.0	250 x 250	190
Milan Niguarda	Philips Achieva Intera	1.5T	8	12	Shortest (8.1)	Shortest (3.7)	0.93 x 0.93 x 1.0	240 x 240	170
Basel	SIEMENS Verio	3T	12	8	2000	3.4	1.0 x 1.0 x 1.0	256 x 256	176
Cologne	Philips Achieva	3T	8	8	9.5	5.5	0.97 x 0.97 x 1.0	250 x 250	190
Birmingham	Philips Achieva	3T	32	8	8.4	3.8	1.0 x 1.0 x 1.0	288 x 288	175
Turku	Philips Ingenuity	3T	32	7	8.1	3.7	1.0 x 1.0 x 1.0	256 x 256	176
Udine	Philips Achieva	3T	8	12	Shortest (8.1)	Shortest (3.7)	0.93 x 0.93 x 1.0	240 x 240	170

© 2020 Koutsouleris N et al. *JAMA Psychiatry.*

eTable 7. Effects of Baseline Treatments on the Decision Scores Generated by 5 Different Risk Calculators

P values were adjusted for multiple comparisons using the False-Discovery Rate (P_{FDR}).

	Not treated with antipsychotics		Treated with antipsychotics				
**Predictor**	**N**	**Mean (SD) decision score**	**N**	**Mean (SD) decision score**	***df***	**T**	P_{FDR}
Clin-NC	268	-0.135 (0.844)	63	0.157 (1.059)	332	-2.35	.29
PRS-based	236	-0.005 (0.923)	60	-0.151 (1.188)	294	0.07	.99
sMRI-based	264	-0.037 (0.466)	63	0.552 (0.476)	325	-1.41	.40
Stacked	268	-0.703 (0.851)	63	-0.491 (0.933)	329	1.75	.31
Cybernetic	268	-1.074 (1.190)	63	-0.764 (1.319)	329	-1.82	.31
	**Not treated with antidepressants**		**Treated with antidepressants**		***df***	**T**	P_{FDR}
Clin-NC	143	-0.083 (0.840)	188	-0.076 (0.936)	329	-0.07	.99
PRS-based	128	-0.030 (0.891)	168	0.011 (1.046)	294	-0.35	.99
sMRI-based	141	-0.055 (0.444)	186	0.008 (0.486)	325	-1.19	.44
Stacked	143	-0.698 (0.797)	188	-0.636 (0.921)	329	0.65	.86
Cybernetic	143	-1.016 (1.149)	188	-1.015 (1.274)	329	-0.01	.99
	**Not hospitalized before or at baseline**		**Hospitalized before or at baseline**		***df***	**T**	P_{FDR}
Clin-NC	173	0.004 (0.949)	158	-0.170 (0.824)	329	1.77	.31
PRS-based	148	-0.007 (0.885)	148	-0.006 (1.070)	294	-0.02	.99
sMRI-based	171	-0.025 (0.508)	156	-0.012 (0.422)	325	-0.24	.99
Stacked	173	-0.596 (0.884)	158	-0.736 (0.850)	329	-1.47	.40
Cybernetic	173	-0.937 (1.194)	158	-1.101 (1.244)	329	1.23	.44

Abbreviations: *Clin-NC* clinical-neurocognitive risk-calculator

© 2020 Koutsouleris N et al. *JAMA Psychiatry.*

eTable 8. Correlations Between Decision Scores of Unimodal and Cybernetic Risk Calculators and Patients' Maximum Follow-up Intervals and Number of Follow-up Examinations

Correlations strengths were measured using Spearman's rho (ρ). P values were corrected for multiple comparisons using the False-Discovery Rate (P_{FDR}). See also **eFigure 11** for a visual analysis relating the number of follow-up examinations and the longest follow-up duration to the predictive performance of these four different risk calculators.

**Variables**		**Clin-NC**	**PRS-based**	**sMRI-based**	**Stacked**	**Cybernetic**
Maximum follow-up interval	Spearman's ρ	-0.083	-0.092	-0.028	-0.077	-0.081
	P_{FDR}	0.21	0.21	0.61	0.21	0.21
Number of follow-up examinations	Spearman's ρ	-0.071	-0.083	-0.022	-0.084	-0.129
	P_{FDR}	0.27	0.26	0.70	0.26	0.10

© 2020 Koutsouleris N et al. *JAMA Psychiatry.*

eTable 9. Analysis of the Site-Related Variation in Follow-up Duration, Time to Transition, Age and Sex Distributions in the PRONIA Cohort

One-way analyses of variance (F) were carried out for the analysis of site-related follow-up duration and age effects. Kruskal-Wallis test (H) was used to analyze site differences in the time to transition variable, while χ^2 test was performed to test for an interaction between site and sex categories. P values were corrected for multiple comparisons using the False-Discovery rate (P_{FDR}) and statistical significance was determined at α=0.05. Significant comparisons in bold.

**Variables**	**Munich**	**Milan**	**Basel**	**Cologne**	**Birmingham**	**Turku**	**Udine**	***F/H/χ²***	***P* (P_{FDR})**
N	109	26	34	63	35	38	30		
Follow-up duration [mean (SD) days]	783.9 (323.7)	731.6 (281.9)	662.6 (295.8)	769.6 (301.0)	596.1 (199.1)	505.0 (126.7)	858.8 (414.2)	**6.54**	**<.001 (<.001)**
Transitions [No (%)]	10 (9.2)	2 (7.7)	2 (5.9)	4 (6.3)	2 (5.7)	6 (15.8)	0 (0.0)		
Time to transition [mean (SD) days]	227.0 (270.8)	526.0 (458.2)	26.0 (11.3)	311.3 (259.4)	115.5 (10.6)	261.7 (150.0)	--	5.81	.33 (.33)
Age [mean (SD) years]	24.5 (6.1)	24.8 (6.0)	24.3 (4.9)	24.0 (4.6)	23.6 (6.7)	26.1 (5.2)	27.2 (6.4)	1.76	.11 (.21)
Sex [Female (%)]	49.0 (45.0)	11.0 (44.0)	15 (44.1)	31.0 (49.2)	24.0 (66.7)	21 (56.8)	19 (63.3)	8.66	.19 (.26)

© 2020 Koutsouleris N et al. *JAMA Psychiatry.*

eTable 10. Effects of the Factor 'Site' on Predictive Performance of Raters, Unimodal, Stacked, and Cybernetic Risk Calculators

As Udine did not observe PT during the follow-up period, the χ^2 tests were carried out independently for true-positive and false-negative predictions (sensitivity, Udine excluded) and true-negative and false-positive predictions (specificity, Udine included). *P* values were corrected for multiple comparison using the False-Discovery Rate separately for the sensitivity and specificity-related analyses. Significant associations in bold.

	Munich	Milan	Basel	Cologne	Birmingham	Turku	Udine	All	$\chi^2(df)_{PT}$ / $\chi^2(df)_{NT}$	P_{PT} (FDR) / P_{NT} (FDR)
**Raters**										
TP (%)	7 (6.5)	2 (8.3)	1 (3.0)	3 (4.8)	0 (0.0)	3 (8.1)		16	5.51 (5)	.67
FN (%)	3 (2.8)	0 (0.0)	1 (3.0)	1 (1.6)	2 (5.7)	3 (8.1)		260		
TN (%)	85 (78.7)	15 (62.5)	29 (87.9)	50 (79.4)	31 (88.6)	27 (73.0)	23 (76.7)	44	10.95 (6)	.27
FP (%)	13 (12)	7 (29.2)	2 (6.1)	9 (14.3)	2 (5.7)	4 (10.8)	7 (23.3)	10		
All	108	24	33	63	35	37	30			
**Clinical-neurocognitive risk calculator**										
TP (%)	9 (8.3)	2 (8.0)	2 (5.9)	3 (4.8)	2 (5.6)	4 (10.8)		22	3.08 (5)	.69
FN (%)	1 (0.9)	0 (0.0)	0 (0.0)	1 (1.6)	0 (0.0)	2 (5.4)		205		
TN (%)	66 (60.6)	15 (60.0)	21 (61.8)	47 (74.6)	19 (52.8)	18 (48.6)	19 (63.3)	103	7.47 (6)	.56
FP (%)	33 (30.3)	8 (32.0)	11 (32.4)	12 (19.0)	15 (41.7)	13 (35.1)	11 (36.7)	4		
All	109	25	34	63	36	37	30			
**PRS-based risk calculator**										
TP (%)	8 (7.6)	2 (10.5)	1 (3.2)	3 (5.2)	2 (8.0)	3 (8.6)		19	3.89 (5)	.68
FN (%)	2 (1.9)	0 (0.0)	0 (0.0)	1 (1.7)	0 (0.0)	3 (8.6)		148		
TN (%)	54 (51.4)	9 (47.4)	18 (58.1)	33 (56.9)	9 (36)	13 (37.1)	12 (48.0)	125	5.24 (6)	.62
FP (%)	41 (39.0)	8 (42.1)	12 (38.7)	21 (36.2)	14 (56)	16 (45.7)	13 (52.0)	6		
All	105	19	31	58	25	35	25			
**sMRI-based risk calculator**										
TP (%)	8 (7.5)	1 (4.0)	2 (5.9)	3 (4.8)	2 (6.1)	6 (16.7)		22	4.75 (5)	.67
FN (%)	1 (0.9)	1 (4.0)	0 (0.0)	1 (1.6)	0 (0.0)	0 (0.0)		156		
TN (%)	52 (49.1)	10 (40.0)	19 (55.9)	32 (51.6)	13 (39.4)	16 (44.4)	14 (46.7)	145	3.32 (6)	.77
FP (%)	45 (42.5)	13 (52.0)	13 (38.2)	26 (41.9)	18 (54.5)	14 (38.9)	16 (53.3)	3		
All	106	25	34	62	33	36	30			
**Stacked risk calculator**										
TP (%)	9 (8.3)	2 (8.0)	2 (5.9)	2 (3.2)	2 (5.6)	4 (10.8)		21	5.18 (5)	.67
FN (%)	1 (0.9)	0 (0.0)	0 (0.0)	2 (3.2)	0 (0.0)	2 (5.4)		258		
TN (%)	81 (74.3)	16 (64.0)	24 (70.6)	57 (90.5)	25 (69.4)	29 (78.4)	26 (86.7)	50	**17.64 (6)**	**.04**
FP (%)	18 (16.5)	7 (28.0)	8 (23.5)	2 (3.2)	9 (25.0)	2 (5.4)	4 (13.3)	5		
All	109	25	34	63	36	37	30			
**Cybernetic risk calculator**										
TP (%)	10 (9.2)	2 (8.0)	2 (5.9)	3 (4.8)	1 (2.8)	4 (10.8)		22	6.16 (5)	.67
FN (%)	0 (0)	0 (0.0)	0 (0.0)	1 (1.6)	1 (2.8)	2 (5.4)		263		
TN (%)	86 (78.9)	17 (68.0)	29 (85.3)	53 (84.1)	27 (75.0)	25 (67.6)	26 (86.7)	45	5.81 (6)	.62
FP (%)	13 (11.9)	6 (24.0)	3 (8.8)	6 (9.5)	7 (19.4)	6 (16.2)	4 (13.3)	4		
All	109	25	34	63	36	37	30			

© 2020 Koutsouleris N et al. *JAMA Psychiatry.*

eTable 11. Heterogeneity of Raters' and Models' Predictive Performance

Cross-site means and standard deviations of sensitivity, specificity, balanced accuracy (BAC) and area-under-the-curve (AUC) were computed for the raters' prognostic estimates and the OOT predictions of unimodal, stacked, cybernetic and sequentially stacked risk calculators. For the computation of the mean (SD) of sensitivity, balanced accuracy, and AUC, we excluded Udine because the site lacked patients who developed psychosis during the follow-up period.

	**Site-level Sensitivity**	**Site-level Specificity**	**Site-level BAC**	**Site-level AUC**
**Sites available for mean (SD) computation**	6	7	6	6
**Rater-based prognostication**	57.5 (33.7)	82.8 (10.3)	69.9 (14.5)	0.71 (0.15)
**Unimodal risk calculators**				
Clinical-neurocognitive risk calculator	88.6 (14.6)	64.1 (8.8)	76.7 (5.5)	0.82 (0.06)
PRS-based risk calculator	84.2 (20.1)	54.8 (12.2)	70.7 (14.0)	0.62 (0.36)
sMRI-based risk calculator	85.6 (20.1)	53.4 (4.2)	69.8 (12.1)	0.73 (0.11)
**Stacked risk calculator**	84.4 (21.3)	83.6 (11.6)	83.9 (9.4)	0.90 (0.04)
**Cybernetic risk calculator**	81.9 (21.4)	84.8 (7.0)	83.0 (11.1)	0.90 (0.07)
**Sequential risk calculators (sequential stacking)**				
Clinical-neurocognitive model → Raters → PRS → sMRI	81.9 (21.4)	85.5 (7.6)	83.3 (11.1)	0.88 (0.08)

© 2020 Koutsouleris N et al. *JAMA Psychiatry.*

eTable 12. Differential Diagnostic Performance of Classification Models Trained to Separate Between CHR and ROD Patients Using Clinical-Neurocognitive, PRS-Based, and sMRI-Based Data Domain

Differential diagnostic performance of classification models trained to separate between CHR (=positive class) and ROD patients (=negative class) using clinical-neurocognitive, PRS-based, and sMRI-based data domains. In addition, raters' prognostic estimates were mapped to diagnostic labels to evaluate whether study group informed raters' predictions. Analyses were conducted in the complete PRONIA cohort.

CHR vs. ROD classification	TP	TN	FP	FN	Sens	Spec	BAC	PPV	NPV	PSI	LR+	AUC
Rater-based outcome estimates mapped to differential diagnostic task	51	156	9	114	30.9	94.5	62.7	84.0	57.8	42.8	5.7	0.63
Clinical-neurocognitive classifier [Clin-NC]	123	152	15	44	73.7	91.0	82.3	89.1	77.6	66.7	8.2	0.91
PRS-based classifier [PRS]	74	92	57	75	49.7	61.7	55.7	56.5	55.1	11.6	1.3	0.53
sMRI-based classifier [sMRI]	86	90	71	79	52.1	55.9	54.0	54.8	53.3	8.0	1.2	0.55
Stacked classifier [Stk]	126	147	20	41	75.4	88.0	81.7	86.3	78.2	64.5	6.3	0.91
Cybernetic classifier [Cyb]	128	150	17	39	76.6	89.8	83.2	88.3	79.4	67.6	7.5	0.91

Abbreviations: *TP* number of true positives, *TN* number of true negatives, *FP* number of false positives, *FN* number of false negatives, *Sens* Sensitivity, *Spec* Specificity, *BAC* Balanced Accuracy, *PPV* Positive Predictive Value, *NPV* Negative Predictive Value, *PSI* Prognostic Summary Index, *LR+* Positive Likelihood Ratio, *AUC* Area-under-the Curve.

© 2020 Koutsouleris N et al. *JAMA Psychiatry.*

eTable 13. Explained Variances of Pairwise Prognostic, Diagnostic, and Prognostic-Diagnostic Classifier Combinations

Decision scores of risk calculators trained to predict transition to psychosis (P:) or to differentially diagnose CHR and ROD study groups (DD:) were analyzed for pairwise associations using the squared Pearson correlation coefficient. Log-transformed, FDR-corrected *P* values in the lower table indicate whether pairwise associations were significant at *P*=0.05. Significant associations were marked in bold. Explained variances and *P* values were marked with different colors to distinguish between intra-prognostic (red), intra-diagnostic (blue) and prognostic-diagnostic (green) classifier associations.

**R^2**	P:Clin-NC	P:PRS	P:sMRI	P:Stk	P:Cyb	DD:Clin-NC	DD:PRS	DD:sMRI	DD:Stk	DD:Cyb
P:Clin-NC		0.010	0.007	**0.674**	**0.519**	**0.497**	0.001	0.000	**0.478**	**0.489**
P:PRS			0.000	**0.253**	**0.241**	0.008	**0.183**	0.000	0.007	0.007
P:sMRI				**0.110**	**0.128**	0.001	0.001	0.002	0.003	0.003
P:Stk					**0.748**	**0.357**	**0.047**	0.006	**0.359**	**0.356**
P:Cyb						**0.310**	**0.056**	0.004	**0.295**	**0.350**
DD:Clin-NC							0.006	0.001	**0.968**	**0.960**
DD:PRS								0.001	0.006	0.006
DD:sMRI									**0.018**	0.015
DD:Stk										**0.976**
DD:Cyb										
**-log(P_{FDR})**	P:Clin-NC	P:PRS	P:sMRI	P:Stk	P:Cyb	DD:Clin-NC	DD:PRS	DD:sMRI	DD:Stk	DD:Cyb
P:Clin-NC		0.78	0.61	**70.36**	**46.13**	**43.41**	0.15	0.09	**41.07**	**42.40**
P:PRS			0.09	**18.80**	**17.83**	0.63	**13.23**	0.02	0.61	0.59
P:sMRI				**7.80**	**9.06**	0.15	0.14	0.26	0.36	0.33
P:Stk					**86.40**	**28.14**	**3.38**	0.56	**28.31**	**28.01**
P:Cyb						**23.76**	**3.98**	0.41	**22.44**	**27.48**
DD:Clin-NC							0.56	0.15	**215.42**	**200.96**
DD:PRS								0.15	0.56	0.56
DD:sMRI									**1.39**	1.18
DD:Stk										**234.18**
DD:Cyb										

Abbreviations: *P:/DD:Clin-NC* Clinical-neurocognitive risk calculator/different diagnostic classifier, *P:/DD:PRS* PRS-based risk calculator/different diagnostic classifier, *P:/DD:sMRI* sMRI-based risk calculator/different diagnostic classifier, *P:/DD:Stk* Stacked risk calculator/different diagnostic classifier, *P:/DD:Cyb* Cybernetic risk calculator/different diagnostic classifier.

© 2020 Koutsouleris N et al. *JAMA Psychiatry.*

eTable 14. ROD Depletion and Substitution Analyses Assessing the Performance Effects Induced by the ROD Group in the Prediction of Psychosis Transitions in the CHR Sample

ROD depletion and replacement analyses were carried out to assess the performance effects induced by training unimodal and multi-modal risk calculators with ROD patients. Both sets of analyses were carried out in the complete PRONIA cohort using the identical parameter settings of our main analyses. First, out-of-training performance was measured only in the CHR sample using Leave-One-Site-Out Cross-Validation (LOSOCV, see **section 1.5.1**). Second, in the depletion analysis, we removed ROD patients from the training population and re-run the analysis just in the CHR patients. Third, in the substitution analysis, we replaced ROD patients with 167 age, sex and site-matched HCs and measured the prediction performance of these prognostic classifiers again only in the CHR patients. For the sMRI-based risk calculator, we were able to use the Fe*Psy* and ZInEP cohorts to externally validate the losses of prognostic performance observed in the PRONIA CHR cohort. See **section 1.5.7** for further methodological details and **eFigure 12** for a statistical analysis of performance differences (BAC) between the original models and their ROD-depleted or HC-substituted counterparts.

**Prediction performance in CHR and ROD patients**	**TP**	**TN**	**FP**	**FN**	**Sens**	**Spec**	**BAC**	**PPV**	**NPV**	**PSI**	**LR+**	**AUC**
**Clinical-neurocognitive risk calculator**												
Performance of the original model	20	57	87	3	87.0	39.6	63.3	18.7	95.0	13.7	1.44	0.71
Model performance after removing ROD patients	14	84	60	9	60.9	58.3	59.6	18.9	90.3	9.2	1.50	0.62
Model performance after replacing ROD patients with HC individuals	22	26	118	1	95.7	18.1	56.9	15.7	96.3	12.0	1.15	0.66
**PRS-based risk calculator**												
Performance of the original model	20	72	55	2	90.9	56.7	73.8	26.7	97.3	24.0	2.10	0.73
Model performance after removing ROD patients	15	68	59	7	68.2	53.5	60.9	20.3	90.7	10.9	1.50	0.65
Model performance after replacing ROD patients with HC individuals	17	63	64	5	77.3	50.4	63.4	21.0	92.7	13.6	1.53	0.63
**sMRI-based risk calculator**												
Performance of the original model												
LOSOCV (PRONIA CHR sample)	19	79	64	3	86.4	55.3	70.8	22.9	96.4	19.2	1.93	0.73
FePsy sample	*12*	*13*	*8*	*4*	*75.0*	*61.9*	*68.5*	*60.0*	*76.5*	*36.5*	*2.0*	*0.71*
ZInEP sample	*12*	*79*	*51*	*4*	*75.0*	*60.8*	*67.9*	*19.0*	*95.2*	*14.2*	*1.9*	*0.71*
Model performance after removing ROD patients												
LOSOCV (PRONIA CHR sample)	18	83	60	4	81.8	58.0	69.9	23.1	95.4	18.5	2.00	0.72
FePsy sample	*8*	*13*	*8*	*8*	*50.0*	*61.9*	*56.0*	*50.0*	*61.9*	*11.9*	*1.30*	*0.64*
ZInEP sample	*12*	*71*	*59*	*4*	*75.0*	*54.6*	*64.8*	*16.9*	*94.7*	*11.6*	*1.70*	*0.68*
Model performance after replacing ROD patients with HC individuals												
LOSOCV (PRONIA CHR sample)	16	84	59	6	72.7	58.7	65.7	21.3	93.3	14.7	1.76	0.65
FePsy sample	*11*	*12*	*9*	*5*	*68.8*	*57.1*	*62.9*	*55.0*	*70.6*	*25.6*	*1.60*	*0.64*
ZInEP sample	*11*	*75*	*55*	*5*	*68.8*	*57.7*	*63.2*	*16.7*	*93.8*	*10.4*	*1.63*	*0.67*
**Stacked risk calculator**												
Performance of the original model	17	105	39	6	73.9	72.9	73.4	30.4	94.6	25.0	2.73	0.82
Model performance after removing ROD patients	10	111	33	13	43.5	77.1	60.3	23.3	89.5	12.8	1.90	0.73
Model performance after replacing ROD patients with HC individuals	17	90	54	6	73.9	62.5	68.2	23.9	93.8	17.7	1.97	0.77

Abbreviations: *LOSOCV* leave-site-out cross-validation performance of model in predicting psychosis transition in the CHR patients, *TP* number of true positives, *TN* number of true negatives, *FP* number of false positives, *FN* number of false negatives, *Sens* Sensitivity, *Spec* Specificity, *BAC* Balanced Accuracy, *PPV* Positive Predictive Value, *NPV* Negative Predictive Value, *PSI* Prognostic Summary Index, *LR+* Positive Likelihood Ratio, *AUC* Area-under-the Curve.

© 2020 Koutsouleris N et al. *JAMA Psychiatry.*

eTable 15. Discriminative Performance of Raters and Risk Calculators in Distinguishing Between Transition Cases, Cases With Nonremitting/De novo CHR States and Cases Developing Asymptomatic CHR Trajectories

Analysis of the raters' and risk calculators' discriminative performance in separating patients with persistent/de-novo psychosis risk (P-CHR) states from patients with disease transitions (PT) and patients with asymptomatic risk states (NP-CHR). The upper part of the table shows the observed balanced accuracies (BAC) measured across the different classifiers for each of the three binary discrimination tasks. P values were obtained by performing 5000 random permutations of the classifiers' decision scores and comparing the BAC of the permuted classifiers with the observed BAC. The lower part of the table lists the pairwise BAC differences (ΔBAC) of the different classifier combinations. Significance was established by computing the permuted ΔBAC and comparing them to the respective observed ΔBAC. An alpha correction was carried for the upper and lower parts of the table using the FDR. Significance was established at *P*=0.05 and respective values were marked in bold.

	**PT vs. P-CHR**	**PT vs. NP-CHR**	**PR vs. NP-CHR**
**Classifiers (Out-Of-Training [OOT] performance [P_{FDR} value])**			
Raters	**68.55 [<.001]**	**76.47 [<.001]**	**57.93 [.002]**
Clinical-neurocognitive [Clin-NC]	**66.48 [.002]**	**82.23 [<.001]**	**65.75 [<.001]**
PRS-based	**62.68 [.02]**	**68.24 [<.001]**	55.57 [.06]
sMRI-based	**71.22 [<.001]**	**70.38 [<.001]**	49.16 [.58]
Stacked	**78.85 [<.001]**	**85.73 [<.001]**	**56.89 [.009]**
Cybernetic	**79.12 [<.001]**	**89.98 [<.001]**	**60.86 [<.001]**
Sequence	**80.77 [<.001]**	**89.59 [<.001]**	**58.83 [.001]**
**Pairwise classifier comparisons (OOT performance difference [P_{FDR} value])**			
Raters vs. Clin-NC	2.06 [.79]	-5.76 [.43]	-7.82 [.06]
Raters vs. PRS-based	5.87 [.51]	8.23 [.35]	2.36 [.69]
Raters vs. sMRI-based	-2.68 [.78]	6.09 [.50]	8.77 [.10]
Raters vs. Stacked	-10.30 [.13]	-9.26 [.16]	1.04 [.80]
Raters vs. Cybernetic	**-10.57 [.02]**	**-13.51 [.008]**	-2.94 [.31]
Raters vs. Sequence	**-12.22 [.02]**	**-13.12 [.008]**	-0.90 [.78]
Clin-NC vs. PRS-based	3.81 [.70]	13.99 [.11]	10.18 [.06]
Clin-NC vs. sMRI-based	-4.74 [.64]	11.85 [.22]	**16.59 [.008]**
Clin-NC vs. Stacked	**-12.36 [.03]**	-3.50 [.55]	**8.86 [.01]**
Clin-NC vs. Cybernetic	**-12.64 [.04]**	-7.75 [.22]	4.89 [.22]
Clin-NC vs. Sequence	**-14.29 [.02]**	-7.36 [.22]	6.92 [.06]
PRS-based vs. sMRI-based	-8.55 [.39]	-2.14 [.80]	6.41 [.30]
PRS-based vs. Stacked	**-16.17 [.04]**	**-17.49 [.03]**	-1.32 [.79]
PRS-based vs. Cybernetic	**-16.45 [.03]**	**-21.74 [.008]**	-5.29 [.32]
PRS-based vs. Sequence	**-18.09 [.03]**	**-21.35 [.008]**	-3.26 [.55]
sMRI-based vs. Stacked	-7.62 [.35]	**-15.36 [.04]**	-7.73 [.11]
sMRI-based vs. Cybernetic	-7.90 [.33]	**-19.60 [.008]**	**-11.71 [.02]**
sMRI-based vs. Sequence	-9.55 [.23]	**-19.22 [.01]**	**-9.67 [.04]**
Stacked vs. Cybernetic	-0.27 [.97]	-4.25 [.39]	-3.97 [.22]
Stacked vs. Sequence	-1.92 [.75]	-3.86 [.43]	-1.94 [.55]
Cybernetic vs. Sequence	-1.65 [.37]	0.39 [.87]	**2.04 [.04]**

© 2020 Koutsouleris N et al. *JAMA Psychiatry.*

eTable 16. Comparison of Nonpsychotic Diagnostic Outcomes Between Patients With a Predicted Transition vs Predicted Nontransition to Psychosis During the Follow-up Period of the Study

Patients were included in the analysis if they had a baseline and at least one SCID-IV follow-up examination. Comparisons were carried out to explore whether raters or risk calculators were not specifically predicting transition to psychotic disorders but also other relevant diagnostic outcomes. In addition, the comparisons were carried out for the observed outcome groups. The definition of diagnostic outcomes is detailed in section **1.5.8**. All *P* values were corrected for multiple comparisons using the False-Discovery-Rate (P_{FDR}).

	**Raters**				**Clinical-neurocognitive risk calculator**				**PRS-based risk calculator**				**sMRI-based risk calculator**				**Cybernetic risk calculator**				**Observed outcomes**			
**Diagnostic variables**	**PT_p (%)**	**NT_p (%)**	**χ^2**	**P_{FDR}**	**PT_p (%)**	**NT_p (%)**	**χ^2**	**P_{FDR}**	**PT_p (%)**	**NT_p (%)**	**χ^2**	**P_{FDR}**	**PT_p (%)**	**NT_p (%)**	**χ^2**	**P_{FDR}**	**PT_p (%)**	**NT_p (%)**	**χ^2**	**P_{FDR}**	**PT_p (%)**	**NT_p (%)**	**χ^2**	**P_{FDR}**
**Major depressive disorder (MDD)**																								
None	18 (31.6)	65 (24.3)	13.0	.09	41 (33.6)	44 (21.3)	11.5	.09	34 (23.9)	36 (24.2)	0.3	1.00	42 (25.1)	43 (27.2)	0.6	1.00	20 (31.3)	65 (24.5)	11.4	.09	5 (22.7)	80 (26.1)	3.6	1.00
Remission	11 (19.3)	70 (26.1)			27 (22.1)	55 (26.6)			34 (23.9)	40 (26.1)			40 (24.0)	41 (25.9)			11 (17.2)	71 (26.8)			5 (22.7)	77 (25.1)		
Non-Remission	19 (33.3)	123 (45.9)			43 (35.2)	100 (48.3)			65 (45.8)	67 (43.8)			75 (44.9)	65 (41.1)			24 (37.5)	119 (44.9)			11 (50.0)	132 (43.0)		
Occurrence	9 (15.8)	10 (3.7)			11 (9.0)	8 (3.9)			9 (6.3)	9 (5.9)			10 (6.0)	9 (5.7)			9 (14.1)	10 (3.8)			1 (4.5)	18 (5.9)		
**Affective disorders (excl. MDD)**																								
None	53 (93.0)	237 (88.4)	0.8	1.00	104 (85.2)	190 (91.8)	3.8	.77	129 (90.8)	133 (86.9)	1.5	.94	153 (91.6)	137 (86.7)	3.3	.78	59 (92.2)	235 (88.7)	1.3	.96	20 (90.9)	274 (89.3)	2.2	.87
Remission	2 (3.5)	11 (4.1)			7 (5.7)	6 (2.9)			6 (4.2)	7 (4.6)			5 (3.0)	8 (5.1)			1 (1.6)	12 (4.5)			0 (0.0)	13 (4.2)		
Non-Remission	1 (1.8)	8 (1.8)			5 (4.1)	4 (1.9)			3 (2.1)	6 (3.9)			5 (3.0)	4 (2.5)			1 (1.6)	8 (3.0)			0 (0.0)	9 (2.9)		
Occurrence	1 (1.8)	12 (4.5)			6 (4.9)	7 (3.4)			4 (2.8)	7 (4.6)			4 (2.4)	9 (5.7)			3 (4.7)	10 (3.8)			2 (9.1)	11 (3.6)		
**Anxiety disorders**																								
None	32 (56.1)	182 (67.9)	5.5	.48	74 (60.7)	143 (69.1)	6.1	.48	92 (64.8)	102 (66.7)	1.7	.94	116 (69.5)	98 (62.0)	2.2	.90	38 (59.4)	179 (67.5)	2.7	.87	14 (63.6)	203 (66.1)	1.1	.97
Remission	2 (3.5)	14 (5.2)			10 (8.2)	6 (2.9)			9 (6.3)	5 (3.3)			8 (4.8)	8 (5.1)			4 (6.3)	12 (4.5)			1 (4.5)	15 (4.9)		
Non-Remission	15 (26.3)	37 (13.8)			23 (18.9)	30 (14.5)			23 (16.2)	28 (18.3)			24 (14.4)	29 (18.4)			14 (21.9)	39 (14.7)			5 (22.7)	48 (15.6)		
Occurrence	8 (14.0)	35 (13.1)			15 (12.3)	28 (13.5)			18 (12.7)	18 (11.8)			19 (11.4)	23 (14.6)			8 (12.5)	35 (13.2)			2 (9.1)	41 (13.4)		
**Substance dependency disorders**																								
None	52 (91.2)	252 (94.0)	2.7	.87	114 (93.4)	194 (93.7)	0.6	1.00	129 (90.8)	145 (94.8)	2.1	.94	156 (93.4)	148 (93.7)	1.8	.94	55 (85.9)	253 (95.5)	8.3	.19	20 (90.9)	288 (93.8)	5.5	.48

© 2020 Koutsouleris N et al. *JAMA Psychiatry.*

Remission	1 (1.8)	1 (0.4)			1 (0.8)	1 (0.5)			1 (0.7)	1 (0.7)			2 (1.2)	0 (0.0)			0 (0.0)	1 (0.4)			1 (4.5)	1 (0.3)		
Non-Remission	2 (3.5)	6 (2.2)			3 (2.5)	5 (2.4)			5 (3.5)	3 (2.0)			4 (2.4)	4 (2.5)			3 (4.7)	5 (1.9)			1 (4.5)	7 (2.3)		
Occurrence	2 (3.5)	9 (3.4)			4 (3.3)	7 (3.4)			7 (4.9)	4 (2.6)			5 (3.0)	6 (3.8)			5 (7.8)	6 (2.3)			0 (0.0)	11 (3.6)		
**Eating disorders**																								
None	51 (89.5)	250 (93.3)	5.6	.48	110 (90.2)	195 (94.2)	2.5	.87	130 (91.5)	145 (94.8)	4.1	.87	157 (94.0)	144 (91.1)	2.1	.90	56 (87.5)	249 (94.0)	4.6	.55	20 (90.9)	285 (92.8)	2.0	.78
Remission	1 (1.8)	4 (1.5)			2 (1.6)	3 (1.4)			4 (2.8)	1 (0.7)			2 (1.2)	3 (1.9)			1 (1.6)	4 (1.5)			1 (4.5)	4 (1.3)		
Non-Remission	0 (0.0)	7 (2.6)			4 (3.3)	3 (1.4)			4 (2.8)	1 (0.7)			4 (2.4)	3 (1.9)			3 (4.7)	4 (1.5)			0 (0.0)	7 (2.3)		
Occurrence	5 (8.8)	7 (2.6)			6 (4.9)	6 (2.9)			4 (2.8)	6 (3.9)			4 (2.4)	8 (1.9)			4 (6.3)	8 (3.0)			1 (4.5)	11 (3.6)		

© 2020 Koutsouleris N et al. *JAMA Psychiatry.*

eTable 17. Prognostic Sequences Tested in the Sequence Optimization Algorithm

The winning sequence was marked in red. See also **eFigure 14** for an in-depth analysis of the winning sequence.

No.	Uni-modal	Two components	Three components	Four components
1	P			
2	P	P → C		
3	P	P → G		
4	P	P → M		
5	P	P → C	P → C → G	
6	P	P → C	P → C → M	
7	P	P → C	P → C → G	P → C → G → M
8	P	P → C	P → C → M	P → C → M → G
9	P	P → G	P → G → C	
10	P	P → G	P → G → M	
11	P	P → G	P → G → C	P → G → C → M
12	P	P → G	P → G → M	P → G → M → C
13	P	P → M	P → M → C	
14	P	P → M	P → M → G	
15	P	P → M	P → M → C	P → M → C → G
16	P	P → M	P → M → G	P → M → G → C
17	C			
18	C	C → P		
19	C	C → G		
20	C	C → M		
21	C	C → P	C → P → G	
22	C	C → P	C → P → M	
23	C	C → P	C → P → G	C → P → G → M
24	C	C → P	C → P → M	C → P → M → G
25	C	C → G	C → G → P	
26	C	C → G	C → G → M	
27	C	C → G	C → G → P	C → G → P → M
28	C	C → G	C → G → M	C → G → M → P
29	C	C → M	C → M → P	
30	C	C → M	C → M → G	
31	C	C → M	C → M → P	C → M → P → G
32	C	C → M	C → M → G	C → M → G → P
33	G			
34	G	G → P		
35	G	G → C		
36	G	G → M		
37	G	G → P	G → P → C	
38	G	G → P	G → P → M	
39	G	G → P	G → P → C	G → P → C → M
40	G	G → P	G → P → M	G → P → M → G
41	G	G → C	G → C → P	
42	G	G → C	G → C → M	
43	G	G → C	G → C → P	G → C → P → M
44	G	G → C	G → C → M	G → C → M → P
45	G	G → M	G → M → P	
46	G	G → M	G → M → C	
47	G	G → M	G → M → P	G → M → P → C
48	G	G → M	G → M → C	G → M → C → P
49	M			
50	M	M → P		
51	M	M → C		
52	M	M → G		
53	M	M → P	M → P → C	
54	M	M → P	M → P → G	
55	M	M → P	M → P → C	M → P → C → G
56	M	M → P	M → P → G	M → P → G → C
57	M	M → C	M → C → P	
58	M	M → C	M → C → G	
59	M	M → C	M → C → P	M → C → P → G
60	M	M → C	M → C → G	M → C → G → P
61	M	M → G	M → G → P	
62	M	M → G	M → G → C	
63	M	M → G	M → G → P	M → G → P → C
64	M	M → G	M → G → C	M → G → C → P

Abbreviations: *C* Clinical model, *G* PRS-based genetic model, *M* sMRI-based model, *P* Raters' prognostic estimates

© 2020 Koutsouleris N et al. *JAMA Psychiatry.*

eTable 18. Study-Related, Sociodemographic, Physical, Functional, and Clinical Differences in the ZInEP Cohort

Parametric and non-parametric tests were used to compare transition to non-transition cases in baseline variables matching closely those analyzed in **Table 1** of the main manuscript. *P* values were corrected for multiple comparisons using the False-Discovery Rate (P_{FDR}). Significant comparisons were marked in bold.

Variables	PT	NT	*df*	*T/W/χ²*	P_{FDR}
**Samples and study variables**					
Sample sizes	16	130			
**Sociodemographic data**					
Age [mean (SD) years]	20.4 (3.8)	22.8 (5.8)	–	1256	.37
Sex [F (%)]	5 (31)	53 (41)	1	0.24	.98
Ethnicity [Caucasian]	16	130			
Handedness [No. right/left/ambi (%left)]	15/1/0 (6)	115/12/3 (9)	2	0.56	1.00
Education [mean (SD) level]	3.47 (1.6)	3.43 (1.7)	–	951	1.00
Educational problems [frequency (%) years re-	6 (67)	37 (29)	1	0.32	.93
Having a partnership most of the time in the year	4 (27)	36 (28)	1	2.54e-30	1.00
**CHR criteria [Yes (%)]**					
Schizotypal personality disorder present	3 (18.8)	8 (6.3)	1	1.57	.42
1st degree relatives with psychosis	1 (6.2)	6 (4.8)	1	1.13e-30	1
30%-loss of global functioning compared to highest	7 (43.8)	32 (25.4)	1	1.57	.42
GRFD criteria met	4 (20.0)	4 (3.1)	1	**9.33**	**.01**
COGDIS criteria met	11 (68.8)	72 (55.4)	1	0.56	.79
APS criteria met	9 (69.2)	42 (28.6)	1	2.62	.31
BLIPS criteria met	1 (6.2)	4 (3.1)	1	3.85e-31	1.00
**Functioning [mean (SD)]**					
GAF Highest Lifetime	65.94	72.56 (13.42)	–	1225	.33
GAF Baseline	45.94	58.12 (14.31)	–	**1552.5**	**.009**
**High-risk symptoms [mean (SD)]**					
SIPS Positive Symptoms	1.98 (0.97)	1.28 (0.87)	–	**590**	**.02**
SIPS Negative Symptoms	2.71 (0.80)	1.85 (1.02)	–	**526**	**.009**
SIPS Disorganized Symptoms	1.44 (0.59)	0.89 (0.69)	–	**500.5**	**.009**
SIPS General Psychopathology	2.25 (0.87)	1.89 (0.94)	–	753	.23
**History of ICD-10 comorbid disorders at study inclusion [No. (%)]**					
Any comorbid affective, substance, anxiety, eating disorders before study inclusion					
No diagnosis	1 (6.3)	16 (12.3)	3	0.65	1.00
1 diagnosis	5 (31.2)	42 (32.3)			
2 diagnoses	5 (31.2)	33 (25.4)			
3 or more diagnoses	5 (31.2)	39 (30)			
Comorbid affective disorders					
No diagnosis	3 (18.8)	39 (30)	2	1.80	.75
1 diagnosis	9 (56.2)	73 (56.2)			
2 diagnoses	4 (25)	18 (13.8)			
Comorbid substance disorders					
No diagnosis	10 (62.5)	119 (91.5)	2	**9.02**	**.02**
1 diagnosis	6 (37.5)	11 (8.5)			
3 or more diagnoses	0 (0)	0 (0)			
Comorbid anxiety disorders					
No diagnosis	8 (50)	56 (41.3)	3	1.52	.98
1 diagnosis	4(25)	42 (32.3)			
2 diagnoses	2 (12.5)	24 (18.5)			
3 or more diagnoses	2(12.5)	8 (6.2)			
Comorbid eating disorders					
No diagnosis	16 (100)	127 (97.7)	1	8.97e-31	1.00
1 diagnosis	0 (0)	3 (2.3)			
2 diagnoses	0 (0)	0 (0)			
**Treatments [No (%)]**					
received antipsychotics	7 (43.8)	20 (15.4)	1	5.84	.06
received antidepressants	4 (28.6)	31 (25.2)	1	3.55e-31	1.00

Abbreviations: *BLIPS* brief limited intermittent psychotic symptoms, *COGDIS* Cognitive Disturbances (SPI-A) criteria, *df* degrees of freedom, *F* analysis of variance statistic, *GAF* Global Assessment of Functioning scores, *GRFD* Genetic Risk and Functional Decline risk syndrome, *PT* cases with transition to psychosis, *NT* non-transition cases, *SIPS* Structured Interview for Psychosis risk Syndromes, *SD* standard deviation, *T* Student *t* statistic, *W* Wilcoxon score of the Mann-Whitney U Test.

© 2020 Koutsouleris N et al. *JAMA Psychiatry.*

eTable 19. Study-Related, Sociodemographic, Physical, Functional, and Clinical Differences in the BEARS-Kid Cohort

Parametric and non-parametric tests were used to compare patients with (PT) vs. without transition to psychosis (NT) during the follow-up period in variables matching closely those analyzed in Table 1 of the main manuscript. *P* values were corrected for multiple comparisons in the respective table columns using the False-Discovery Rate (P_{FDR}). Significant differences were marked in bold.

Variables	PT	NT	*df*	$T/Z/\chi^2$	P_{FDR}
**Samples and study variables**					
Sample sizes	13	449			
**Sociodemographic data**					
Age [mean (SD) years]	15.9 (1.5)	14.6 (2.4)	13.8	**2.86**	**.05**
Sex [F (%)]	6 (46.2)	260 (58.0)	1	0.73	.49
Nationality [%]					
Swiss	8 (61.5)	249 (55.5)	4	0.26	.99
German	4 (30.8)	161 (35.9)			
French	0 (0.0)	1 (0.2)			
Italian	0 (0.0)	2 (0.4)			
Other	1 (7.7)	36 (8.0)			
Current class level [mean (SD) level]	9.0 (1.9)	7.8 (2.4)	400	1.64	.20
School graduation [concluded (%)]	3 (23.1)	53 (11.8)	1	1.51	.49
Partnership at study inclusion [Yes (%)]	1 (7.7)	59 (13.4)	1	0.36	.79
**CHR criteria [Yes (%)]**					
Schizotypal personality disorder acc. to SIPS	0	7 (1.6)	1	0.24	1.00
1st degree relatives with psychosis	1 (7.7)	17 (3.8)	1	0.56	1.00
30%-loss of global functioning compared to highest levels in the year before study inclusion	6 (46.2)	74 (16.5)	1	**7.77**	**.05**
GRFD criteria met	1 (7.7)	11 (2.5)	1	1.37	.43
COPER criteria met	5 (38.5)	105 (23.4)	1	1.60	.47
COGDIS criteria met	8 (61.5)	71 (15.8)	1	**18.6**	**.005**
APS criteria met	8 (61.5)	105 (23.4)	1	**9.91**	**.02**
BLIPS criteria met	1 (7.7)	6 (1.3)	1	3.41	.30
**Functioning [mean (SD)]**					
GF:S Highest Last Year	7.54 (1.27)	7.12 (1.15)	–	1.14	.39
GF:S Baseline	6.46 (1.85)	6.60 (1.16)	–	-0.49	.72
**High-risk symptoms [mean (SD)]**					
SIPS Positive Symptoms	1.78 (0.98)	0.89 (0.86)	460	**3.67**	**.005**
SIPS Negative Symptoms	1.95 (1.19)	1.16 (0.88)	12.4	2.38	.09
SIPS Disorganized Symptoms	1.17 (0.83)	0.69 (0.54)	12.3	2.12	.12
SIPS General Psychopathology	2.32 (1.28)	1.40 (0.95)	460	**3.42**	**.006**
Mean of 9 COGDIS symptoms	1.12 (0.69)	0.40 (0.78)	460	**3.30**	**.006**
Mean of 10 COPER symptoms	1.14 (0.74)	0.46 (0.76)	460	**3.20**	**.006**
**DSM-IV mental disorders according to M.I.N.I.-Kid [No. (%)]**					
Any past mental disorder incl. specific phobia prior to study inclusion					
No diagnosis	6 (46.2)	309 (69.0)	3	8.67	0.09
1 diagnosis	3 (23.1)	97 (21.0)			
2 diagnoses	4 (30.8)	36 (8.0)			
3 or more diagnoses	0 (0.0)	6 (1.3)			
Any mental disorder incl. specific phobia at study inclusion					
No diagnosis	2 (15.4)	35 (7.8)	3	7.55	.12
1 diagnosis	5 (38.5)	211 (47.1)			
2 diagnoses	0 (0.0)	105 (23.4)			
3 or more diagnoses	6 (46.2)	97 (21.7)			
Any mental disorder excl. specific phobia at study inclusion					
No diagnosis	4 (30.8)	51 (11.4)	3	**12.7**	**.02**
1 diagnosis	3 (23.1)	201 (44.9)			
2 diagnoses	0 (0.0)	107 (23.9)			
3 or more diagnoses	6 (46.2)	89 (19.9)			
Affective disorders at study inclusion					
No diagnosis	7 (53.8)	291 (65.0)	3	5.37	.26
1 diagnosis	1 (7.7)	86 (19.2)			
2 diagnoses	5 (38.5)	69 (15.4)			
3 or more diagnoses	0 (0.0)	2 (0.4)			

© 2020 Koutsouleris N et al. *JAMA Psychiatry.*

Substance-related disorders at study inclusion					
No diagnosis	11 (84.6)	431 (96.2)	2	5.74	.12
1 diagnosis	2 (15.4)	14 (3.1)			
2 diagnoses	0 (0.0)	3 (0.7)			
Anxiety disorders at study inclusion					
No diagnosis	8 (61.5)	304 (67.9)	3	3.03	.49
1 diagnosis	4 (30.8)	116 (25.9)			
2 diagnoses	0 (0.0)	20 (4.5)			
3 or more diagnoses	1 (7.7)	8 (1.8)			
Eating disorders at study inclusion					
No diagnosis	13 (100)	346 (77.2)	2	3.80	.26
1 diagnosis	0 (0.0)	100 (22.3)			
2 diagnoses	0 (0.0)	2 (0.4)			

Abbreviations: *BLIPS* brief limited intermittent psychotic symptoms, χ^2 Chi-square of the Chi-square test, *COGDIS* Schizophrenia Proneness Instrument: Cognitive Disturbances criteria, *COPER* Schizophrenia Proneness Instrument: Cognitive-Perceptive Basic Symptoms criteria, *df* degrees of freedom, *GF:S/GF:R* Global functioning scales: Social and Role, *GRFD* Genetic Risk and Functional Decline risk syndrome, *M.I.N.I.-Kid* Mini-International Neuropsychiatric Interview - children's version , *SIPS* Structured Interview for Psychosis risk Syndromes, *SD* standard deviation, *T* Student *t* statistic, *Z* z-score of the Mann-Whitney U Test.

© 2020 Koutsouleris N et al. *JAMA Psychiatry.*

eTable 20. Performance Gains Produced by the Stacking of the Clinical-Neurocognitive and PRS-Based Models

Abbreviations are provided in **Table 2**, main manuscript.

	TP	TN	FP	FN	Sens	Spec	BAC	PPV	NPV	PSI	LR+	AUC
**Clinical-neurocognitive risk calculator**												
PRONIA+18M cohort	22	147	73	4	84.6	66.8	75.7	23.2	97.4	20.5	2.6	0.83
PRONIA-18M cohort	–	*58*	*30*	–	–	*65.9*	–	–	–	–	–	–
**PRS-based risk calculator**												
PRONIA+18M cohort	19	113	88	6	76.0	56.2	66.1	17.8	95.0	12.7	1.7	0.74
PRONIA-18M cohort	–	*35*	*37*	–	–	*48.6*	–	–	–	–	–	–
**Stacked risk calculator analyzing predictions of the clinical-neurocognitive and PRS-based models**												
PRONIA+18M cohort	21	178	42	5	80.8	80.9	80.8	33.3	97.3	30.6	4.2	0.85
PRONIA-18M cohort	–	*69*	*19*	–	–	*78.4*	–	–	–	–	–	–

© 2020 Koutsouleris N et al. *JAMA Psychiatry.*

eFigure 1. CONSORT Chart and Follow-up Protocol of the PRONIA Study for the Clinical Participants

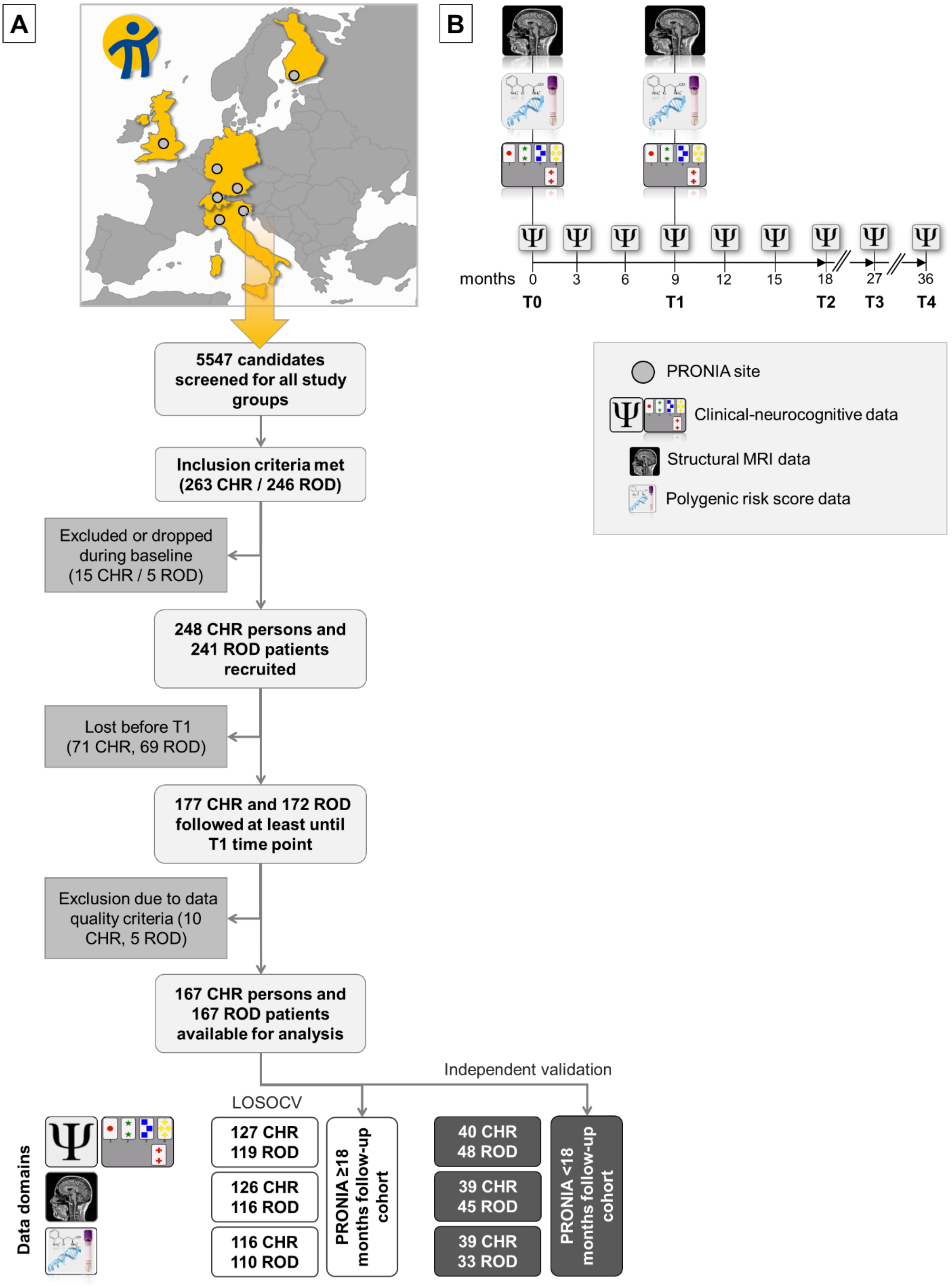

Abbreviations: *CHR* Clinical-High Risk patients, *LOSOCV* Leave-One-Site-Out Cross-Validation, *ROD* Recent-Onset Depression

© 2020 Koutsouleris N et al. *JAMA Psychiatry.*

eFigure 2. Schematic Analysis Workflow of the Study

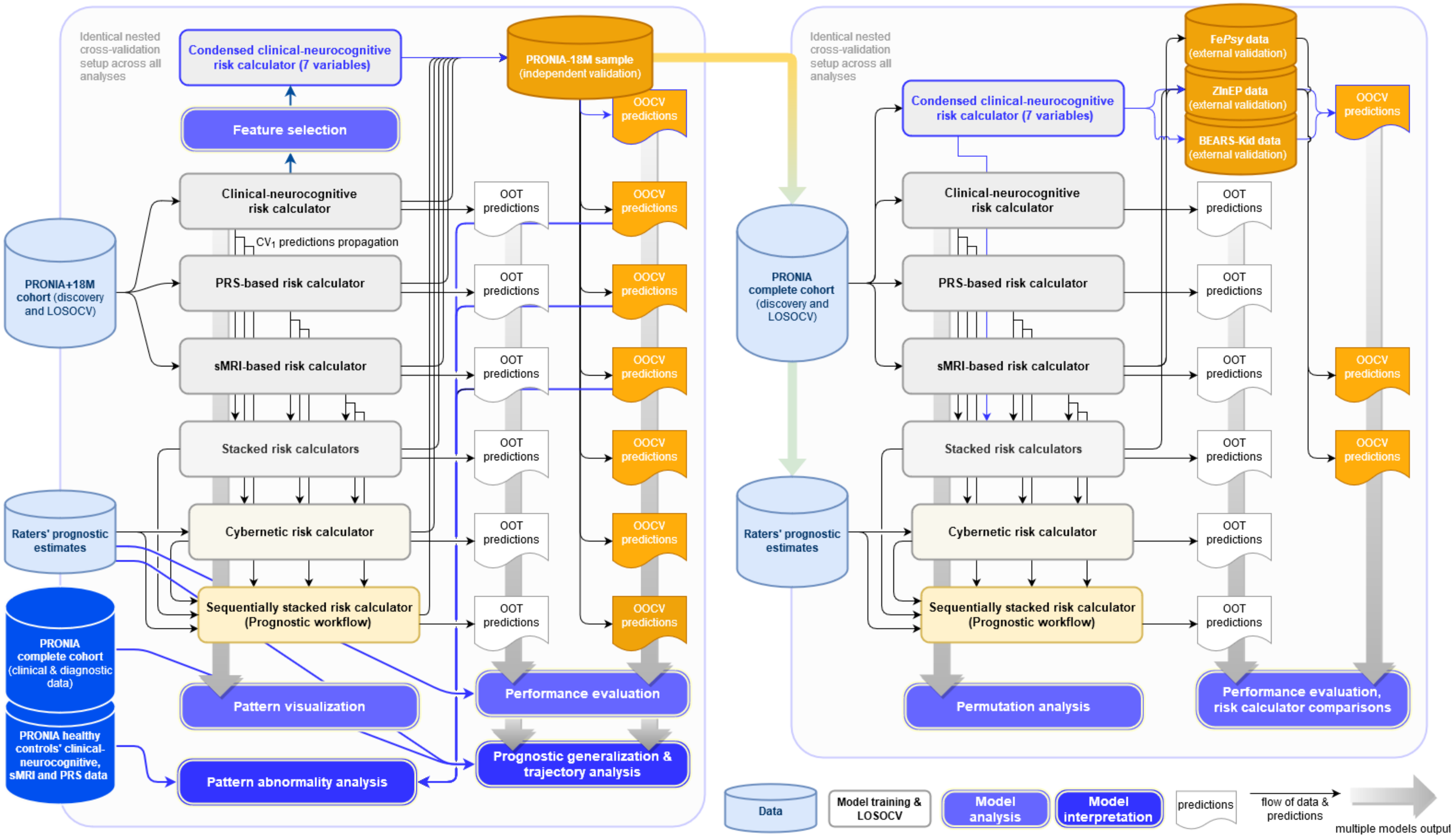

See eMethods for a detailed description of the analysis process. Left panel depicts discovery analyses, right panel depicts retraining for permutation analysis, risk calculator comparisons and external validation of discovery models (reduced clinical-neurocognitive model, sMRI-based model and a stacked risk calculator combining these two models). **Abbreviations:** *LOSOCV* Leave-one-site-out cross-validation, *OOCV* Out-of-cross-validation predictions, *OOT* Out-of-training predictions, *PRONIA+18M cohort* Multi-modal datasets of PRONIA patients who transitioned to psychosis or were followed for at least 18 months, *PRONIA-18M cohort* Multi-modal datasets of PRONIA patients who were followed for less than 18 months, *PRONIA complete cohort* Combined multi-modal database comprising the data of patients in the PRONIA+18M and PRONIA-18M cohorts.

© 2020 Koutsouleris N et al. *JAMA Psychiatry.*

eFigure 3. Experimental Design of the Machine Learning Pipelines Used to Train and Cross-validate the Unimodal and Stacked Risk Calculators

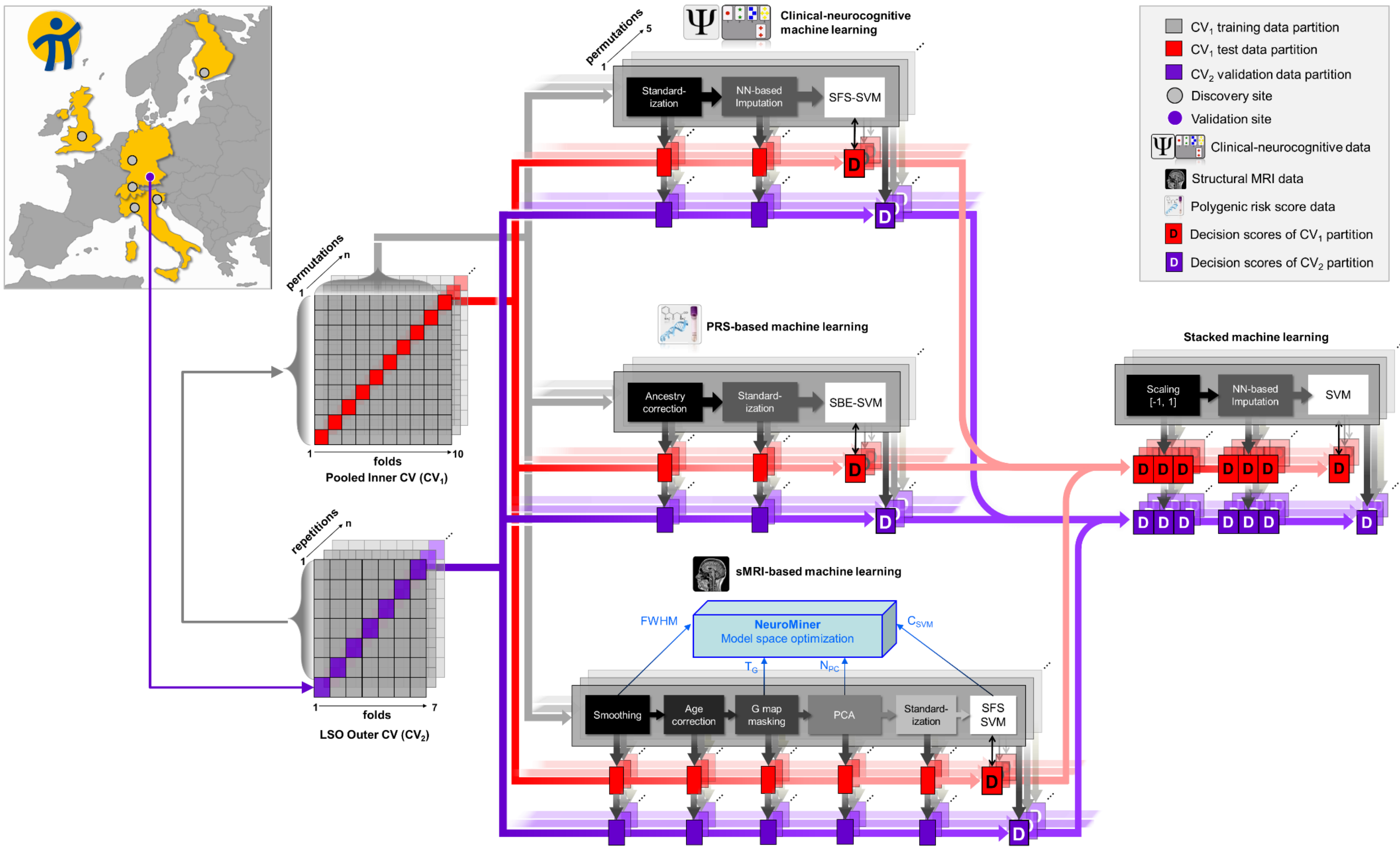

Please see eMethods for further details. **Abbreviations:** *CV* Cross-validation, C_{SVM} Regularization hyperparameter of the Support Vector Machine (*SVM*), *FWHM* full-width-at-half-maximum smoothing kernel, *LSO* Leave-site-out *NN* Nearest neighbor, *PCA* Principal component analysis returning a given number of principal components (N_{PC}), *PRS* Polygenic Risk Scores, *SBE* Sequential backward elimination, *SFS* Sequential forward search, *sMRI* structural Magnetic Resonance Imaging, T_G voxel masking threshold in the generalization theory map (*G* map).

© 2020 Koutsouleris N et al. *JAMA Psychiatry.*

eFigure 4. Schematic Representation of the NeuroMiner Model Optimization Process Used to Train the Structural MRI Predictors

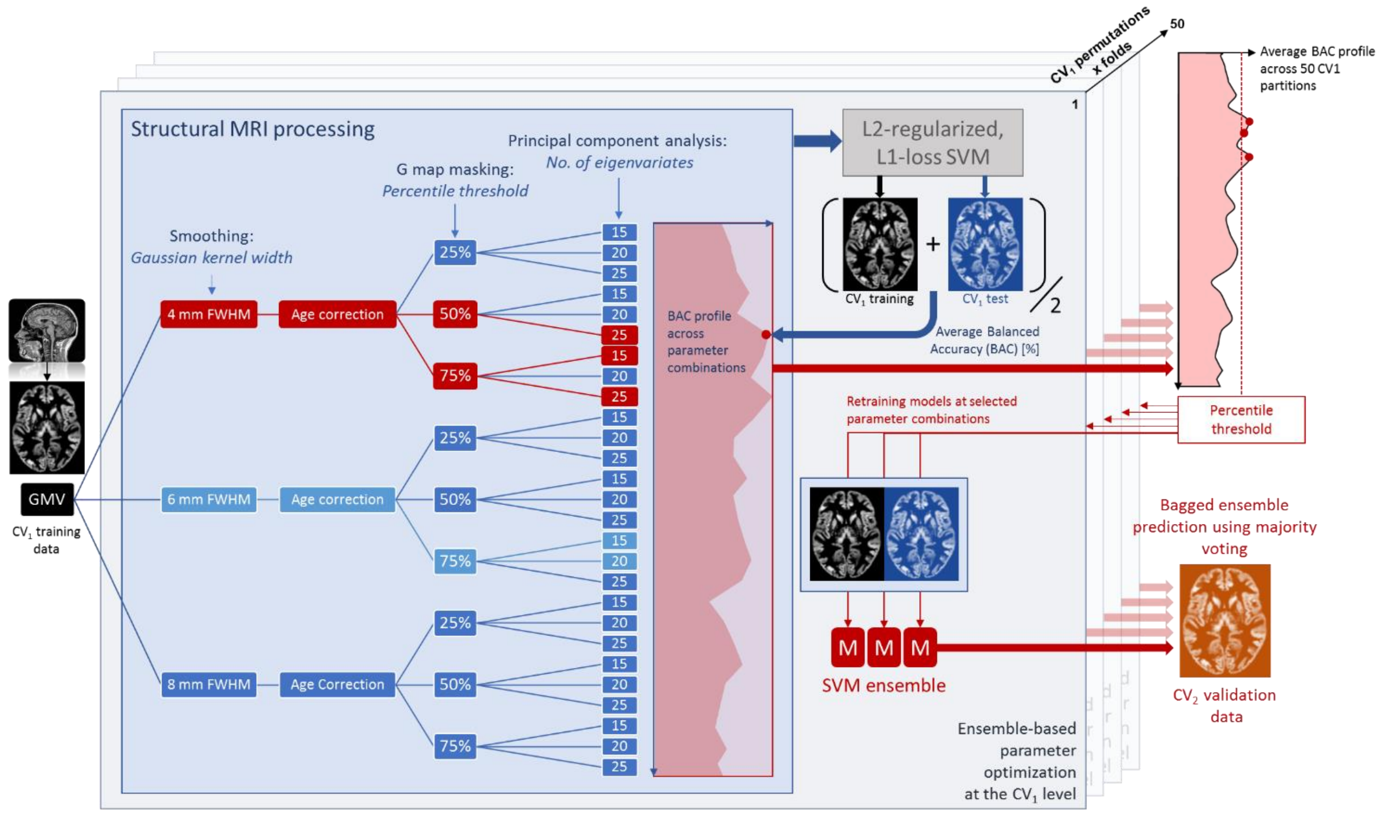

The preprocessing pipeline involved the optimization of three hyperparameters (Gaussian kernel width [FWHM], percentile threshold of G map masking [T_G], number of Principal Components generated by Principal Component Analysis [N_{PC}]). In addition, the optimization of the SVM slack parameter C_{SVM} and the greedy forward search of optimally predictive eigenvariates was integrated in this model selection process (not shown in the figure for the sake of simplicity). A model performance profile across these 3 (FWHM) x 3 (T_G) x 3 (N_{PC}) x 5 (C_{SVM}) = 135 parameter combinations was computed by applying each model (M) to the respective CV_1 training and test data and calculating the average BAC in given partition. To reduce the risk of overfitting, the top three of the most predictive models were selected from the CV_1 test performance profile, which was computed by averaging across the 5 x 10 CV_1 test data partitions. This SVM model ensemble (comprising 3 * 5 CV_1 permutations * CV_1 10 folds = 150 SVM models) was then applied to the CV_2 validation data provided by the respective held-back site.

© 2020 Koutsouleris N et al. *JAMA Psychiatry.*

eFigure 5. Predictive Signature Underlying the sMRI-Based Risk Calculator

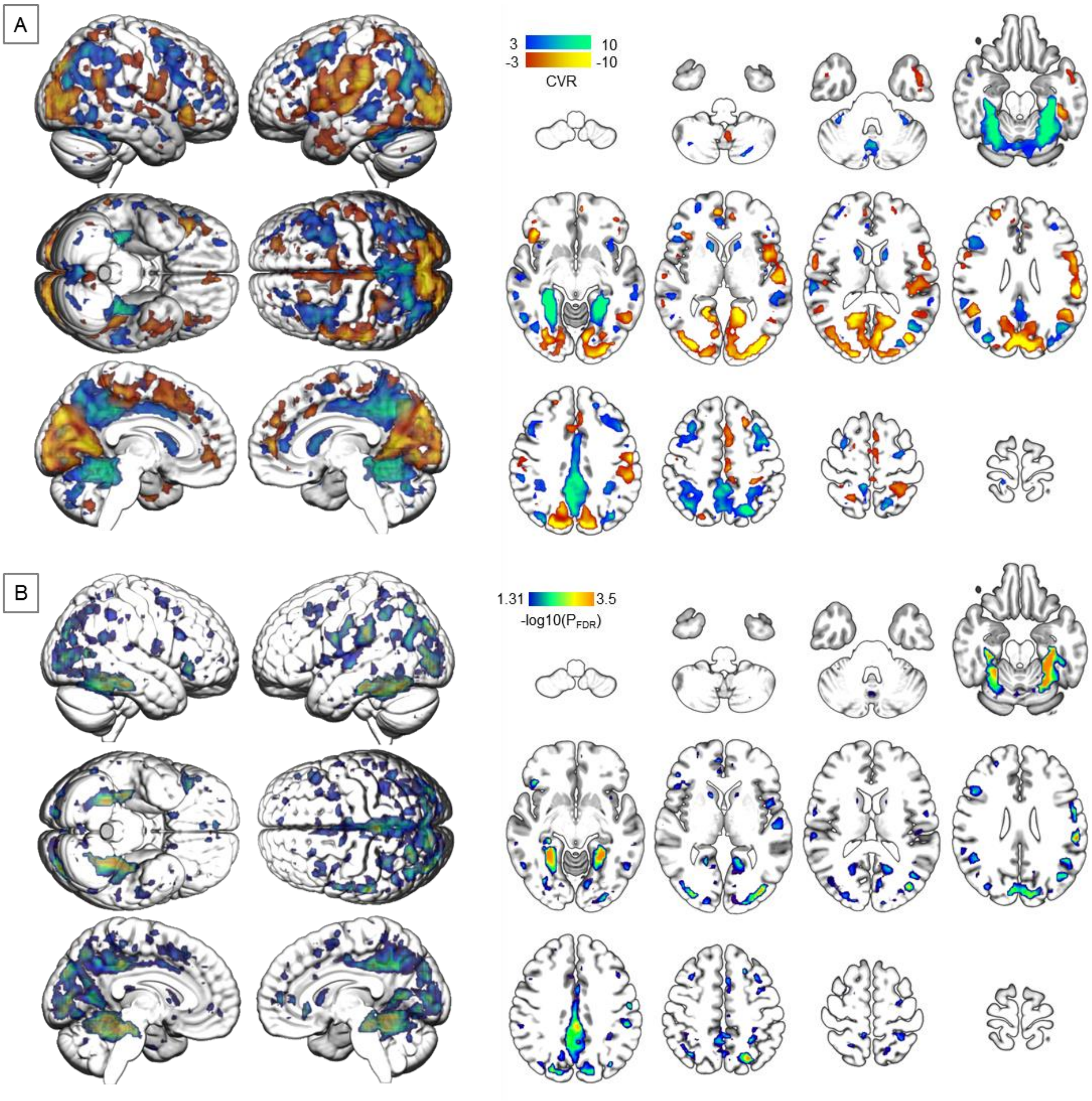

A: the reliability of predictive pattern elements is visualized by means of cross-validation ratio mapping. **B:** Sign-based consistency mapping depicts patterns elements that significantly contributed to the risk calculator's predictive pattern. Cool colors indicate voxels with increased brain volumes in PT patients while warm colors represent increased brain volume in NT patients. The **eMethods** in the Supplementary Material provide a detailed description of both visualization methods.

© 2020 Koutsouleris N et al. *JAMA Psychiatry.*

eFigure 6. Comparison of Predictive Performance of Expert-Based, Unimodal, Stacked, Cybernetic and Sequentially Stacked Risk Calculators Trained and Cross-validated Using the PRONIA-18M and Complete PRONIA Cohorts

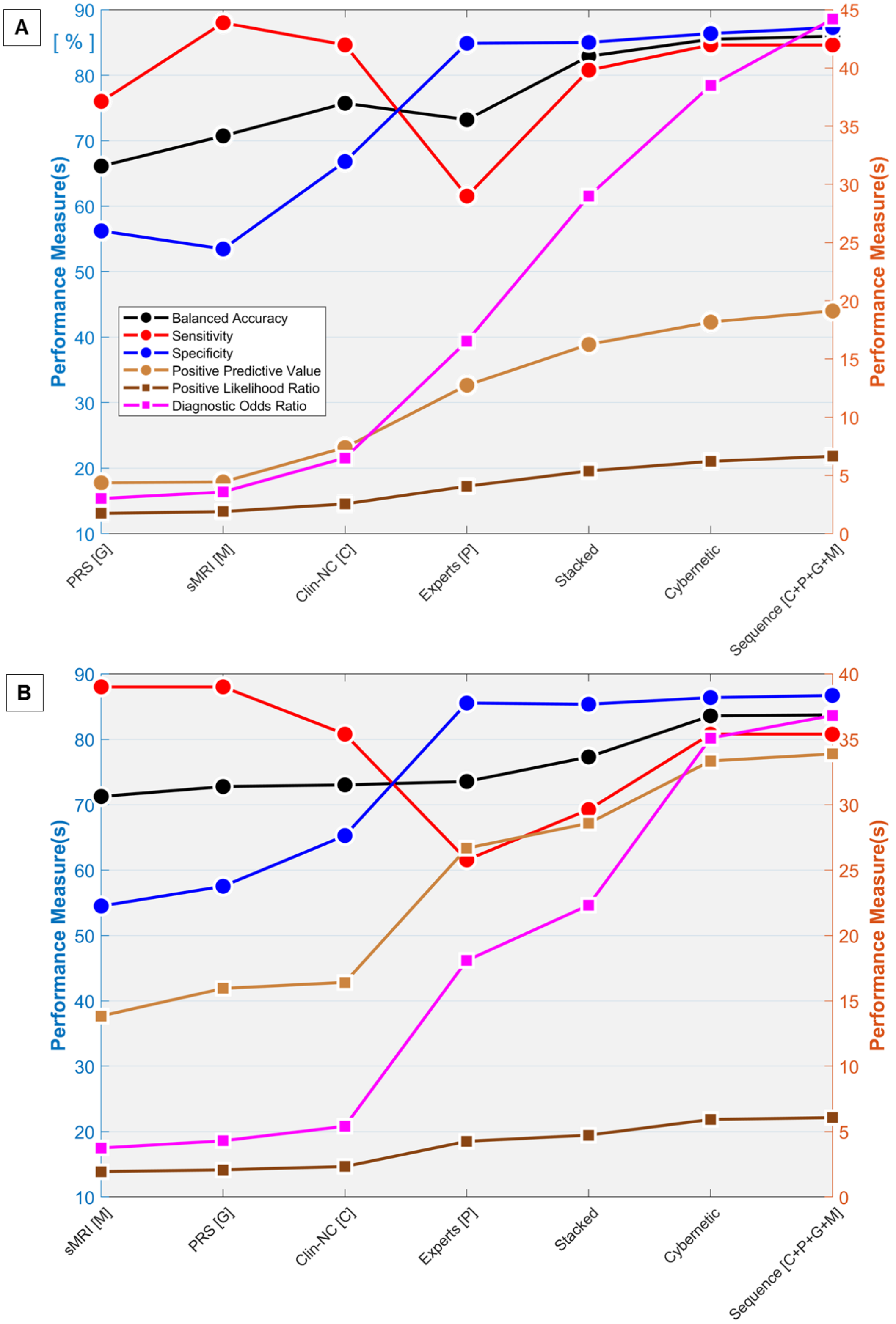

Analyses were ranked from lowest (left) to highest (right) performance by computing the mean across sensitivity, specificity, balanced accuracy, positive predictive value, positive likelihood ratio and diagnostic odds ratio in each analysis and sorting analyses using this measure. Squared marker graphs map to the right, circled markers to the left y axis. **Abbreviations:** *Clin-NC* Clinical-neurocognitive risk calculator, *PRS* PRS-based risk calculator, *sMRI* sMRI-based risk calculator, *Stacked* Stacked-risk calculator.

© 2020 Koutsouleris N et al. *JAMA Psychiatry.*

eFigure 7. Comparison of standardized Clinical-Neurocognitive Variables Between Healthy Volunteers and CHR/ROD Patients Who Were Labeled With a Transition or Nontransition to Psychosis by the Clinical-Neurocognitive Risk Calculator

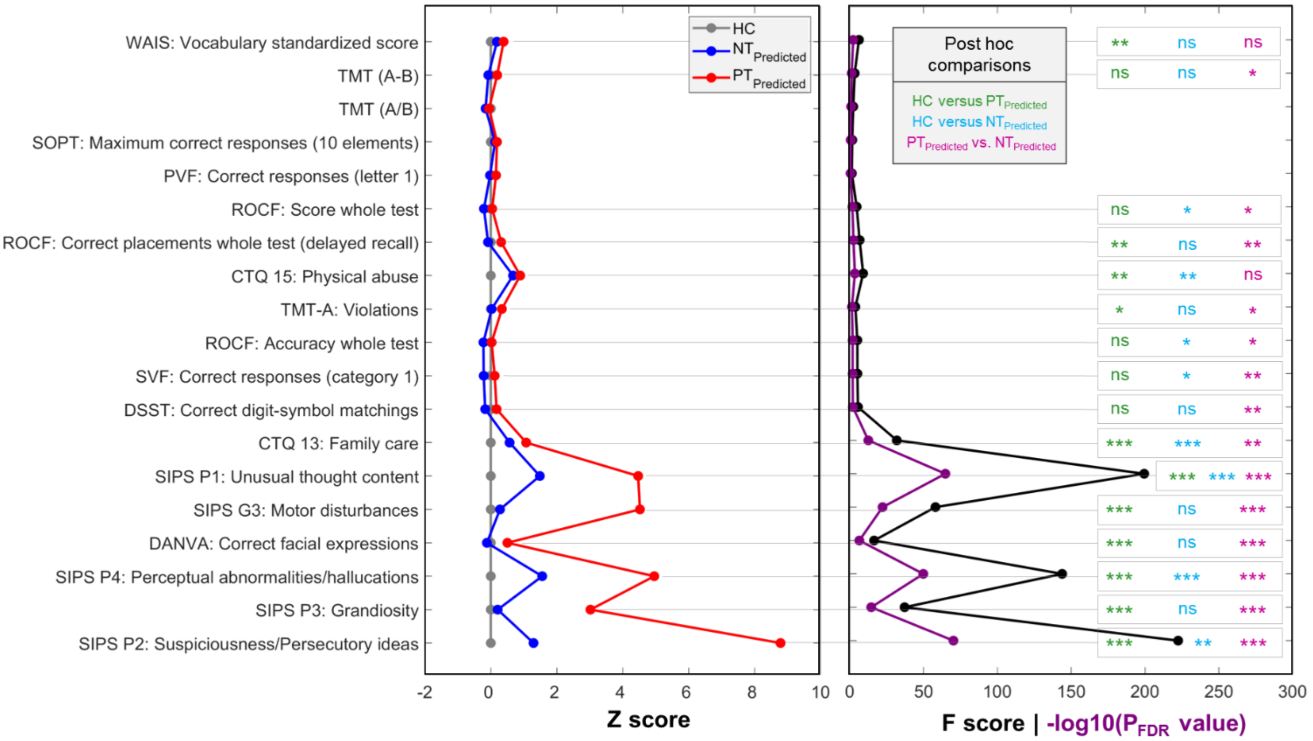

Analysis of group differences in variables which were reliably predictive of PT in the clinical-neurocognitive risk signature (see **Figure 1**, main manuscript). See **section 1.5.6** for methodological details. Z score profiles were visually compared between HC (gray line), predicted PT (red line) and predicted NT patients (blue line). Latter two groups were produced by the prognostic assignments of the clinical-neurocognitive risk calculator. The three groups were statistically compared using Multivariate Analysis of Variance (MANOVA) producing a significant omnibus test (Wilk's λ=0.282; *F*=20.89; *P*<.001). The right side of the figure shows the results of the between-group ANOVAs which followed the significant omnibus test (*F* scores, black line; -log10(P_{FDR} value) scores, purple line). Pairwise post hoc analyses were carried out for significant ANOVAs and obtained *P* values were adjusted for multiple comparisons using Tukey's HSD method.

© 2020 Koutsouleris N et al. *JAMA Psychiatry.*

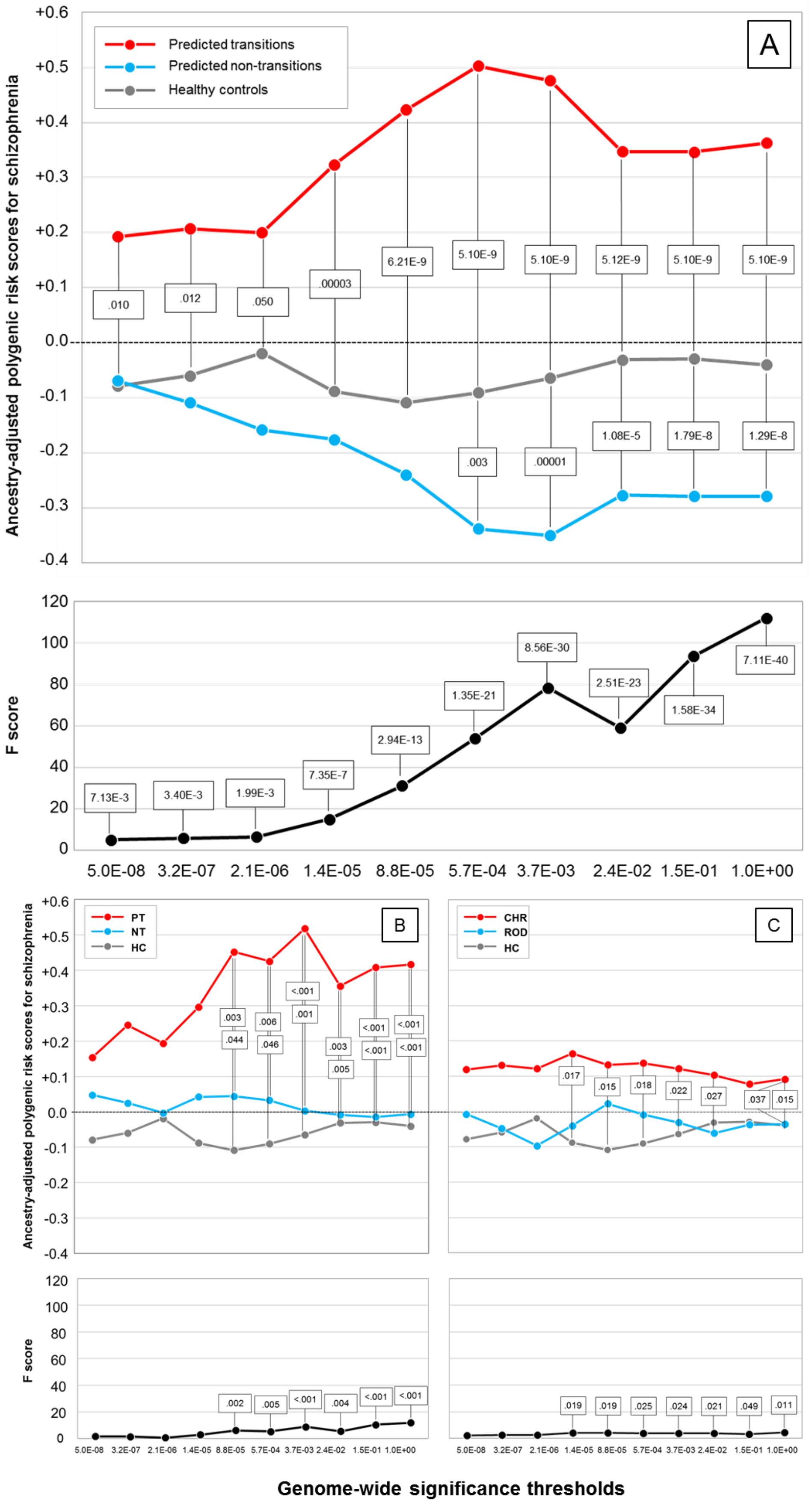

eFigure 8. Comparison of Polygenic Risk Scores (PRS) Between Healthy Volunteers and CHR/ROD Patients Who Were Labeled With a Transition or Nontransition to Psychosis by the PRS-Based Risk Calculator

Group comparison in the polygenic risk scores (PRS) between HC (gray line), predicted transition (red line) vs. predicted non-transition patients (blue line). Latter two groups were produced by the PRS-based risk calculator (**A**). Similar comparisons were conducted between observed outcomes (PT, NT) and HC (**B**), as well as between CHR, ROD, and HC groups (**C**). The lower graph in each plot shows the F scores (black line) and respective *P* values of a Multivariate Analysis of Variance (MANOVA) that was carried out to assess main effects of group across 10 genome-wide significance thresholds. The omnibus test was significant at *P*<.001 (Wilk's λ=0.598; *F*=14.27). Post hoc analyses were performed to compare both predicted outcome groups to the healthy volunteers and obtained *P* values were corrected for multiple comparisons using Tukey's HSD method. Like **eFigure 9**, the analysis showed that both predicted outcome groups differ significantly from the HC population. In similar MANOVAs performed in the observed outcome group vs. HC (**B**) and the study groups vs. HC (**C**) we did not observe reduced polygenic risk in non-transition cases or ROD patients compared with the HC participants.

© 2020 Koutsouleris N et al. *JAMA Psychiatry.*

eFigure 9. Univariate Volumetric Comparisons Between Healthy Volunteers and Patients Labeled With a Transition or Nontransition to Psychosis by the sMRI-Based Risk Calculator

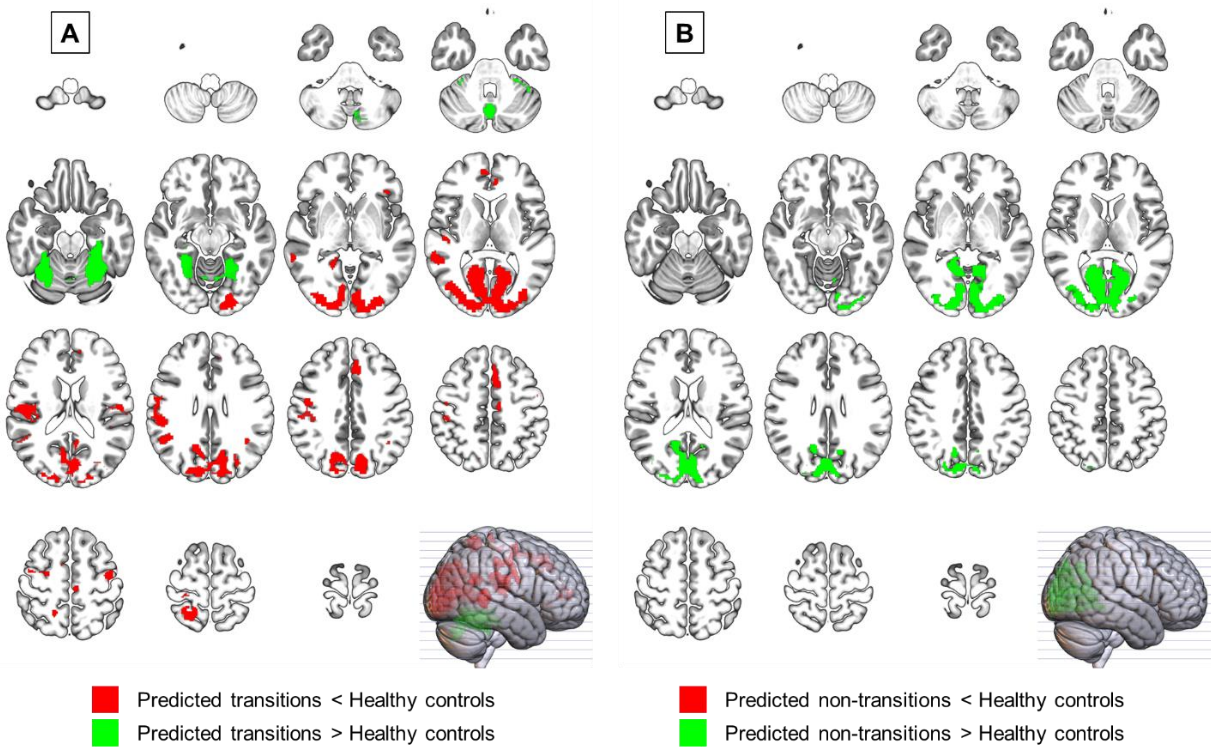

All analyses were carried out in SPM12 (MATLAB 2019b). Structural images were smoothed with an 8 mm Full-width-at-half-maximum Gaussian kernel before entering a non-parametric analysis of pairwise group differences performed using the Threshold-Free Cluster Enhancement toolbox for SPM12 (http://www.neuro.uni-jena.de/tfce/). To assess the significance of differences 5000 permutations of group memberships were performed and respective null distributions of the TFCE scores were constructed. The figure shows brain regions where differences between predicted outcome groups and healthy controls were significant at α=0.05 after alpha correction using the False-Discovery-Rate (FDR).

© 2020 Koutsouleris N et al. *JAMA Psychiatry.*

eFigure 10. Image Quality Assessment of T1-Weighted Images Analyzed in the Study Using the Quality Assessment Tools of the CAT12 Toolbox

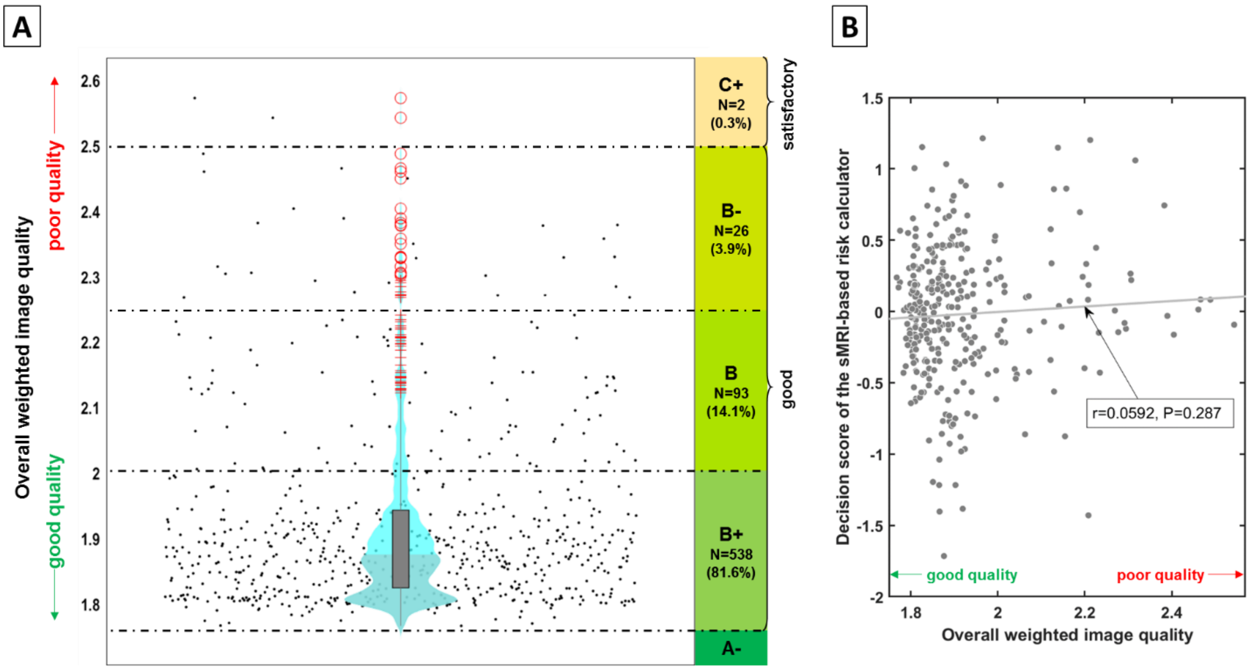

A: Violin plot and scoring showing the distribution of image quality in the current study. **B:** Correlation analysis between the overall weighted image quality and the decision scores of the sMRI-based risk calculator.

© 2020 Koutsouleris N et al. *JAMA Psychiatry.*

eFigure 11. Interaction Analysis Assessing the Effects of the Number of Examinations Available and the Longest Interval Duration on the Predictions of Four Different Risk Calculators

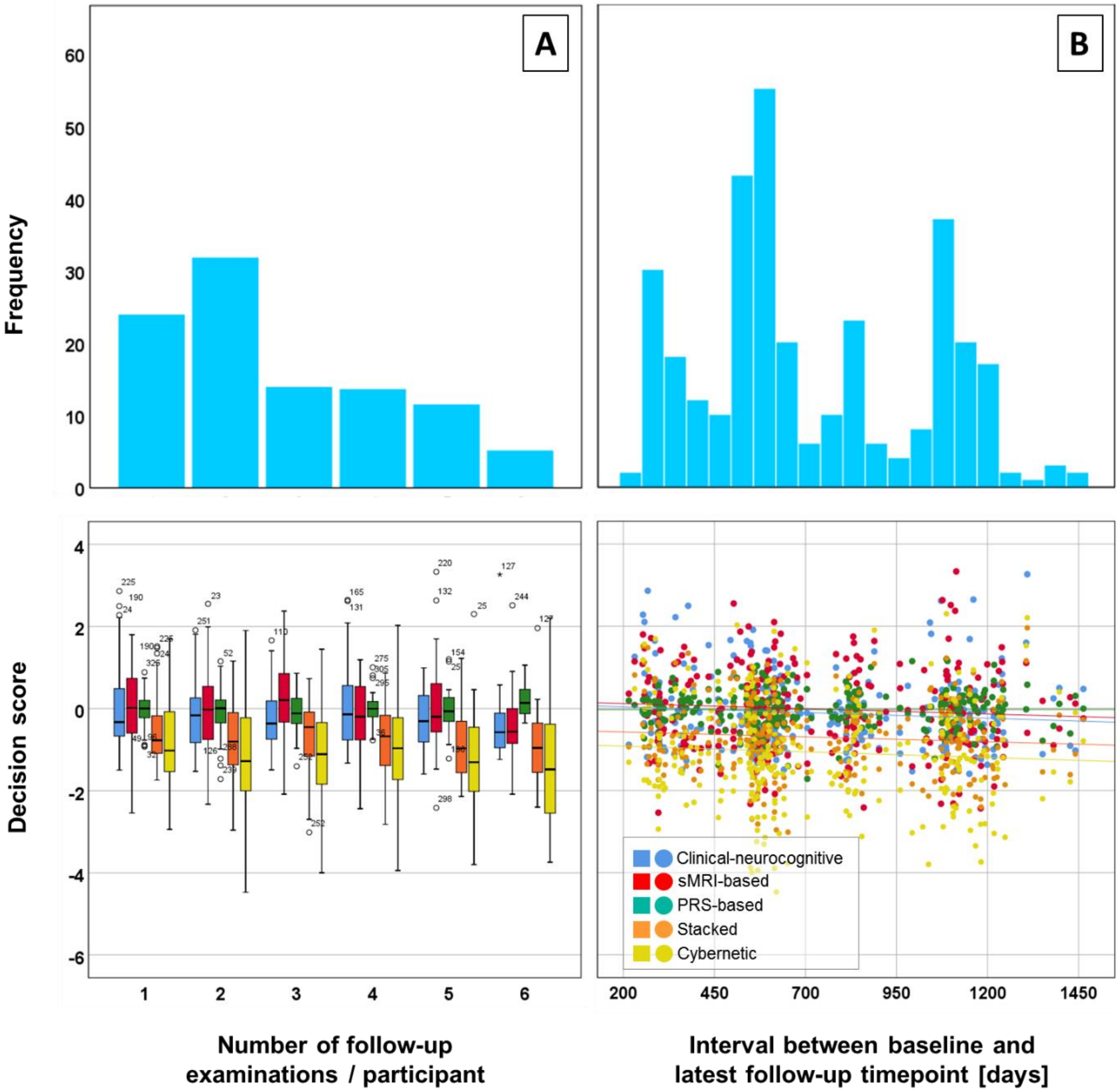

Interaction analysis assessing the effects of the number of examinations available (A) and the longest interval duration (B) on the predictions of unimodal, stacked and cybernetic risk calculators. Top: histogram analyses of the number of follow-up time points and longest follow-up intervals. **Bottom:** Box and scatter plots of the respective variables and the decision scores of the four risk calculators (Clinical-neurocognitive, sMRI-based, PRS-based, cybernetic models) trained to predict transition to psychosis. The cybernetic risk calculator refers to the stacked model integrating all algorithmic estimates with raters' predictions. See **eTable 8** for the respective statistical analysis of moderating effects of follow-up frequency and follow-up duration on decisions scores.

© 2020 Koutsouleris N et al. *JAMA Psychiatry.*

eFigure 12. Statistical Analysis of Prognostic Performance Effects Induced by the ROD Depletion and Substitution Strategies in the Three Unimodal Risk Calculators and the Stacked Model

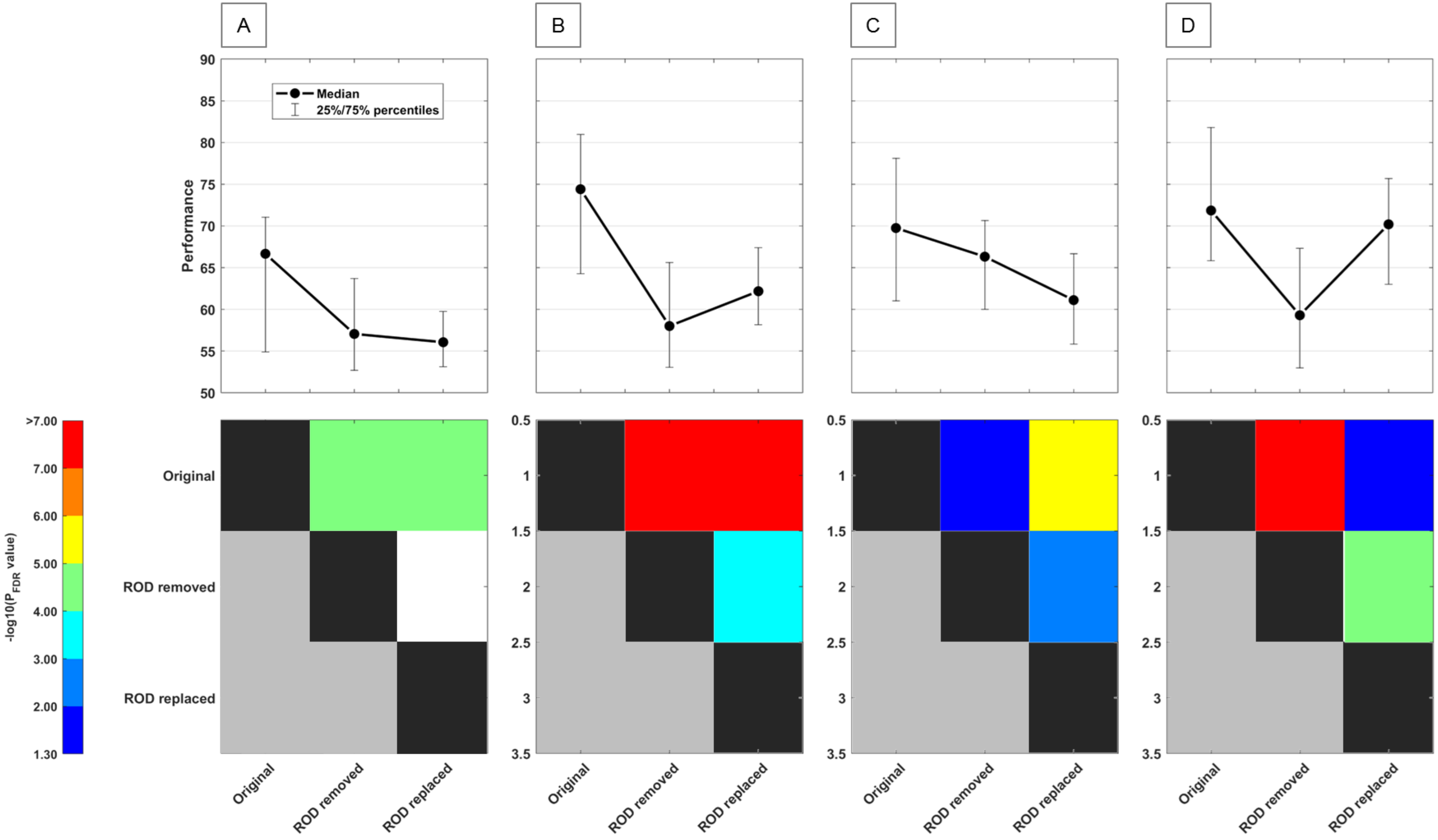

A	B	C	D
$W_{2,138}$=11.7; *P*<.001	$W_{2,138}$=44.9; *P*<.001	$W_{2,138}$=13.5; *P*<.001	$W_{2,138}$=20.3; *P*<.001

Statistical analysis of CHR-specific prognostic performance changes of clinical-neurocognitive (**A**), PRS-based (**B**), sMRI-based (**C**) and stacked risk calculators (**D**) induced by removing ROD patients from the patient population (and learning predictive signatures only in CHR patients) or by replacing ROD patients with healthy controls. Performance was defined as the balanced accuracy of the ensemble models in predicting PT in the respective CV_2 test data partitions of the LOSOCV cycle. The statistical analysis revealed that ROD patients contributed significantly to the CHR-specific prognostic performance across all unimodal and stacked risk models. Methodological descriptions are provided in **section 1.5.7**

© 2020 Koutsouleris N et al. *JAMA Psychiatry.*

eFigure 13. Prognostic Stratification Effects of Raters' Predictions, Unimodal and Cybernetic Risk Calculators on the Trajectories of CHR Syndromes and Functioning as Determined by Nonnegative Matrix Factorization and Linear Mixed-Effects Modelling

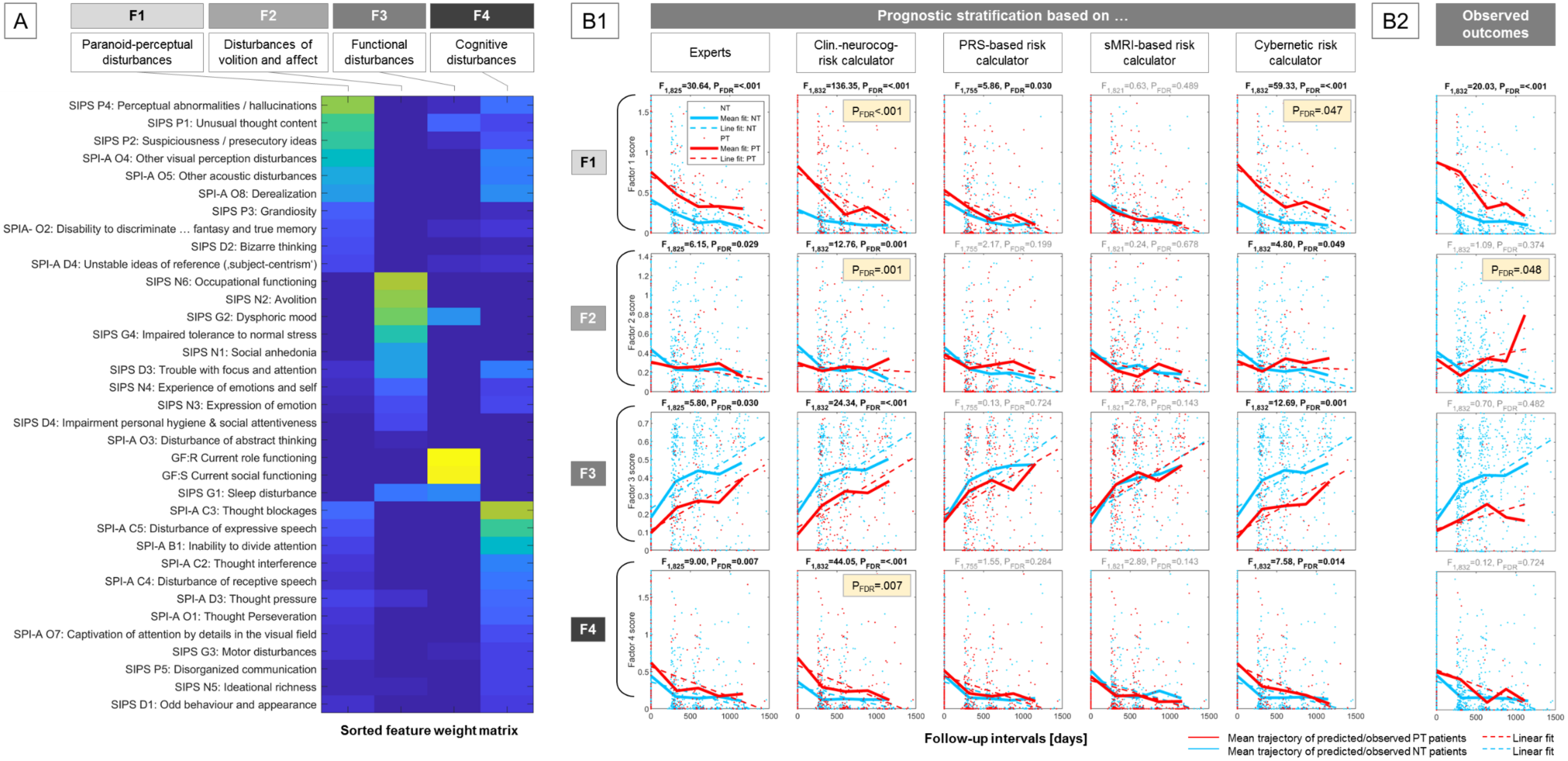

First, psychopathological and functioning baseline symptoms were projected to four factors using non-negative matrix factorization (sorted factor loadings in **A**). The respective variables recorded at the T1, T2, T3 and T4 time points were projected into this NNMF model and obtained factor scores were clustered at the average follow-up time points of the study. Linear mixed-effects models were used to compare main and slope differences between trajectories of prognostic assignments (**B1**) or observed outcomes (**B2**). Group-level trajectories shown in **B1** and **B2** have been computed by averaging subject-level factor scores at the average follow-up time points. In addition, linear fits were depicted in each factor-model plot. Significant main effects were highlighted in bold. In case of significant slope differences, the *P* value was provided in a cream-colored box. See **section 1.5.8** for methodological details.

© 2020 Koutsouleris N et al. *JAMA Psychiatry.*

eFigure 14. Detailed Analysis of the Optimal Sequential Prediction Algorithm

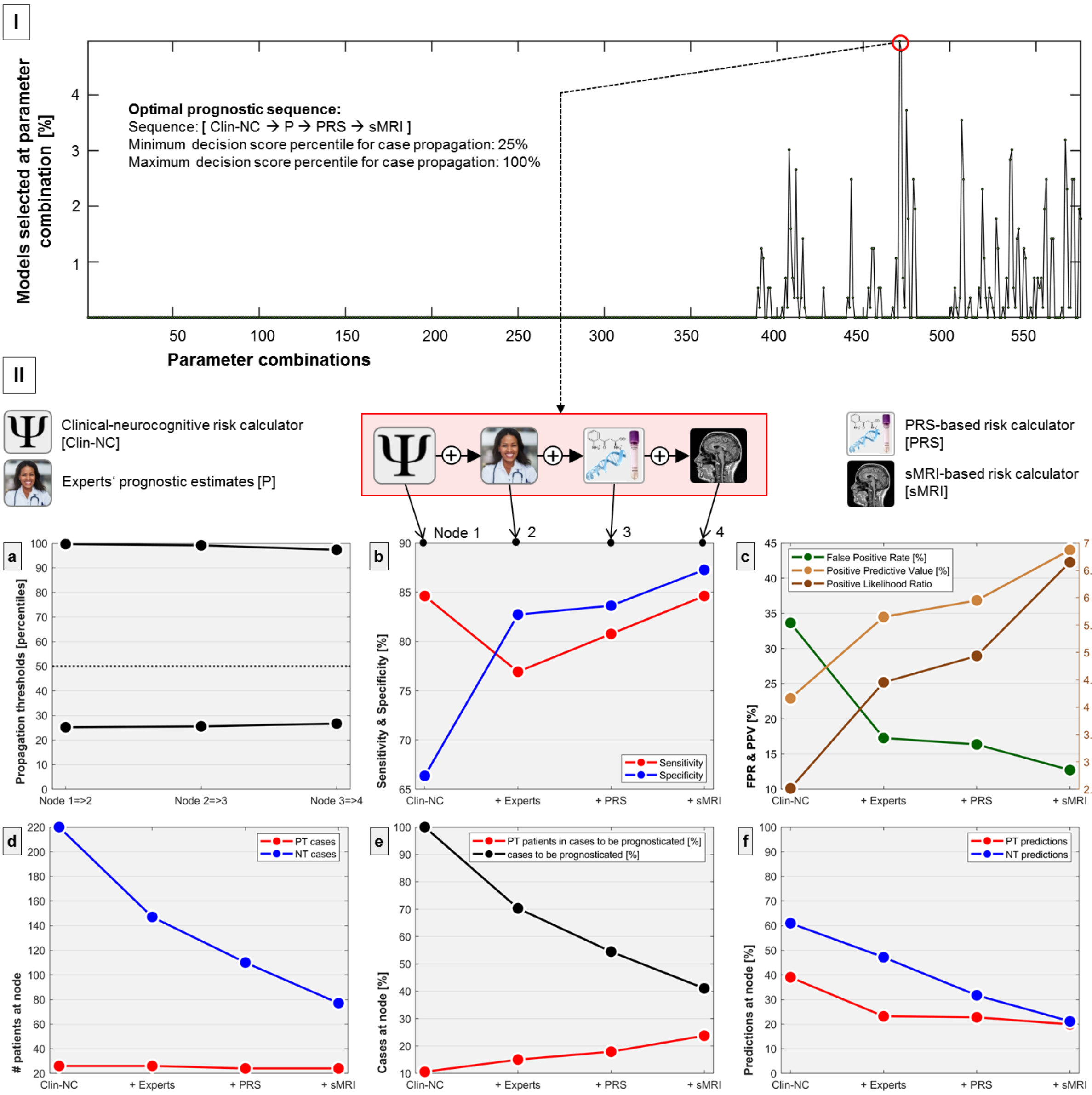

I: Nested leave-site-out cross-validation was used to identify the optimal sequential stacking algorithm as defined by the most frequently selected sequence parameter combination across 576 possible combinations (64 sequences × 3 lower × 3 upper case propagation percentiles; for a description of prognostic sequences see **eTable 6**). **II:** The optimal prognostic sequence started with the clinical-neurocognitive risk calculator (Clin-NC). Cases within the propagation thesholds **(a)** were forwarded to a cybernetic risk calculator which analyzed the risk scores of the Clin-NC model together with the raters' prognostic estimates. In turn, cases were propagated to cybernetic models analyzing additionally the PRS-based and sMRI-based risk calculators' scores. The out-of-training (OOT) prediction performance changes were measured at each prognostication node using **(b)** the false-positive rate, positive predictive value (left y axis) and positive likelihood ratio (right y axis), as well as **(c)** sensitivity and specificity. Furthermore, figures **d**, **e**, and **f** show changes in the absolute numbers of PT and NT cases, PT cases in relation to the percentage of remaining cases to be prognosticated at given node, and percentages of predicted outcome labels relative to the PRONIA plus 18M sample, respectively.

© 2020 Koutsouleris N et al. *JAMA Psychiatry.*

eFigure 15. Regularization of the Prognostic Workflow for Different Levels of Examination Sparsity and Analysis of Effects of Regularization on Prognostic Performance

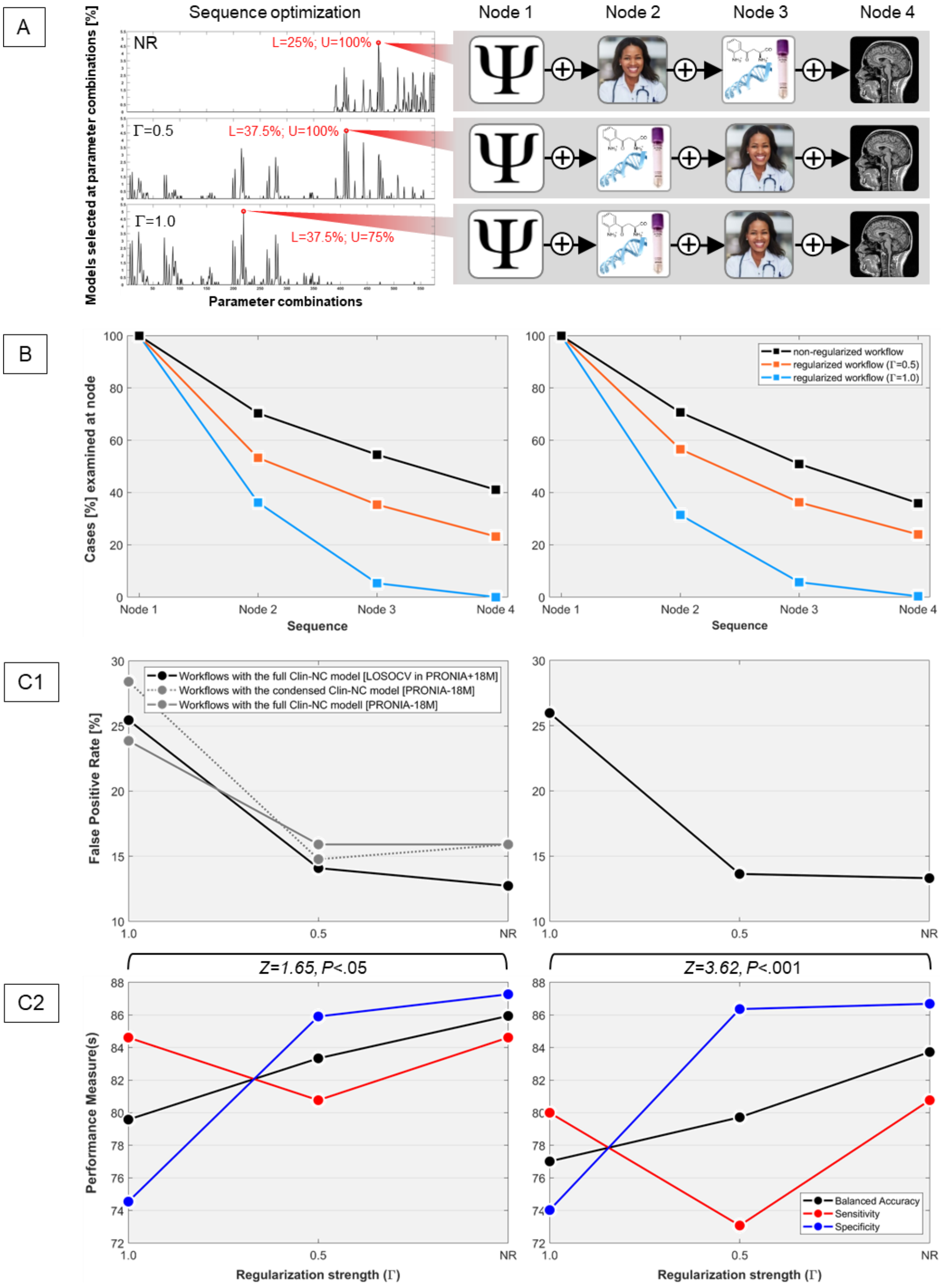

A: Description of prognostic sequences obtained at two parsimony regularization strengths (Γ=[0.5, 1.0]) vs. the non-regularized (NR) workflow, including model selection frequency plots for each regularization strength (left) and depictions of the optimal sequences (right) at each model selection maximum (red circle; U/L=maximum /minimum case propagation percentiles). Examination icons are explained in **eFigure 14**. **B:** Analysis of node utilization frequencies in the three sequences, illustrating that higher regularization strength reduces the case propagation likelihood to the final examination step (sMRI). **C1:** False positive rates at Γ=1.0, Γ=0.5, and NR in the PRONIA+18M sample (black line) and the PRONIA-18M non-transition cases (grey lines). Dotted grey line describes the false positive rate of a prognostic workflow containing the condensed clinical-neurocognitive instead of the full model. **C2:** Workflow sensitivity, specificity and balanced accuracy at Γ=1.0, Γ=0.5, and NR in the PRONIA+18M sample (left) and complete PRONIA sample (right). Based on the Wilcoxon signed rank test, significant differences in balanced accuracy were found between NR and Γ=1.0, but not between NR and Γ=0.5.

© 2020 Koutsouleris N et al. *JAMA Psychiatry.*

eFigure 16. Comparison of Clinical-Neurocognitive Signatures Used by the Prognostic Risk Calculator and the Differential Diagnostic Classifier (CHR vs ROD)

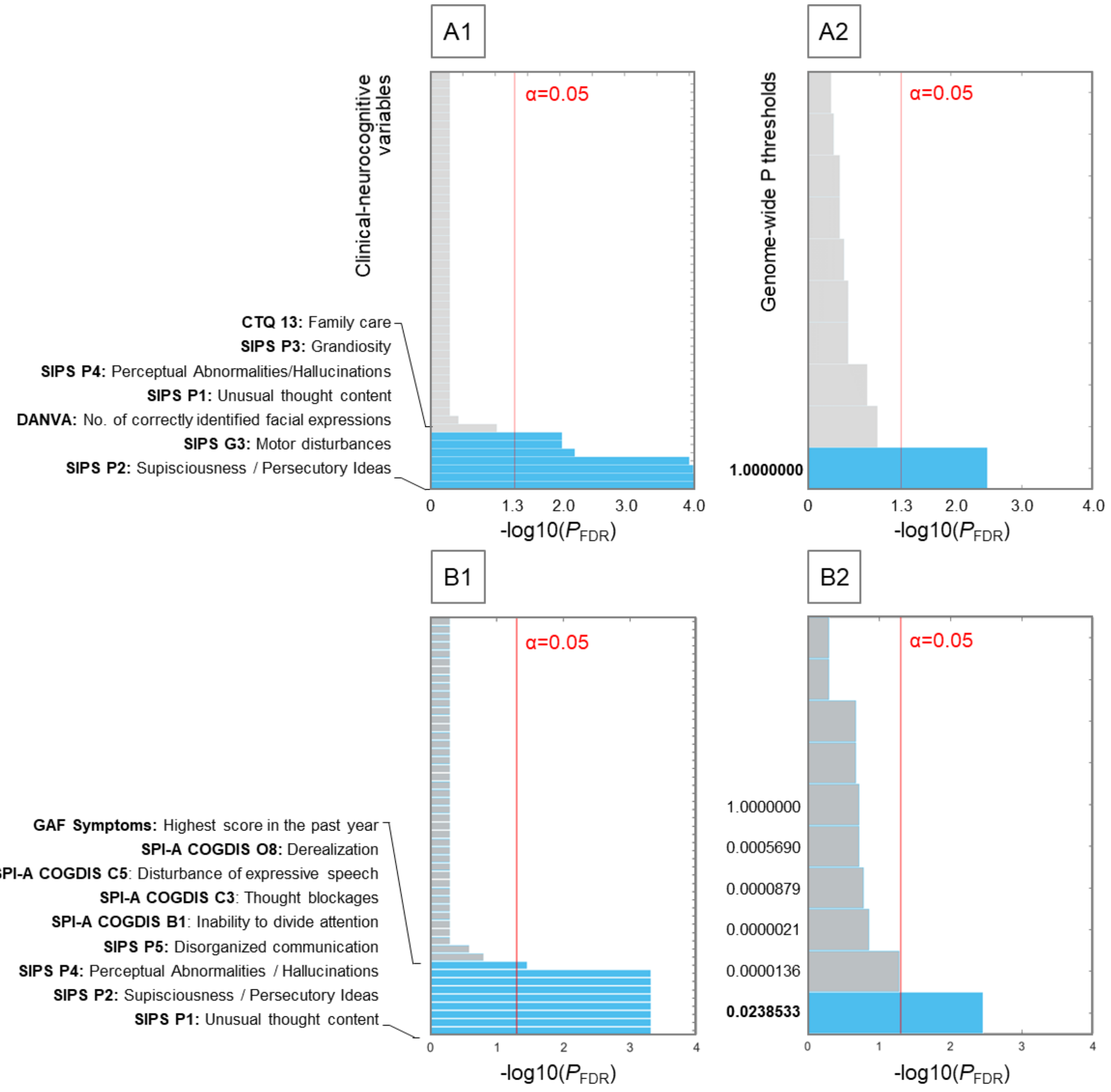

Comparison of clinical-neurocognitive (**1**) and polygenic risk signatures (**2**) used by the risk calculators (**A**) and the differential diagnostic classifiers (CHR vs. ROD, **B**). Significance was determined using sign-based consistency mapping as described in **section 1.5.5**. Abbreviations of clinical-neurocognitive features are detailed in **eTable 5**.

© 2020 Koutsouleris N et al. *JAMA Psychiatry.*

eReferences

1. Fusar-Poli, P. *et al.* Prevention of Psychosis: Advances in Detection, Prognosis and Intervention. *JAMA Psychiatry* **77**, 1–11 (2020).
2. Cannon, T. D. *et al.* An individualized risk calculator for research in prodromal psychosis. *American Journal of Psychiatry* **173**, 980–988 (2016).
3. McGlashan, T., Walsh, B. & Woods, S. *The psychosis-risk syndrome: handbook for diagnosis and follow-up*. (Oxford University Press: 2010).
4. Schultze-Lutter, F., Addington, J., Ruhrmann, S. & Klosterkötter, J. Schizophrenia Proneness Instrument, Adult Version (SPI-A). (2007).
5. Pedersen, G., Hagtvet, K. A. & Karterud, S. Generalizability studies of the Global Assessment of Functioning–Split version. *Comprehensive Psychiatry* **48**, 88–94 (2007).
6. Cornblatt, B. A. *et al.* Preliminary findings for two new measures of social and role functioning in the prodromal phase of schizophrenia. *Schizophrenia Bulletin* **33**, 688–702 (2007).
7. Bernstein, D. & Fink, L. CTQ: Childhood Trauma Questionnaire: a retrospective self-report. *San Antonio, TX: Psychological Corp* (1998).
8. Wechsler, D. *Wechsler Adult Intelligence Scale*. (Psychological Coorperation: San Antonio, TX, 1997).
9. Keefe, R. S. E. *et al.* Comparative effect of atypical and conventional antipsychotic drugs on neurocognition in first-episode psychosis: a randomized, double-blind trial of olanzapine versus low doses of haloperidol. *American Journal of Psychiatry* **161**, 985–995 (2004).
10. Reitan, R. M. TMT, Trail Making Test A & B. (1992).
11. Petrides, M. Impairments on nonspatial self-ordered and externally ordered working memory tasks after lesions of the mid-dorsal part of the lateral frontal cortex in the monkey. *Journal of Neuroscience* **15**, 359–375 (1995).
12. Rey, A. *L'examen psychologique dans les encéphalopathies traumatiques*. (1943).
13. Joyce, E. M., Collinson, S. L. & Crichton, P. Verbal fluency in schizophrenia: relationship with executive function, semantic memory and clinical alogia. *Psychological Medicine* **26**, 39–49 (1996).
14. Arbuthnott, K. & Frank, J. Trail making test, part B as a measure of executive control: validation using a set-switching paradigm. *Journal of Clinical and Experimental Neuropsychology* **22**, 518–528 (2000).
15. Nowicki, S. & Duke, M. P. Individual differences in the nonverbal communication of affect: The Diagnostic Analysis of Nonverbal Accuracy Scale. *Journal of Nonverbal behavior* **18**, 9–35 (1994).
16. Neuroimaging, W. T. C. for Statistical Parametric Mapping 12. (2014).at <http://www.fil.ion.ucl.ac.uk/spm/software/spm12/>
17. Manjón, J. V. *et al.* Robust MRI brain tissue parameter estimation by multistage outlier rejection. *Magnetic Resonance in Medicine* **59**, 866–873 (2008).
18. Rajapakse, J. C., Giedd, J. N. & Rapoport, J. L. Statistical approach to segmentation of single-channel cerebral MR images. *IEEE Trans. Med. Imaging* **16**, 176–186 (1997).
19. Purcell, S. *et al.* PLINK: a tool set for whole-genome association and population-based linkage analyses. *The American Journal of Human Genetics* **81**, 559–575 (2007).
20. Wolpert, D. H. Stacked generalization. *Neural Networks* **5**, 241–259 (1992).
21. Steyerberg, E. W. & Harrell, F. E. Prediction models need appropriate internal, internal–external, and external validation. *Journal of clinical epidemiology* **69**, 245–247 (2016).
22. Golland, P. & Fischl, B. Permutation tests for classification: towards statistical significance in image-based studies. *Inf Process Med. Imaging* **18**, 330–341 (2003).
23. Theodoridou, A. *et al.* Early Recognition of High Risk of Bipolar Disorder and Psychosis: An Overview of the ZInEP "Early Recognition" Study. *Frontiers in public health* **2**, 166 (2014).
24. Riecher-Rössler, A. *et al.* The Basel early-detection-of-psychosis (FEPSY)-study–design and preliminary results. *Acta Psychiatrica Scandinavica* **115**, 114–125 (2007).

© 2020 Koutsouleris N et al. *JAMA Psychiatry.*

25. Schultze-Lutter, F. *et al.* Clinical high risk for psychosis in children and adolescents: findings of the Bi-national Evaluation of At-Risk Symptoms in children and adolescents (BEARS-Kid) study. **269**, S14–S15
26. Koutsouleris, N. *et al.* Prediction Models of Functional Outcomes for Individuals in the Clinical High-Risk State for Psychosis or With Recent-Onset Depression: A Multimodal, Multisite Machine Learning Analysis. *JAMA Psychiatry* **75**, 1156–1172 (2018).
27. Brennan, R. L. Generalizability theory and classical test theory. *Applied Measurement in Education* **24**, 1–21 (2010).
28. Hansen, L. K. *et al.* Generalizable patterns in neuroimaging: how many principal components? *Neuroimage* **9**, 534–544 (1999).
29. Saeys, Y., Inza, I. & Larrañaga, P. A review of feature selection techniques in bioinformatics. *Bioinformatics* **23**, 2507–2517 (2007).
30. Fan, R., Chang, K., Hsieh, C., Wang, X. & Lin, C. LIBLINEAR: A Library for Large Linear Classification. (2008).
31. Polikar, R. Ensemble based systems in decision making. *IEEE Circuits and Systems* **6**, 21–45 (2006).
32. Chang, C.-C. & Lin, C.-J. *LIBSVM: a library for support vector machines*. (2001).
33. Gennatas, E. D. *et al.* Expert-augmented machine learning. *Proceedings of the National Academy of Sciences of the United States of America* **117**, 4571–4577 (2020).
34. Editorial The return of cybernetics. doi:https://doi.org/10.1038/s42256-019-0100-x
35. Wiener, N. *The Human Use of Human Beings.* (Houghton Miffliin: Boston,).
36. Boardman, M. & Trappenberg, T. A Heuristic for Free Parameter Optimization with Support Vector Machines. *Proc. Int. Joint Conf. Neural Networks IJCNN '06* 610–617 (2006).doi:10.1109/IJCNN.2006.246739
37. Krishnan, A., Williams, L. J., McIntosh, A. R. & Abdi, H. Partial Least Squares (PLS) methods for neuroimaging: A tutorial and review. *Neuroimage* (2010).doi:10.1016/j.neuroimage.2010.07.034
38. Gaonkar, B. & Davatzikos, C. Deriving statistical significance maps for SVM based image classification and group comparisons. *Med Image Comput Comput Assist Interv* **15**, 723–730 (2012).
39. Noble, W. S. What is a support vector machine? *Nature Biotechnology* **24**, 1565–1567 (2006).
40. Gaonkar, B., T Shinohara, R., Davatzikos, C. & Initiative, A. D. N. Interpreting support vector machine models for multivariate group wise analysis in neuroimaging. *Medical Image Analysis* **24**, 190–204 (2015).
41. Gomez-Verdejo, V., Parrado-Hernandez, E., Tohka, J., Initiative, A. D. N. & others Sign-consistency based variable importance for machine learning in brain imaging. *Neuroinformatics* **17**, 593–609 (2019).
42. Benjamini, Y. & Hochberg, Y. Controlling the false discovery rate: a practical and powerful approach to multiple testing. *Journal of the royal statistical society. Series B (Methodological)* 289–300 (1995).
43. Dwyer, D. B. *et al.* An Investigation of Psychosis Subgroups With Prognostic Validation and Exploration of Genetic Underpinnings: The PsyCourse Study. *JAMA psychiatry* (2020).doi:10.1001/jamapsychiatry.2019.4910
44. Smith, S. M. & Nichols, T. E. Threshold-free cluster enhancement: addressing problems of smoothing, threshold dependence and localisation in cluster inference. *Neuroimage* **44**, 83–98 (2009).
45. Association, A. P. *Diagnostic and Statistical Manual for Mental Disorders*. (American Psychiatric Association: Washington, DC, 1994).
46. Ding, C., Li, T., Peng, W. & Park, H. Orthogonal Nonnegative Matrix T-factorizations for Clustering. *Proceedings of the 12th ACM SIGKDD International Conference on Knowledge Discovery and Data Mining* 126–135 (2006).doi:10.1145/1150402.1150420
47. Schimmelmann, B. G., Michel, C., Martz-Irngartinger, A., Linder, C. & Schultze-Lutter, F. Age matters in the prevalence and clinical significance of ultra-high-risk for psychosis symptoms and

© 2020 Koutsouleris N et al. *JAMA Psychiatry.*

criteria in the general population: Findings from the BEAR and BEARS-kid studies. *World Psychiatry* **14**, 189–197 (2015).

48. Schultze-Lutter, F. & Koch, E. Schizophrenia Proneness Instrument, Children and Youth Version (SPI-CY). (2010).
49. Fusar-Poli, P. *et al.* The dark side of the moon: meta-analytical impact of recruitment strategies on risk enrichment in the clinical high risk state for psychosis. *Schizophrenia Bulletin* **42**, 732–743 (2015).
50. Koutsouleris, N. *et al.* Disease prediction in the at-risk mental state for psychosis using neuroanatomical biomarkers: results from the FePsy study. *Schizophr. Bull.* **38**, 1234–1246 (2012).
51. Quade, D. Using Weighted Rankings in the Analysis of Complete Blocks with Additive Block Effects. *Journal of the American Statistical Association* **74**, 680–683 (1979).
52. First, M. B., Spitzer, R. L., Gibbon, M. & Williams, J. B. W. *Structured Clinical Interview for DSM-IV-TR Axis I Disorders, Research Version, Patient Edition. (SCID-I/P) New York: Biometrics Research, New York State Psychiatric Institute, November 2002*. (Biometrics Research, New York State Psychiatric Institute: New York, Biometrics Research, 2002).

© 2020 Koutsouleris N et al. *JAMA Psychiatry.*
